# Mindfulness-Based Interventions to Implement the Psychological Well-Being of Nursing Students: A Scoping Review

**DOI:** 10.3390/healthcare14010130

**Published:** 2026-01-04

**Authors:** Milena Consorte, Elena Morotti, Fabio Nanni, Alessandro Giannandrea, Stefano Benini, Monica Martoni

**Affiliations:** 1 Clinical Governance, Research, Education and Quality System Unit, Local Health Authority of Bologna, 40124 Bologna, Italy; m.consorte@ausl.bologna.it (M.C.); f.nanni@ausl.bologna.it (F.N.); stefano.benini@ausl.bologna.it (S.B.); 2 Department of Biomedical, Metabolic and Neural Sciences, University of Modena and Reggio Emilia, 41125 Modena, Italy; elena.morotti@unimore.it; 3 Department of Psychology, Sapienza University of Rome, 00185 Rome, Italy; a.giannandrea@gmail.com; 4 Department of Medical and Surgical Sciences, University of Bologna, 40126 Bologna, Italy

**Keywords:** nursing students, undergraduate nursing students, pre-licensure, mindfulness, mindful self- compassion, psychological well-being, satisfaction, self-efficacy, empathy, resilience

## Abstract

**Highlights:**

**What are the main findings?**

- Mindfulness-based interventions (MBIs) are increasingly integrated into nursing education; however, substantial heterogeneity exists in intervention protocols, duration, delivery modes, and facilitator training.
- The majority of studies focus on reducing psychological distress, whereas outcomes related to positive psychological well-being and core nursing competencies remain underexplored.

**What are the implications of the main findings?**

- Future research should broaden outcome assessment to include positive indicators of well-being aligned with the relational and humanistic dimensions of nursing practice (e.g., empathy, compassion satisfaction, self-efficacy).
- The development and curricular integration of theory-informed, population-specific mindfulness and self-compassion protocols may enhance standardization, student engagement, and comparability across studies.

**Abstract:**

**Background/Objectives**: Mindfulness is a meditative practice that increases levels of awareness and attention. Introducing this practice into the curricula of nursing students could improve the relationship with patients and promote students’ well-being. This scoping review aims to map the literature on mindfulness-based interventions (MBIs) administered to nursing students to promote their psychological well-being. **Methods**: The review was conducted according to Peters’ framework. PCC eligibility criteria were used. The PCC mnemonic stands for Population (nursing students), Concept (mindfulness-based interventions applied with positive and negative outcomes for psychological well-being), and Context (undergraduate, pre-licensure). The search was conducted in March 2024 by consulting PubMed, ERIC (Ovid), CINAHL (EBSCOhost), PsycINFO (Ovid), and ProQuest databases. Additional sources have been identified from Google Scholar. **Results**: Of the 763 articles that emerged, 55 were included in the scoping review. The interventions depicted are heterogeneous in terms of content and timing. The most investigated outcomes remain stress and anxiety; self-efficacy and empathy emerge as positive indicators of psychological well-being. In the post-COVID period, there is an increase in the online delivery method. **Conclusions**: In order to provide guidance for practice and institutional policies, future research could focus on a systematic review that identifies the most appropriate MBIs for the well-being of nursing students. In addition, it would be useful to conduct feasibility studies to introduce structured mindfulness-based practices into the nursing degree programmes, with standardized and adaptable pathways tailored to the needs of different academic realities.

## 1. Introduction

For nursing students, the concept of “self-care” is associated with Dorothea Orem and her self-care theory. From a universal perspective, nursing care ideally aims to promote the patient’s full intellectual, emotional, spiritual, and physical well-being [1]. However, caring for others requires nurses to invest a significant physical, cognitive, emotional, and relational resources. For this reason, the construct of “self-care” should be understood as having a double value, encompassing both intra and interpersonal dimensions, because one’s ability to take care of others effectively is closely connected to one’s ability to care for oneself [2].

Over the years, the concept of self-care has become increasingly important in the nursing profession, as the extent to which this population is subject to repeated stressors has become apparent [3,4,5]. This exposes nurses to the risk of developing an increasing degree of compassion fatigue [5,6,7], which can negatively affect not only their health, but also the quality of care provided [8,9]. This reflection transforms the need to reduce the risk of psychological distress and compassion fatigue into an ethical and deontological duty. Care must be focused first and foremost on the professional, in order to preserve and renew the energy that will be expended in the process of caring for those receiving assistance. In other words, only by reserving a space for ourselves will we be able to welcome the being of others [1].

In this regard, the great intuition of theorist Jean Watson, founder of the Human Caring theory, is precisely to address the concept of care within this dual vision. Watson places care at the centre of her thinking. In her theory, Watson identifies a deep correlation with the thinking of important philosophers such as Heidegger, who defines care as a fundamental ontological phenomenon [10], and Levinas, with his Ethics of Belonging [11,12]. Building on this philosophical foundation, Watson emphasizes that caring for oneself is inseparable from caring for others. This conceptual bridge paves the way for self-care practices that resonate with mindfulness, understood as an attitude of presence and compassion in the here and now [13].

The importance of these practices becomes even more evident when considering that nursing students are subjected to higher levels of stress than their peers in other disciplines, mainly due to academic demands and clinical placements [14]. Since the COVID-19 pandemic, the importance of self-care has become even more apparent. Among the various self-care practices, mindfulness-based interventions (MBIs) introduced as part of university courses have consistently shown positive effects on students’ psychological well-being [15]. Mindfulness has been operationally defined as “the awareness that arises from paying attention in a particular way: intentionally, in the present moment, and non-judgmentally” [16]. A multicenter cross-sectional study conducted in Northern Italy in 2020 reported that approximately 70% of nursing students experienced significant psychological distress. However, students with higher dispositional mindfulness (DM) showed lower levels of distress. Based on these findings, the authors suggested integrating mindfulness courses specifically tailored for nursing students in their curricula as a protective factor against stress [17]. In addition, dispositional mindfulness is associated with key relational competencies. Higher DM levels are positively correlated with perspective taking and empathic concern and negatively related to personal distress. Nursing students with higher levels of DM appear better able to understand others’ perspectives, experience compassion, and manage emotional discomfort in challenging interpersonal situations. Consequently, ad hoc mindfulness intervention may support the development of functional empathy within university programmes [18].

Mindfulness practices involve intentionally focusing attention on the present moment, including breathing, body sensations, thoughts and emotions, and the environment around us. This attentional focus reduces ruminative thinking about the past and anxiety-provoking anticipations of the future, such as concerns related to academic and clinical performance. Present moment awareness fosters an approach to care grounded in listening, acceptance, and empathy, thereby enhancing the quality of the helping relationship [19]. Mindfulness-based interventions (MBIs) are recognized as a protective factor against psychological distress associated with clinical practice and academic demands in nursing education. Their relevance extends beyond stress reduction, as they align with the evolving nursing paradigm that emphasizes holistic and person-centred care rather than a purely technical approach [20]. In this context, mindfulness is closely related to self-care and has been linked to constructs such as resilience and compassion [21]. Despite a growing interest in MBIs, from an initial analysis of the literature, studies investigating the effects of mindfulness interventions on nursing students refer exclusively to stress and anxiety. Other dimensions of psychological well-being, such as self-efficacy, empathy, resilience, emotional intelligence, and compassion satisfaction have received limited attention.

As of December 2023, preliminary research conducted on MEDLINE, the *Cochrane* Database of Systematic Reviews, and Joanna Briggs Institute Evidence Synthesis databases identified no systematic reviews specifically addressing mindfulness-based interventions aimed at promoting psychological well-being among nursing students. Two systematic reviews closely related to this topic were identified, but both had a narrower or different focus. The review by Aloufi et al. [22] examined MBIs primarily in relation to the reduction in stress, anxiety, and depressive symptoms among undergraduate nursing students [22]. While demonstrating the potential effectiveness of MBIs in managing psychological distress, the review focused almost exclusively on symptom reduction and did not explore broader dimensions of psychological well-being or positive psychological outcomes. Similarly, the Cochrane review by Kunzler et al. [23] investigated psychological interventions designed to foster resilience among healthcare students [23]. However, this review included a wide range of heterogeneous interventions, with mindfulness-based approaches representing only one component, and did not provide a specific or comprehensive analysis of MBIs or their multidimensional outcomes. Furthermore, the population was mixed and did not include only nursing students. Consequently, an important gap remains in the literature regarding how mindfulness-based interventions have been specifically applied to nursing students and which outcomes related to psychological well-being have been investigated beyond stress and anxiety.

A scoping review (ScR) is therefore warranted to describe the types of mindfulness-based interventions implemented in this population. This scoping review aims to map all examined psychological well-being outcomes—both positive and negative—and the assessment tools used. Addressing this gap will contribute to a more comprehensive understanding of the role of mindfulness in nursing education and inform future research and educational programme development.

## 2. Methods

The research was conducted adhering to the Preferred Reporting Items for Systematic reviews and Meta-Analyses extension for Scoping Reviews—PRISMA-ScR- guidelines (Supplementary File S1: PRISMA checklist) and in accordance with Peters’ framework, as indicated by the methodology of the Joanna Briggs Institute [24,25,26].

The objectives, inclusion criteria and methods were specified in an a priori scoping review protocol registered on the Open Science Framework (https://osf.io/) on 10 March 2024, available at the link https://osf.io/7ek8d.

### 2.1. Review Question(s)

- Primary question:
  - Which mindfulness-based interventions (MBIs) are administered to nursing students to implement their psychological well-being (e.g., increased level of awareness, resilience, empathy, emotional intelligence, self-care, compassion satisfaction, compassion fatigue, reduced stress, anxiety, and self-efficacy)?
- Sub-questions:
  - Are the periods in which they are administered (academic year, internship, theoretical lessons, laboratory activities) specified?
  - Are any adverse events reported in studies that included mindfulness-based interventions for nursing students?
  - What measurement tools are given to students to assess the outcomes of mindfulness-based interventions (MBIs)?
  - Are the competencies of the mindfulness facilitator administering the intervention specified?

### 2.2. Eligibility Criteria

PCC eligibility criteria were used. The PCC mnemonic stands for Population, Concept, and Context [26].

Population: The scoping review considered studies that refer to pre-licence and undergraduate nursing students. Studies that refer to undergraduate students of other degree programmes (medicine, psychology, physiotherapy, obstetrics and the like), postgraduate nursing students, or master’s and doctoral degree programmes in Nursing were excluded.

Concept: The scoping review considered studies that refer to all types of mindfulness-based interventions, and included mindful self-compassion, teaching methodologies, and tools (face-to-face workshops, online programmes, seminars, interventions, or self-study programme formats). In addition, the included studies had to describe the outcomes obtained and the competencies of the MBIs trainer. Descriptive studies of students’ mental health that only suggest the need for new approaches to implement their well-being were excluded.

Context: The scoping review considered studies that relate to bachelor and pre-licensure programmes in Nursing. International studies from all countries, written in English, with an abstract and full text, that encompass the eligibility criteria of the scoping review were included.

#### Types of Sources

Experimental and quasi-experimental studies were included, as well as qualitative and mixed methods studies in which mindfulness-based interventions were administered to undergraduate nursing students. In addition, grey literature, i.e., dissertations and theses, which adhered to the eligibility criteria of the scoping review was also considered. Systematic reviews were excluded but their reference sections were analyzed in order to identify relevant studies that did not emerge from the search strategy.

### 2.3. Search Strategy

In January 2024, the first phase of the search was conducted on MEDLINE (PubMed) and CINAHL (EBSCO) to identify topic-related titles and abstracts according to the PCC framework. Text words from relevant abstracts were selected and evaluated to identify keywords. The search string was adapted accordingly for each database (Appendix A).

The second phase of the search was conducted from 11 March to 15 March 2024, in the databases PubMed, ERIC (Ovid), CINAHL (EBSCOhost), PsycINFO (Ovid), and ProQuest. In the stated time frame, the search for other sources also included Google Scholar (years > 2020 for feasibility reasons). The following limits were applied: abstract and full text available, English language. Articles published after 15 March 2024, were not included.

The third phase of the search examined the references section of reviews to include articles, with abstracts and full text, that did not emerge from the database searches. When clarification related to the PCC was required, the author of the article was contacted; the specified response time was seven days; failure to respond within this time frame resulted in exclusion of the article.

### 2.4. Study/Source of Evidence Selection

**Figure 1:**
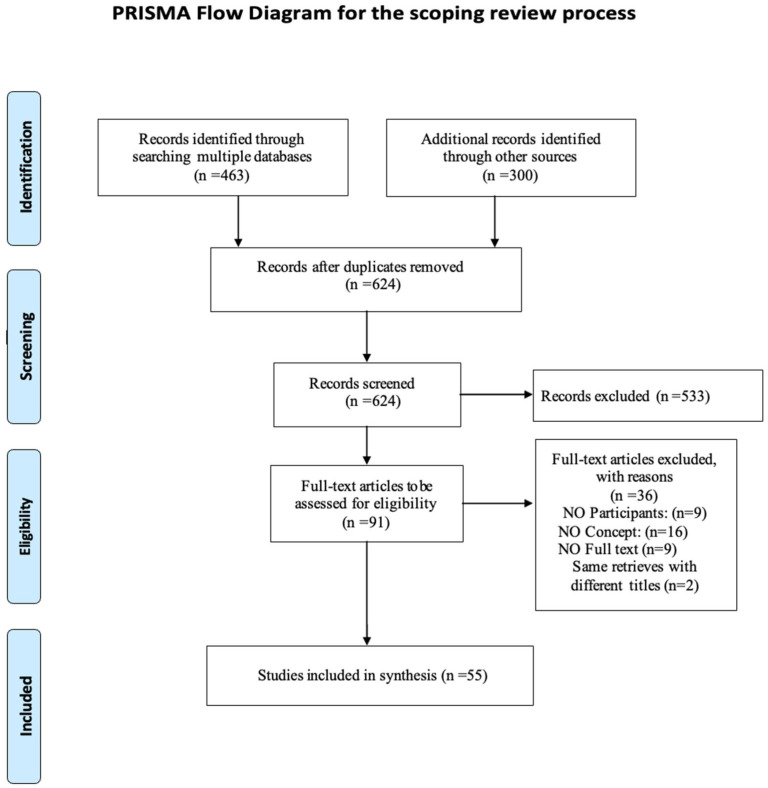
PRISMA Extension for Scoping Review (PRISMA-ScR) that illustrated the selection of sources of evidence.

The selection process is reported in the PRISMA-ScR-Preferred Reporting Items for Systematic Reviews and Meta-analyses extension for scoping review flowchart [24], including the reasons for the exclusion of the articles which did not meet the inclusion criteria (Figure 1, Section 3).

All identified records were collected in a Microsoft Excel spreadsheet and duplicates were removed. Each title and abstract (TI/AB) was then independently screened by the three reviewers (M.C., E.M., F.N.) for evaluation against the inclusion criteria. Potentially relevant items were recovered in full. The subsequent full-text screening phase, conducted by the three reviewers (M.C., E.M., F.N.), further reduced the number of articles included. Disagreements that arose between the reviewers, at any phase of the selection process, were resolved through discussion or by requesting the intervention of an additional reviewer (M.M.).

### 2.5. Data Extraction

For the data summary, the data extraction table presented in the protocol has been partially modified: the section dedicated to secondary objectives (not explained in the studies) has been removed; the publication country, conclusions, and limitations have been added.

### 2.6. Data Analysis and Presentation

Graphs were used as synthesis tools, in particular for data related to the distribution of sources by country of origin, outcomes, and the most frequent assessment tools. A descriptive summary accompanies the graphical results and outlines how the results relate to the objectives and questions of the scoping review.

## 3. Results

### 3.1. Search Results

Of the 763 articles that emerged, 55 were included in the scoping review. The PRISMA—for scoping review flowchart (Figure 1) shows the screening process.

After the removal of duplicates, three independent reviewers (M.C., E.M., F.N.) evaluated the titles and abstract (TI/AB). At this stage, most of the excluded articles were cross-sectional studies that correlated dispositional mindfulness with outcomes associated both positively and negatively with the psychological well-being of nursing students. Other excluded articles concerned mixed populations or interventions that were not truly mindfulness based. The subsequent selection of the full texts further reduced the number of articles as they did not fully meet the eligibility criteria.

During the third phase, which involved reviewing the references of systematic reviews excluded from this scoping review, two relevant articles were identified. These were subsequently included among the records from other sources, which primarily consist of doctoral theses.

### 3.2. Inclusion of Sources of Evidence

Some articles did not provide complete answers to the scoping review questions; therefore, one reviewer (M.C.) contacted the authors by email to request additional information. The authors of the doctoral dissertations could not be contacted because no email address for correspondence was available. For journal articles, e-mails were sent to the corresponding authors, to request clarification in relation to the scoping review questions. Of these, nine out of 29 responded within the specified time frame of one week [27,28,29,30,31,32,33,34,35]; for three authors, the correspondence email address was not valid [36,37,38]. In order to gain broader mapping, however, the reviewers decided to include all sources, as the missing information pertained to secondary questions only.

### 3.3. Review Findings

In this section, the aggregate results are presented in narrative, tabular and graphic form. The data were extrapolated from the data extraction table (see Supplementary File S2: DATA EXTRACTION TABLE ScR) and presented in tabular or graphic form by one of the authors (M.C).

#### 3.3.1. Study Characteristics

In the ScR we have included 37 journal articles (67%) [19,27,28,29,30,31,32,33,34,35,36,37,38,39,40,41,42,43,44,45,46,47,48,49,50,51,52,53,54,55,56,57,58,59,60,61,62] and 18 doctoral dissertations (33%) [63,64,65,66,67,68,69,70,71,72,73,74,75,76,77,78,79,80]. Of the studies included, most were conducted in the United States of America. The origin of all the articles is shown in Table 1.

As for the distribution by year, an increase from the year 2022 onwards was evident. This increase could depend on two factors: the pandemic period which induced a focus of attention on certain topics; Google Scholar research that was conducted within a time limit (>2020) for feasibility reasons.

Among the study designs we find a prevalence of quasi-experimental studies (40%) [29,34,35,47,48,49,50,52,53,55,59,60,61,62,64,67,70,71,73,76,78,79] and Randomized Controlled Trials or RCTs (38%) [31,33,36,37,38,39,40,41,42,44,46,51,54,56,57,63,66,68,69,72,80]; Mixed-Method studies appear for 16% of sources [19,27,28,32,58,65,74,75,77]; qualitative studies for 5% [30,43,45].

#### 3.3.2. Participants in the Studies

In relation to the number of participants, the panorama of articles is very varied: the sample size ranged from five [34] to 201 students [19]. Regarding the doctoral dissertations the sample size ranged from nine [71] to 203 [70]. The total of participants who began the studies is 4320, and the total of participants who did not complete required activities or withdrew before the end of the studies is 799. The problem of dropout rate is common to many studies. This may be attributed to the low adherence of students due to commitments.

#### 3.3.3. Mindfulness-Based Interventions Applied

The MBIs found in the studies are numerous and differ in terms of type, investigated outcomes, duration and mode of delivery. Thirteen studies are inspired by the MBSR, Mindfulness-Based Stress Reduction [27,31,35,38,51,52,54,59,60,67,76,78], lasting eight weeks, and two of these were carried out in online mode [54,59]. Two studies applied Koru Mindfulness Meditation [66,77], which is based on practices such as diaphragmatic breathing, breath awareness, dynamic breathing, body scanning, mindful walking, Gatha meditation, guided imagery, and thought/feeling labelling. Among the remaining studies, four focused on heterogeneous Mindful Self Compassion (MSC) protocols, whereas the others proposed interventions based on Mindfulness Meditation are proposed. See Table 2 for further details.

In the included studies there are some interventions (30%) lasting longer than 8 weeks [27,30,31,32,37,38,42,48,52,59,62,68,79,80], 10 weeks [40], or 12 weeks [49,51] and many are shorter in duration. The most common interventions are unstructured and involve sporadic practices implemented in preparation for an examination or a lesson. Shorter interventions are often related to self-control before a cognitive or motor performance and last from two minutes or less [33,70], or three minutes [58,63], up to nine hours over two days [50].

#### 3.3.4. Outcomes

**Figure 2:**
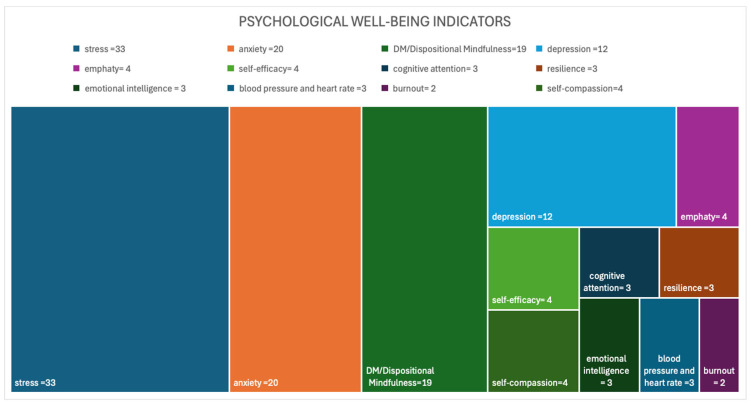
Most frequently investigated outcomes in the reviewed studies. Each colour indicates a specific psychological outcome, and the associated number represents the number of times a specific outcome has been considered in the selected articles.

The most frequently investigated indicators remain those negatively associated with psychological well-being: stress (30.0%), anxiety (18.2%) and depression (10.9%). Dispositional mindfulness is the only positive indicator that is investigated quite frequently; it appears 19 times (17.3%) in the research included in the scoping review. Figure 2 describes the most frequently investigated outcomes in the studies.

#### 3.3.5. Assessment Tools

**Figure 3:**
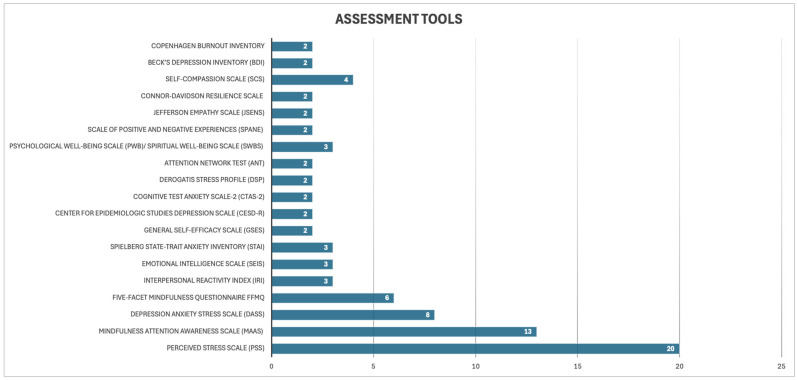
The bars indicate the number of times a specific tool has been used to measure related psychological variables in all reviewed articles.

The results are followed by measurement scales. The most frequently used scales in the studies included are the Perceived Stress Scale (PSS), which measures perceived stress levels [81]; the Mindfulness Attention Awareness Scale (MAAS) for dispositional mindfulness [82]; the Depression Anxiety Stress Scale (DASS) that measures depression, anxiety, and stress, which are often associated with each other [83]. PPS and MAAS were used 20 and 13 times, respectively. Figure 3 descrbes the assessment tools that are most frequently used in studies.

A comprehensive overview of the study characteristics is presented in Table 2, including first author, year of publication and country, study design, sample Size and dropout rate, experimental group intervention, control group intervention, outcome measures (variables), finding and publication type.

#### 3.3.6. Delivery Method of Mindfulness-Based Interventions

In included studies, the most common delivery method of MBIs is face-to-face [19,30,33,36,37,39,40,43,44,45,48,50,56,58,60,62,67,75,80]. There is a significant propensity for face-to-face interventions with Supplementary Materials for home practice [27,29,31,34,35,38,42,51,52,53,68,72,76]. However, a shift toward online delivery is evident, especially in publications after 2021, a trend that was probably due to social restrictions during the COVID pandemic. In fact, 15 out of 18 [28,32,46,49,54,55,57,61,64,65,70,73,74,77,79] studies, the total of those applying online modalities, were conducted from 2021 to 2024 (compared to three studies with online interventions before 2021) and of these only five sources come from Google Scholar (for the time filter applied).

**Figure 4:**
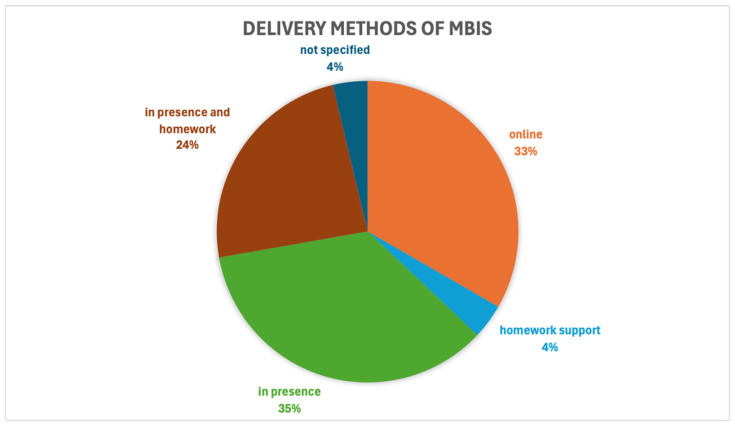
The percentage of mindfulness-based interventions (MBIs) used in-person.

In addition to online MBIs, protocols built ad hoc for the population examined, administered on IT platforms (institutional and non-institutional) and applications for smartphones and videos on YouTube were offered. The different delivery methods are represented in Figure 4.

#### 3.3.7. Competencies of the Facilitators of Mindfulness-Based Interventions

**Figure 5:**
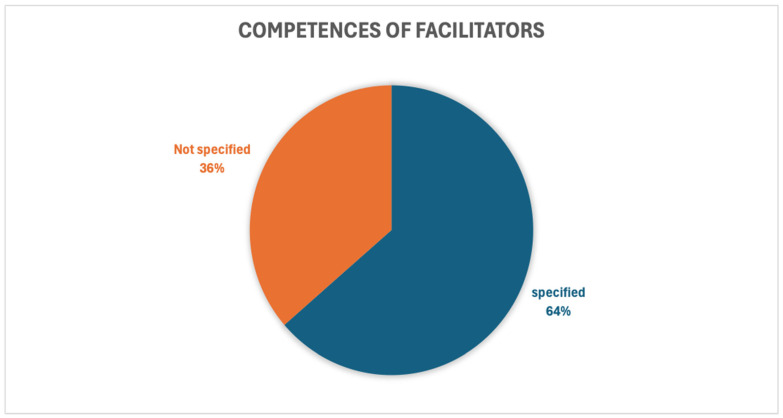
Percentage of studies reporting specified versus unspecified facilitator competencies.

Albeit very heterogeneous, the competencies of the facilitators involved in mindful-based interventions are specified in most studies (64%), as shown in Figure 5. Out of a total of 55 studies, 19 did not specify the level of competence of the facilitator [19,27,39,40,47,53,55,56,58,59,63,64,69,73,74,75,77,78,79]; of these studies, seven were administered online [55,59,64,73,74,77,79]. In most of the remaining 36 studies, the trainers were mindfulness facilitators with certification at different levels and personal experience of practice, with the exception of three studies in which they were psychiatrists [46,52,66], two sources in which they were psychologists [36,50], and two articles in which they were cognitive behavioural therapists [51,54].

#### 3.3.8. Adverse Events

In the papers included in this ScR, two articles examined this topic and ruled out the presence of adverse events [50,71]. However, one of these articles reported a possible risk of emotional dependency among students [50]. For the other articles, eight authors provided additional information in response to our e-mail request for clarification. They reported that participants did not experience any significant adverse events [28,29,30,31,32,33,34,35]. In one clarification, it was noted that some students fell asleep during the practice [30]; in another, a student, who described himself as generally anxious, reported increased awareness accompanied by elevated stress levels. Despite this adverse experience, the student continued and completed the course [35]. In the latter case, the intervention was an adapted MBSR programme, consisting of two-hour sessions held twice a week for four weeks. The remaining papers did not address the issue of adverse events related to the mindfulness-based intervention.

#### 3.3.9. Intervention Characteristics

Table 3 presents the intervention characteristics of the included studies, including MBIs duration, mode of delivery, links to website or mobile apps, reported adverse events, facilitators’ competence, and the context in which the interventions were applied.

## 4. Discussion

The importance of integrating a mindfulness-based approach into nursing curricula has been clearly and exhaustively explained by Beddoe & Murphy [27], who frame mindfulness as a response to core questions in nursing education: how we care for ourselves, how we compassionately care for others, and what values should guide professional training. Central to this perspective is the recognition that compassion which excludes oneself is incomplete [84]. In line with this framework, the findings of the present scoping review suggest that MBIs offer added value to the education of nursing students. However, the review also highlights substantial heterogeneity in the types of interventions implemented, and in the outcomes assessed. In the studies included in this ScR, the predominant indicators are always those associated with psychological distress, such as stress, anxiety, and depression. Variables that are positively associated with psychological well-being are investigated in only 37.3% of research. This result is in line with previous systematic reviews conducted in the same population of interest [22,85,86]. Having extensively studied the effects of MBIs on distress reduction, future research should broaden its focus to outcomes that are more closely aligned with the relational and humanistic dimensions of nursing practice.

Carl Rogers, founder of the Theory of Counselling, summarized the main qualities for person-centred care—empathy, active listening skills, and authentic presence—that are closely connected to mindfulness and positive mediators of psychological well-being. These qualities may buffer against compassion fatigue. An authentic helping relationship enhances compassion satisfaction (ProQOL manual https://proqol.org/proqol-manual; accessed on 22 November 2025), with a direct impact on professional well-being and quality of the care provided. In this context, the inclusion of Mindful Self-Compassion in nursing curricula appears particularly relevant. Nevertheless, only two studies introduce MSC interventions [54,86]. Furthermore, the protocols adopted were not comparable, limiting conclusions regarding their effectiveness.

Another critical issue emerging from this ScR concerns the mode of delivery (in-person or online) and intensity of MBIs. Many interventions were designed as brief online programmes to increase accessibility and reduce student dropout [45,59]. Brief bursts of MBIs administered through devices, however, generated conflicting results. In fact, it is difficult for educators to try to calibrate the duration of the protocols that they should offer, particularly in light of clinical and academic commitments. These findings are in line with those of Jimènez et al. [87], who showed that low-intensity MBIs (lasting between 8 and 15 min per meeting) can produce statistically significant effects on outcomes such as rumination, distraction, and impulse control [88,89]. The review concludes with the suggestion that new research should be carried out on the effects of short MBIs on larger samples so that these techniques can be incorporated into clinical practice. This conclusion leads to the third critical issue found in this ScR: most studies relied on small and convenient samples. In addition, most of them lacked follow-up assessment to evaluate the maintenance of the outcomes obtained and their impact on the quality of care.

The issue of adverse events also warrants attention. The majority of studies included in this review did not report negative outcomes, particularly when low-intensity interventions were used. The isolated reports of increased stress or discomfort were associated with higher-intensity programmes. Consistent with the work of Baer et al. [90], the safety of MBIs appears to depend on practice intensity, participant vulnerability, and facilitator expertise. This finding underscores the importance of adequately trained instructors and careful adaptation of intervention intensity to the student population.

Consistent with the work of Baer et al. [90], of the Oxford Mindfulness Centre, the safety of MBIs appears to depend on practice intensity (expressed in terms of daily commitment required and exploratory depth of self), intrinsic factors of the participant (susceptibility to frequent manifestations of psychological distress or recurrent depression), and intrinsic factors of the trainer, such as experience, professionalism, the ability to lead a group, and the embodiment of meditative practice. Baer et al. [90] identify low-intensity interventions as those that direct attention to the capacity for awareness in sensory perception. Generally, these types of MBIs are not associated with personal injury. Their research does not show adverse events linked to moderate-intensity practices, although research in this field is still in its infancy. MBSR or MBCT programmes can be considered moderate-intensity interventions, and it would be desirable for them to be conducted by experienced professionals (e.g., psychologists, psychotherapists) in order to manage the possible occurrence of adverse events. The use of the conditional is necessary, given the wide variability in trainers’ expertise across current programmes. High-intensity practices are defined as residential meditation retreats where generous commitment is required from participants, such as prolonged silences and daily meditations lasting hours. In line with the findings of the review by Baer et al. [90], facilitator training also varied greatly across the sources included. This variability further complicates comparisons between interventions and highlights the need for clearer reporting standards in future research. Despite these limitations, there is broad consensus across the literature on the value of integrating MBIs into nursing curricula, not only to mitigate academic and clinical stress, but also to enhance compassion satisfaction and professional well-being.

### 4.1. Intervention Characteristics and Outcomes of MBIs

In relation to the intervention characteristics summarized in Table 1 and Table 2, the effectiveness of mindfulness-based interventions (MBIs) appears to vary systematically according to programme structure, duration, and facilitator competence. More structured and longer interventions, such as Mindfulness-Based Stress Reduction (MBSR), Mindfulness-Based Cognitive Therapy (MBCT), and Koru-based programmes, are more frequently associated with statistically significant improvements in stress, anxiety, emotional regulation, empathy, and self-care outcomes [27,29,31,35,38,44,51,52,66,80]. These effects are predominantly reported in studies involving trained or experienced facilitators and sustained engagement over several weeks, often combined with regular home practice [44,51,52,66]. However, such interventions also entail higher demands in terms of time commitment and organizational resources, which may limit their feasibility and attractiveness within academic curricula [27,51,52].

In contrast, mindfulness meditation interventions centred on sensory awareness and brief guided practices show more heterogeneous results. While several studies report statistically significant improvements in stress, anxiety, or dispositional mindfulness [36,39,40,41,46,55,61,69,75], others yield non-significant quantitative findings despite positive qualitative feedback, including increased calmness, attentional focus, and emotional centring [19,28,32,43,65]. These interventions appear to be more adaptable in terms of duration and delivery format, including online and hybrid modalities, potentially enhancing accessibility within nursing education programmes [32,41,46,49,64].

Very brief MBIs administered immediately before academic activities or examinations demonstrate mixed effectiveness. Although these interventions are highly feasible and time-efficient, quantitative outcomes are often modest or non-significant [28,63,65,70,73], possibly due to insufficient depth of practice and implementation challenges, such as limited faculty engagement and variable student adherence. Overall, the evidence summarized in Table 1 and Table 2 suggests that the effectiveness of MBIs in nursing education is not inherent to mindfulness practice alone, but is closely moderated by intervention intensity, facilitator expertise, delivery context, and opportunities for sustained practice.

### 4.2. Limitation of Scoping Review

This scoping review presents some methodological limitations. First, the data extraction tool was refined after the review process had commenced, as the original version proposed in the protocol had not been piloted. The modifications were necessary to adequately capture the heterogeneity of mindfulness-based interventions and outcomes identified and were transparently reported in line with JBI guidance. Second, the literature search was completed in March 2024; therefore, studies published thereafter were not included, and more recent evidence may not be represented in the present review. This temporal limitation highlights the need for future updates of the evidence as the field continues to evolve. In addition, filters such as restriction to the English language and a temporal filter for Google Scholar (>2020) were applied for feasibility reasons. This may have reduced the number of sources included in this ScR.

A further limitation of this scoping review is that, in accordance with JBI and PRISMA-ScR guidance, it did not aim to evaluate the effectiveness of mindfulness-based interventions. Consequently, the review provides a descriptive synthesis of reported outcomes rather than a critical appraisal or effect-size estimation. While this approach is consistent with the purpose of scoping reviews, it highlights the need for future systematic reviews and meta-analyses to assess the effectiveness of MBIs once more methodologically homogeneous studies become available.

### 4.3. Methodological Limitations of the Existing Literature

This review identified several methodological limitations that constrain the interpretability and transferability of current evidence. Most studies relied on small, non-probabilistic convenience samples, reported high dropout rates, and lacked follow-up assessments to evaluate the sustainability of outcomes over time. In addition, outcome measures were predominantly self-reported, limiting the ability to assess the impact of MBIs on observable clinical or educational outcomes.

Another relevant limitation concerns the heterogeneity of intervention protocols, delivery formats, and facilitator training. This variability complicates comparisons across studies and limits the identification of best practices. Furthermore, although most studies reported no adverse events, the inconsistent reporting of negative or unintended effects represents an additional methodological weakness.

### 4.4. Implications for Education and Practice

The findings of this scoping review highlight the growing integration of MBIs within nursing education, while also revealing a lack of conceptual alignment between intervention objectives and the core values of the nursing profession. Although MBIs are predominantly implemented to reduce psychological distress, and their potential to foster positive psychological resources—such as empathy, self-efficacy, compassion, and emotional intelligence—remains underutilized.

From an educational perspective, these findings suggest that MBIs should be more explicitly framed as formative tools supporting professional identity development, relational competence, and sustainable self-care, rather than solely as stress-management strategies. Integrating mindfulness and mindful self-compassion within curricula may contribute to preparing students for emotionally demanding clinical environments and enhance compassion satisfaction, with potential downstream effects on quality of care.

The integration of self-care and mindfulness-based interventions directly into undergraduate nursing curricula may represent a key strategy for enhancing student engagement and adherence. Voluntary or extracurricular participation may be associated with variable attendance and inconsistent practice, whereas curricular integration can normalize self-care as a core professional competence and promote sustained engagement over time. Moreover, the analysis of intervention characteristics indicates that programmes adopting a structured and theory-informed approach to self-care activities are more consistently associated with positive outcomes across psychological, emotional, and professional domains.

These observations highlight the potential value of developing a population-specific protocol tailored to nursing students. An ad hoc protocol could contribute to greater standardization of intervention components (e.g., duration, intensity, facilitation, and practice requirements), thereby improving comparability across studies and strengthening the overall evidence base. In addition, a structured, curriculum-embedded protocol may support adherence by aligning self-care practices with educational objectives and assessment frameworks, reinforcing their relevance within professional training rather than positioning them as optional or ancillary activities.

### 4.5. Implications for Future Research

The gaps identified in this scoping review indicate several priorities for future research. Among these is the need to broaden the domains of outcomes beyond psychological distress to include positive indicators of well-being in line with nursing competencies (e.g., empathy, compassionate satisfaction, self-efficacy, emotional intelligence). In addition, it will be useful to conduct research with greater methodological rigour, using larger samples and longitudinal designs with follow-up assessments to examine the maintenance of outcomes and their impact on educational and clinical practice. Consequently, it is appropriate to optimize the measurement of outcomes by integrating self-reported data with observational or patient-reported measures, particularly with regard to compassionate care. The characteristics of the intervention must be clear in future research, including intensity, duration, mode of delivery, and facilitator training, to support reproducibility and implementation. Feasibility and acceptability must be assessed at the organizational level, addressing institutional readiness, cultural barriers, and resource implications in nursing education. It is important to emphasize that, once a sufficiently homogeneous body of evidence is available, future research projects, such as systematic reviews or meta-analyses, will be needed to evaluate the effectiveness of MBIs.

## 5. Conclusions

The aim of this ScR was to map the existing literature on mindfulness-based interventions designed for nursing students and their association with psychological well-being. The findings reveal substantial heterogeneity in intervention types, delivery formats, and outcome measures. Most studies focused on reducing psychological distress, while indicators positively associated with well-being, such as self-efficacy, empathy, compassion, and emotional intelligence, remain underexplored. Future research should prioritize these outcomes, as they reflect core competencies of the nursing profession. Expanding the evidence base on MBIs will support their integration within university programmes.

However, successful implementation requires addressing cultural and organizational barriers. In order for both educators and students to invest in and benefit from self-care practices, it is important that they feel confident that they can take care of themselves during work and study, and that they can also feel free to share emotional difficulties among peers, dispelling the fear that adherence to such practices could influence the achievement of goals. Unfounded concerns that associate self-care practices during study or work with lack of productivity must be refuted. If these factors are not addressed, engagement in the programme may be jeopardized [91]. Consistent with Waddell et al. [92], existing studies primarily target individual-level factors, often neglecting organizational and systemic dimensions. Therefore, future research should include feasibility studies to assess institutional readiness, educator and student attitudes, perceived barriers, and preferred delivery formats. Understanding these factors is essential to identifying which MBIs are most appropriate and sustainable within nursing education. The road to future research, therefore, still has a long way to go.

## Figures and Tables

**Table 1:** Distribution of sources by Country in alphabetical order. Percentage values have been rounded up or down.

Country	No (%)	Country	No (%)
USA-United State of America [27,28,29,34,43,49,55,56,58,59,63,64,65,66,67,68,69,70,71,72,73,74,75,76,77,78,79]	27 (49%)	Italy [44]	1 (2%)
Turkey [35,42,51,54,57,60,62]	7 (13%)	Hawaii [41]	1 (2%)
China [30,31,32,36,46,80]	6 (11%)	Marocco [52]	1 (2%)
Egypt [47,48]	2 (4%)	Oland [19]	1 (2%)
Jordan [39,40]	2 (4%)	Spain [61]	1 (2%)
Korea [37,38]	2 (4%)	Thailand [56]	1 (2%)
Canada [33]	1 (2%)	UK-United Kingdom [50]	1 (2%)
Indonesia [53]	1 (2%)		

**Table 2:** Study characteristics, with particular reference to first author, year of publication, country in which the study has been conducted, study design, sample size (and relative dropout), mindfulness-based intervention, control condition, outcomes measured, main results, and type of publication.

No.	First Author (Year) Country	Study Design	Sample Size Dropout Score	MBIs	Control	Outcome Measures (Variables)	Finding	Publication Type
1	Niessen (2015) Oland [19]	MM	201 E: 123; C: 78 Dropout rate (17.91%) E: −28 C: −8	Mindfulness training	Wait list	QN: FFMQ-SF (Dispositional mindfulness); SCS^1^ (Self-compassion); QL: Inquiry (students’ perception)	QN: No statistically significant difference QL: MBIs benefited the students in personal and professional life.	Journal Article
2	Beddoe (2004) USA [27]	MM	23 Dropout rate −7 (30%)	MBSR in presence and audio guide recorded at home	-	IRI (empathy); DSP (stress)	Significant reduced students’ anxiety. Reduced levels of anxiety and discomfort; improved levels of empathy and imagination.	Journal article
3	Bultas (2021) USA [28]	MM	48 E: 24; C: 24 Dropout rate (18.75%) E: −9 C: 0	Short Mindfulness Meditation exercise online	Usual care	QN: MAAS (Dispositional Mindfulness); PSS (Stress); CD-RISC-10 (resilience); QL: Inquiry (students’ perception)	QN: An improvement in outcomes was observed but was not statistically significant. QL: greater sense of calm, confidence, awareness	Journal article
4	Burner (2023) USA [29]	QE	67 Dropout rate −9 (13%)	Mindfulness Meditation and MBSR	-	PSS (Stress); MSCS (Self-care)	Statistically insignificant reduction in stress. Statistically significant impact on self-care after the practice. MSCS level decreased in follow-up	Journal article
5	Chiam (2020) China [30]	QL	32 Dropout rate −12 (37.5%)	Mind-Nurse Program	-	Inquiry (students’ perception)	MBIs benefited the students in personal and professional life.	Journal Article
6	Kou (2022) China [31]	RCT	60 E: 30; C: 30 Dropout rate (3.33%) E: −1; C: −1	MBSR MBCT	Lecture on Mindfulness	SCS^2^ (Communication skills); EIS (Emotional intelligence); CAI (Human care)	Statistically significant improvement of the investigated outcomes	Journal Article
7	Lam (2024) China [32]	MM	126 Dropout rate −15 (12%)	Mindfulness-based PAL		OLBI-S (Burnout); DASS-21 (Depression, anxiety, stress); GSES (Self-efficacy)	QN: Statistically insignificant reduction in depression, anxiety and stress. Statistically significant impact on self-efficacy and burnout. QL: MBIs benefited the students in personal and professional life.	Journal Article
8	Pollard (2020) Canada [33]	RCT	120 E and C not specified Dropout rate −13 (11%) E: 86; C: 21	Mindfulness moment before clinical simulation	No intervention	National Aeronautics and Space Administration Task Learning Index (mental demands, physical demands, time demands, effort, performance, and frustration)	An improvement of workload demanded in two domains (temporal and exertion).	Journal Article
9	Schwarze (2015) USA [34]	QE	5 Dropout rate −2 (40%)	MBCT Recorded guide on Podcast	-	MAAS (Dispositional Mindfulness); PSS (Stress)	An improvement of the investigated outcome	Journal Article
10	Uysal (2022) Turkey [35]	QE	71 E and C: not specified Dropout rate −19 (27%) E: 17; C: 35	MBSR	No intervention	PSS (Stress); PPSRS (student’s physical, psychological, and social health); MAAS (Dispositional Mindfulness)	Statistically significant improvement of the investigated outcomes	Journal Article
11	Chen (2013) China [36]	RCT	60 E: 30; C: 30	Mindful breathing	No intervention	SAS (anxiety); SDS (depression); Physiological Measures: (Heart Rate; Blood Pressure)	A reduction in anxiety levels and blood pressure was observed.	Journal article
12	Kang (2019) Korea [37]	RCT	41 E: 21; C: 20 Dropout rate (21.95%) E: −5; C: −4	Stress Coping Program MBIs	Lecture on stress and coping	PWI-SF (Stress); STAI (Anxiety); BDI (Depression) Physiological Measures (Heart Rate; Blood Pressure)	Statistically insignificant reduction in depression, heart rate and blood pressure. Statistically significant impact on anxiety and stress.	Journal Article
13	Song (2015) Korea [38]	RCT	50 E: 25; C: 25 Dropout rate (12%) E: −4; C: −2	MBSR	Wait list	DASS-21(anxiety, stress, depression); MAAS (Dispositional Mindfulness)	Statistically significant improvement of the investigated outcomes	Journal Article
14	Alhawatmeh (2022) Jordan [39]	RCT	112 E: 56; C: 56 Dropout rate (3.57%) E: −2; C: −2	Meditation “of the sense” ABC Relaxation Theory protocol	Sitting quietly with closed eyes	MAAS (Dispositional Mindfulness); PSS (Stress); Biomedical Markers (Cortisol and C-Reactive Protein)	Serum Cortisol level and perceived stress have been significantly reduced. DM and CRP did not reach statistically significant level.	Journal article
15	Alsaraireh (2017) Jordan [40]	RCT	200 E: 100; C: 100 Dropout rate (9.5%) E: −9; C: −10	Mindfulness Meditation	Physical exercises	CESD-R(Depression)	Experimental group showed a significantly greater reduction in their depression score than the Control group.	Journal article
16	Burger (2017) Hawaii [41]	RCT	60 E: 32; C: 28 Dropout rate (13.33%) E: −4 C: −4	Mindfulness Meditation online and recording	Wait list	ANT (attention); PSS (Stress); FFMQ (dispositional mindfulness)	Statistically significant improvement of the investigated outcomes	Journal article
17	Can Gür (2020) Turkey [42]	RCT	132 E and C not specified Dropout rate −9 (7%) E: 61; C: 62	MBET	No intervention	JSENS (empathy); ADAS (Age discrimination)	Significant increase in the level of empathy in the Experimental group. No significant change in total age discrimination scores was recorded in either group	Journal article
18	Chase-Cantarini (2019) USA [43]	QL	64 Dropout rate Not specified	Mindfulness moments		Survey (stress, fatigue, engagement, focus, learning skills	Very popular with most students. A student did not like it; some felt sleepy. Some suggestions have been made for implementing mindfulness moments	Journal article
19	Cheli (2020) Italy [44]	RCT	82 E: 36; C: 46 Dropout rate (8.53%) E: −2; C: −5	MBEP (SANP)	Normal Educational Practice	CBI (Burnout); MAAS (Dispositional Mindfulness);	Statistically significant improvement of the investigated outcomes	Journal article
20	Colburn (2023) USA [45]	QL	69 Dropout rate Not specified	Mindful Self-Compassion Workshop with sandtray	-	Inquiry (students’ perception)	The participants appreciated the sandtray component, finding it useful for reflecting on strengths, life, emotions and experiences, and for expressing themselves.	Journal Article
21	Dai (2022) China [46]	RCT	120 E: 60; C: 60 Dropout rate (10%) E: −8, C: −4	MLWC	Health Education	DASS-21 (anxiety, stress, depression); FFMQ-SF ((dispositional mindfulness); PSSS (perceived social support)	Statistically insignificant reduction in depression. Statistically significant impact on anxiety, stress and awareness.	Journal Article
22	Ebrahem (2022) Egypt [47]	QE	78 Dropout rate −1 (1.28%)	Mindfulness Practices	-	AOCS (Obsessive-compulsive symptoms); SSI (Suicidal ideation); SES (Self-efficacy); MAAS (Dispositional Mindfulness)	The MBIs had a positive effect on improving self-efficacy and decreasing suicidal ideation and obsessive-compulsive symptoms.	Journal Article
23	ElKayal (2022) Egypt [48]	QE	160 8 clusters with 20 participants per cluster Dropout rate Not specified	Theoretical session and Mindfulness Practices	-	IES-R (Post-traumatic stress) FFMQ-15 (Dispositional Mindfulness)	PTS symptoms and DM improved significantly after the application of MBIs.	Journal Article
24	Franco (2022) USA [49]	QE	76 Dropout rate Not specified	TAO	-	DASS-21 (Depression, anxiety, stress); CSI-SF (Positive coping)	An improvement of the investigated outcomes	Journal Article
25	Hutchison (2016) UK [50]	QE	25 Dropout rate −5 (20%)	Mindfulness in Learning Disability Practice Workshop	-	A combination of open-ended questions and a Likert scale (Provocative behaviours; calm; dispositional mindfulness)	Positive effects of the workshop support the idea of embedding mindfulness training in the nursing curriculum.	Journal Article
26	Karaca (2019) Turkey [51]	RCT	114 E: 42; C: 72 Dropout rate (8.77%) E: −3; C: −7	MBSR	No intervention	Nursing Education Stress Scale (Academic stress); MAAS (Mindfulness skills); Stress Management Styles Scale (coping styles);	Statistically significant improvement on stress and awareness	Journal Article
27	Ksiksou (2022) Marocco [52]	QE	20 Dropout rate Not specified	MBSR		PSS-CP (Stress); EIS (Emotional intelligence)	An improvement of the investigated outcomes	Journal Article
28	Munif (2019) Indonesia [53]	QE	36 E: 18; C: 18	Islamic spiritual mindfulness	No intervention	DASS-42 (Stress)	An improvement of the investigated outcome	Journal Article
29	Öztürk (2023) Turkey [54]	RCT	64 E and C not specified Dropout rate −5 (8%) E: 29; C: 30	Adapted MBSR	No intervention	PSS (Stress); PWB (Psychological Well-Being); SEIS (emotional intelligence)	Statistically significant improvement of the investigated outcomes	Journal Article
30	Quatraro (2024) USA [55]	QE	35 Dropout rate −9 (25.71%)	Mindfulness exercise Pre-recorded audio files	-	PSS (Stress); SCS (Self-Compassion); BDI (Depression)	Statistically significant improvement on stress and depression levels. An improvement of self-compassion.	Journal Article
31	Ratanasiripong (2015) Thailand [56]	RCT	89 E1: 29 E2: 29 C: 29	E-1: Biofeedback E-2: Vipassana Meditation	No intervention	Improvement of academic performance; PSS (Stress); STAI (Anxiety)	Mindfulness Meditation improved anxiety and stress levels	Journal Article
32	Sari Ozturk (2022) Turkey [57]	RCT	180 E: 90; C: 90 Dropout rate (5,55%) E: −6; C: −4	Mindfulness-based mandalas	No intervention	STAI (Anxiety); SWBS (Spiritual well-being); SPANE (Emotional management)	Statistically significant improvement of the investigated outcomes	Journal Article
33	Scheick (2011) USA [58]	MM	30 E and C not specified Dropout rate −8 (27%) E: 15; C: 7	STEDFAST S-AM	-	Element S: Self-concept examination by Schutz (Dispositional mindfulness, self-control, reflective attention)	Statistically significant improvement of the investigated outcomes	Journal Article
34	Spadaro (2016) USA [59]	QE	27 Dropout rate −1(3.70%)	MBSR	-	PSS (Stress); HADS (Depression and anxiety); ANT (attention)	Statistically significant decrease in stress scores. Statistically insignificant decrease in stress scores. The ability to attention is improved.	Journal Article
35	Tarhan (2023) Turkey [60]	QE	78 E: 36; C: 42 Dropout rate Not specified	Short form MBSR	Traditional teaching on clinical errors	Attitudes Medical Error Scales (medical error); Student Satisfaction and Self-confidence Scale in Learning (Self-confidence); Simulation Design Scale (errors and risks in a simulation environment)	Statistically significant improvement for medical error attitudes. Statistically insignificant difference for self-confidence and satisfaction.	Journal Article
36	Torné-Ruiz (2023) Spain [61]	QE	52 E: 26 C: 26 Dropout rate (19.23%) E: −5; C: −5	10-daymindfulness	-	Self-administered Analogue Stress Scale (Stress); STAI (Anxiety); FFMQ (Awareness); Physiological Measures: (Blood Pressure)	Statistically significant improvement of the investigated outcomes	Journal Article
37	Yüksel (2020) Turkey [62]	QE	82 E: 41; C: 41 Dropout rate Not specified	MBCT	-	MAAS (Dispositional Mindfulness); DASS-21(anxiety, stress)	Statistically significant improvement of the investigated outcomes	Journal Article
38	Arthur (2022) USA [63]	RCT	57–60 Unclear	A record on Mindful Breathing	Relaxing music	Improvement of academic performance; MAAS (Dispositional Mindfulness); PSS (Stress)	No improvement in outcomes was observed	Doctoral dissertations
39	Baich (2022) USA [64]	QE	34 Dropout rate −9 (26%)	Mindfulness guide with smartphone	-	WTAS (anxiety)	Statistically significant impact on the reduction in anxiety score	Doctoral dissertations
40	Bonfe (2023) USA [65]	MM	24 Dropout rate Not specified	Short Mindfulness Meditation exercise online	-	QN: DASS-21(anxiety, stress, depression); QL: Inquiry (students’ perception)	QN: Statistically insignificant reduction in anxiety and stress QL: Increased sense of calm, relaxation, centering, focusing.	Doctoral dissertations
41	Davis (2020) USA [66]	RCT	120 12 clusters with 10 participants per cluster Dropout rate Not specified	Koru Mindfulness Meditation	Wait list	DASS-21 (stress; depression); ER (resilience); Brief COPE (coping strategies); ISEL-12 (personal resources and social support); SPANE (positive and negative emotions); PSS (burden of academic requirements); HPLP II (interpersonal relationships)	The MBIs was effective in lowering stress and depressive symptoms in nursing students.	Doctoral dissertations
42	Dodson (2021) USA [67]	QE	40 Dropout rate Not specified	MBSR: Burdick’s Workbook	-	TAI (anxiety; emotionality, worries)	Statistically significant improvement of the investigated outcomes.	Doctoral dissertations
43	Foster (2017) USA [68]	RCT	12 E: 6; C: 6	Theoretical session and Mindfulness Practices	Self-managed study with supervision	DSP (Stress, anxiety); LASSI (Learning and time management strategies)	An improvement of the investigated outcomes in Experimental group.	Doctoral dissertations
44	Heinrich (2022) USA [69]	RCT	195 E: 99; C: 96 Dropout rate (25.64%) E: −17; C: −33	Listening to 10 min of Meditation recording	Listening to 10 min recording on nursing notions	PSS (Stress) SCS^1^ (Self-compassion) MAAS (Dispositional Mindfulness); GAD-7 (Anxiety)	Statistically significant improvement of the investigated outcomes	Doctoral dissertations
45	Kinney (2022) USA [70]	QE	203 E: 111; C: 92 Dropout rate (59.60%) E: −59; C: −62	Mindful Breathing	No intervention	JSE-HPS (empathy)	No statistically significant difference	Doctoral dissertations
46	Klich (2019) USA [71]	QE	15 −6 (40%)	Mindfulness Meditation Book and the CD by Sharon Salzberg	-	IRI (Empathy); MAAS (Dispositional Mindfulness); STAI (Anxiety)	An improvement of the investigated outcomes	Doctoral dissertations
47	Leggett (2010) USA [72]	RCT	90 E and C not specified Dropout rate −5 (6%) E: 42; C: 43	Mindfulness Meditation	Usual treatment	CESD-R (Depression) MAAS (Dispositional Mindfulness); Student Clinical Completion Appraisal form (self-efficacy) Clinical Skills Evaluation (Clinical Skills performance) Physiological Measures: (Heart Rate; Blood Pressure)	Statistically significant decrease in depression scores and blood pressure. Statistically insignificant Improvements in mindfulness, self-efficacy, and clinical skills performance. No change for Heart Rate.	Doctoral dissertations
48	Lynch (2023) USA [73]	QE	122 Dropout rate −68 (56%)	Short Mindfulness Meditation exercise online	-	Survey (Stress, Resilience)	Students found the website easily accessible and useful. Students’ satisfaction is highlighted	Doctoral dissertations
49	Medari (2023) USA [74]	MM	102 Dropout rate −88 (86%)	MindShift^®^ CBT App from device		PSS (Stress); GSES (Self-efficacy)	QN: Not statistically significant differences. QL: Students suggest continuing to use it.	Doctoral dissertations
50	Oseguera (2019) USA [75]	MM	175 Dropout rate −100 (57%) QN: 60; QL: 15	Mindfulness Meditation	-	PSS (Stress); CAM-R (Dispositional mindfulness); Inquiry (Mindfulness experience)	QN: Statistically significant improvement on stress and awareness. QL: positive experience	Doctoral dissertations
51	Ross (2022) USA [76]	QE	79 Dropout rate −50 (63%)	Adapted MBSR	-	SOS-S and PSS (Stress) Survey (students’ perception)	No statistically significant difference Increased sense of calm and engaged	Doctoral dissertations
52	Simonton (2021) USA [77]	MM	57 Dropout rate −41 (72%)	Koru Mindfulness Meditation Online	-	STAI (Anxiety); Inquiry (Students’ experience; mindfulness strategies used)	QN data are scarce; QL: MBIs benefited the students in personal and professional life.	Doctoral dissertations
53	Tenrreiro (2022) USA [78]	QE	20 Dropout rate Not specified	MBSR	-	CTAS-2 (Anxiety)	An improvement of the investigated outcome	Doctoral dissertations
54	Teribury (2021) USA [79]	QE	26 Dropout rate −3 (12%)	Mindfulness Meditation	-	CTAS-2 (Anxiety)	An improvement of the investigated outcome but a negative linear relationship was observed with post-test CTAS-2 scores and mindfulness meditation performance for 200–1400 min.	Doctoral dissertations
55	Tung (2019) China [80]	RCT	88 E: 44 C: 44 Dropout rate (17.04%) E: −11; C: −4	Mindful Self Compassion (MSC) programme	Wait list	Chinese form: FFMQ (Awareness); SCS (Self-Compassion); ProQOL-5 (Compassion Satisfaction and Compassion Fatigue); PSS (Stress).	An improvement of the investigated outcome	Doctoral dissertations

Notes: Experimental group (E); Control group (C); Mindfulness Attention Awareness Scale (MAAS); Perceived Stress Scale (PSS); Dispositional Mindfulness (DM); Biomedical Markers: ELISA methods for Cortisol and CRP (C-Reactive Protein); Center for Epidemiologic Studies Depression Scale (CESD-R); Westside Test Anxiety Scale (WTAS); Mindfulness Based Stress Reduction (MBSR); The Interpersonal Reactivity Index (IRI); The Derogatis Stress Profile (DSP); Depression Anxiety and Stress Scale (DASS-21); 10-item Connor-Davidson Resilience Scale (CD-RISC-10); Attention Network Test (ANT); Five-Facet Mindfulness Questionnaire (FFMQ); Mindful Self-Care Scale (MSCS); Mindfulness-Based Empathy Training (MBET); Jefferson Empathy Scale (JSENS); Age Discrimination Attitude Scale (ADAS); Mindful Based Educational Program (MBEP); Self-Aware Nurse Project (SANP); Copenhagen Burnout Inventory (CBI); Self-Rating Anxiety Scale (SAS); Self-Rating Depression Scale (SDS); Mindfulness Living with Challenge (MLWC); Short Formed Five-Facets of Mindfulness Questionnaire (FFMQ-SF); Perceived Social Support Scale (PSSS); Ego Resilience Scale (ER); Interpersonal Support Evaluation List (ISEL-12); Scale of Positive and Negative Experience (SPANE); Health-Promoting Lifestyle Profile II (HPLP II); Test Anxiety Inventory (TAI); The Arabic Obsessive-Compulsive Scale (AOCS); Scale for Suicidal Ideation (SSI); Self-efficacy scale (SES); Event Impact Scale (IES-R); 15-item Five-Facet Awareness Questionnaire (FFMQ-15); Post-Traumatic Stress (PTS); Learning and Study Strategies Inventory (LASSI); Coping Strategies Inventory-Short Form (CSI-SF); Generalized Anxiety Disorder sub-scale (GAD-7); Self-Compassion Scale (SCS^1^); Psychosocial well-being index-short form (PWI-SF); State Trait Anxiety Inventory (STAI); Beck depression inventory (BDI); Jefferson Scale of Empathy for Health Professions Students JSE-HPS; Mindfulness-Based Cognitive Therapy (MBCT); Supporting Communication Scale (SCS^2^); Emotional Intelligence Scale (EIS); Caring Ability Inventory (CAI); Perceived Stress Scale in Clinical Practicum (PSS-CP); Peer-assisted Learning (PAL); Oldenburg Burnout Inventory—Student (OLBI-S); General Self-Efficacy Scale (GSES); Cognitive and Affective Mindfulness Scale-Revised (CAM-R); Perceived Stress Scale (PSS); Psychological Well-Being Scale (PWB); Revised Schutte Emotional Intelligence Scale (SEIS); Short Stress Overload Scale (SOS-S); Spiritual Well-Being Scale (SWBS); STEDFAST S-AM protocol: to improve awareness trough Self-Assessment, Therapeutic Role, Empathy, Detached Reflection, Facilitated Debriefing, Alert Empathy, Self-Awareness, Therapeutic Use of Self- Self Aware Mindfulness; Hospital Anxiety and Depression Scale (HADS); Cognitive Test Anxiety Scale (CTAS-2); Physio−Psycho−Social Response Scale (PPSRS).

**Table 3:** Intervention characteristics of mindfulness-based interventions (MBIs) reported in the reviewed article. First author, year of publication, country of study has been carried out, type of MBIs, its duration, modality of delivery, adverse events, the presence of facilitator/competence, and the context of intervention are also reported.

No.	First Author (Year)	MBIs	MBIs Duration	Mode of Delivery	Link Website/App	Adverse Events	Facilitator/Competence	Context
1	Niessen (2015) [19]	Mindfulness training	Four-week	In presence	-	Not specified	Not specified	1st Academic Year
2	Beddoe (2004) [27]	MBSR in presence and audio guide recorded at home	eight weeks with one session per week of two hours in presence and a commitment of 30 min at home (listening to audio recording)	In presence with Supplementary Materials for home practice	-	Not specified	Not specified	Ad hoc workshop and home with recorded audio tracks
3	Bultas (2021) [28]	Short Mindfulness Meditation exercise online	Twenty minutes before the exam for 5 exams	Online	https://www.youtube.com/watch?v=3yxgFAW7wTc (accessed on 22 November 2025)	No adverse events reported	The authors did not have skills as facilitators and for this reason they used YouTube. Not specified the skills of the video’s author.	Online before exams
4	Burner (2023) [29]	Mindfulness Meditation and MBSR	Sessions took place weekly or every other week, depending on the weeks in which the skills workshops were scheduled. The sessions lasted from 10 to 30 min depending on the time available in the skills lab programme	In presence with Supplementary Materials for home practice	-	No adverse events reported	The first female researcher. Self-paced online courses. MBSR practitioner. Specialized in Mental Health Nursing	Before the nursing skills workshop session
5	Chiam (2020) [30]	Mind-Nurse Program	Eight sessions of one hour and half and 10–20 min practice at home	In presence	-	“I have not received any reports of side effects. Some students felt asleep during the mindfulness practice during the session.” (Au)	Last author of the article. Mindfulness facilitator experiences. Specialization in Mental Health Nursing. Formal training (with certificate) on the Mindfulness-Based Stress Reduction (MBSR) programme in Singapore. Continuing informal training (monthly training) from a meditation practitioner in Thailand.	Ad hoc workshop and home practice
6	Kou (2022) [31]	MBSR MBCT	Two hours per session, one session per week for eight weeks	In presence with Supplementary Materials for home practice	-	No adverse events reported	The Researcher. Experienced mindfulness facilitator (>5 years) who has incorporated the principles of the Unified Mindfulness system into the training	Extracurricular course in presence and at home
7	Lam (2024) [32]	Mindfulness-based PAL	Five-forty minutes for eight weeks; (from nine to 16 weeks)	Online workshops and audio guides at home	-	No adverse events reported	Research Assistant and Peer Leader. The Research Assistant Trainer is a qualified mindfulness instructor who has extensive experience in providing mindfulness training to healthcare professionals.	Extracurricular peer to peer online course
8	Pollard (2020) [33]	Mindfulness moment before clinical simulation	Two minutes before the three-hour, five-match simulation	In presence	-	No adverse events reported	Course Facilitators. Script preparations and facilitator training by a mindfulness expert, Dr. Shelley Winton of Alberta Health Services	Before participating in the gestural simulation workshops
9	Schwarze (2015) [34]	MBCT Recorded guide on Podcast	Six one-hour sessions	In presence with Supplementary Materials for home practice	Maddux and Maddux podcast (2006) https://itunes.apple.com/us/podcast/meditation-oasis/id204570355?mt=2 (accessed on 22 November 2025)	No adverse events reported	Podcast authors are Mindfulness Meditation experts. Licensed Professional Counselor Trainer	Spring Semester
10	Uysal (2022) [35]	MBSR	Two hours two days a week for four weeks	In presence with Supplementary Materials for home practice	-	“One student said that his mindfulness practice had increased his awareness, but that his stress level had also increased. He described himself as a person with a lot of anxiety and stress. However, he remained in the intervention group” (Au)	Researcher. Trained in the MBSR	Extra-Curricular Course
11	Chen (2013) [36]	Mindful breathing	30 min a day for seven consecutive days	In presence	-	Not specified	Senior Consultant Psychologist, Expert in the practice of Mindfulness meditation techniques	Ad hoc workshop
12	Kang (2019) [37]	Stress Coping Program MBIs	Ninety minutes per session for eight weeks (one session per week)	In presence	-	Not specified	The researcher. Professional training in mindfulness meditation with eight years of conducting experience.	Ad hoc workshop during the internship
13	Song (2015) [38]	MBSR	Two hours a week for eight weeks	In presence with Supplementary Materials for home practice	-	Not specified	Mindfulness Instructor. Over 10 years of experience in MSR	Extra-Curricular Course
14	Alhawatmeh (2022) [39]	Meditation “of the sense” ABC Relaxation Theory protocol	Three hours of plenary to explain the study. Five weekly sessions of 30 min of mindfulness meditation	In presence	-	Not specified	Not specified	Ad hoc workshop
15	Alsaraireh (2017) [40]	Mindfulness Meditation	1 h for 3 days a week for 10 weeks	In presence	-	Not specified	Not specified	Ad hoc workshop
16	Burger (2017) [41]	Mindfulness Meditation online and recording	Ten minutes a day for 4 weeks	Online University of Wisconsin Podcast-Department of Public Health	https://www.fammed.wisc.edu/mindfulness-meditation-podcast-series/ (accessed on 30 October 2024)	Not specified	Senior Researcher, that trained as a mindfulness facilitator and daily practice	Online at home in the first half of the year
17	Can Gür (2020) [42]	MBET	Eight weeks: one hour twice a week for a total of 16 meetings	In presence with Supplementary Materials for home practice	-	Not specified	Significant increase in the level of empathy in the Experimental group. No significant change in total age discrimination scores was recorded in either group	Ad hoc workshop
18	Chase-Cantarini (2019) [43]	Mindfulness moments	Five-ten minutes before the theory lesson every week of a semester	in presence before the lesson		Not specified	The authors: nurse educators without specific training	Before the theoretical lessons
19	Cheli (2020) [44]	MBEP (SANP)	Six-weeks MBEP: Five regular sessions of three hours and one session of four hour and half	In presence	-	Not specified	Specific training and supervision, at least 2 years of experience as a Mindfulness teacher	Replacement of a pre-existing course in General Pedagogy. MBIs has been delivered in conjunction with the final internship
20	Colburn (2023) [45]	Mindful Self-Compassion Workshop with sandtray	Four-hour workshop	In presence	-	Not specified	The researcher. A Certificate of Advanced Practice in Mind–Body Medicine	Workshop part of the curriculum of nursing students
21	Dai (2022) [46]	MLWC	Variable for intervention; 30–40 min per lesson (two lessons per session) for six weeks	Online	-	Not specified	Two psychiatrists. Certified Mindfulness Facilitator at Mindfulness Awareness Research Center California	Extracurricular course
22	Ebrahem (2022) [47]	Mindfulness Practices	Not specified	Not specified	-	Not specified	Not specified	Not specified
23	ElKayal (2022) [48]	Theoretical session and Mindfulness Practices	Forty-five/sixty minutes per session for a total of eight sessions in three months	In presence	-	Not specified	Researchers. Certificated in mindfulness-based stress reduction techniques (MBSR) at the Zagazig University Psychiatric Center.	Ad hoc workshop
24	Franco (2022) [49]	TAO Bookstore: Mindfulness Practices	Twelve mindfulness exercises lasting from two to eleven minutes for a total of four weeks	Online	https://www.taoconnect.org/ (accessed on 22 November 2025)	Not specified	Course Facilitator (Interactive Site). Therapy Assistance Online (TAO) is a peer-reviewed interactive website developed by Dr. Susan Benton in 2012	During a semester
25	Hutchison (2016) [50]	Mindfulness in Learning Disability Practice Workshop	Nine hours in two days	In presence	-	“College nursing programs need to consider how the application of mindfulness can create emotional dependency in students.” (Au)	Psychologist. Lecturer trained at a seminar on leadership development Decide, Commit, Proceed.	Ad hoc workshop
26	Karaca (2019) [51]	MBSR	Ninety/Ninety-five minutes twice a week for a total of 12 weeks	In presence with a request for 10 min a day at home	-	Not specified	Researcher, that was a cognitive and behavioural therapist. European Association for Behavioural and Cognitive Therapies Accreditation Certificate.	MBSR has been included in the Coping course programme during the autumn semester
27	Ksiksou (2022) [52]	MBSR	Sessions of 2.30 h per week with a minimum of 20 min of home practice for a total of eight weeks	In presence with Supplementary Materials for home practice		Not specified	Psychiatrist Expert in MBSR. MBSR Specialization	Extracurricular course in presence and at home
28	Munif (2019) [53]	Islamic spiritual mindfulness	Twenty-five minutes for five days at home: morning, afternoon and evening.	In presence with Supplementary Materials for home practice	-	Not specified	Not specified	Not specified
29	Öztürk (2023) [54]	Adapted MBSR	Ninety minutes twice a week. Four-hour retreat and not eight hours.	Online	-	Not specified	The researcher. Certified Cognitive-Behavioural Therapist, Mindfulness and Acceptance Engagement Professional	Extra-Curricular Course
30	Quatraro (2024) [55]	Mindfulness exercise Pre-recorded audio files	Six weeks for three/four days a week for five/seven minutes	Online through mobile App Smiling Mind	https://www.smilingmind.com.au (accessed on 22 November 2025)	Not specified	Not specified	3rd and 4th year students
31	Ratanasiripong (2015) [56]	Vipassana Meditation	Three times a day for four weeks. Not specified time for each session	In presence	-	Not specified	Meditation instructor. Not specified certificate	Before the clinical internship
32	Sari Ozturk (2022) [57]	Mindfulness-based mandalas	Three one-hour meetings for three weeks, one meeting per week.	Online	-	Not specified	Researcher. Mindfulness-based mandala drawing certificate and a therapeutic art life coach certificate	During the clinical internship in the COVID period
33	Scheick (2011) [58]	STEDFAST S-AM	Three minutes of breathing before the meetings. Not specified the number of meetings	In presence	-	Not specified	Not specified	Two semesters for community psychiatric and mental health nursing
34	Spadaro (2016) [59]	MBSR	One day a week for eight weeks. Not specified the time for each session	Online	-	Not specified	Not specified	During a semester (not the last)
35	Tarhan (2023) [60]	Short form MBSR	One hour per week for four weeks	In presence	-	Not specified	Second author. Trained in MBSR	Ad hoc workshop
36	Torné-Ruiz (2023) [61]	10-daymindfulness	Twenty-five minutes in 10 days	Online	https://www.youtube.com/playlist?list=PLokeFpXsus96XL_E2rgQTnd9YxFPlwzix (accessed on 22 November 2025) https://www.cc.nih.gov/sites/default/files/internet-files/palliativecare/pdf/MindfulnessManual.pdf (accessed on 30 October 2024)	Not specified	Mindfulness instructor. Awareness expert with experience in conducting courses and training	Before the clinical simulations
37	Yüksel (2020) [62]	MBCT	Two hours a week for eight weeks	In presence	-	Not specified	The first researcher. Training and supervision on cognitive and behavioural therapy and mindfulness-based stress reduction	Extra-Curricular Course
38	Arthur (2022) [63]	A record on Mindful Breathing	3 min before theory classes until exam day	A recording on Mindful Breathing listened in presence	-	Not specified	Researcher unrelated to the degree course. Competence not specified	before the theoretical lessons
39	Baich (2022) [64]	Mindfulness guide with smartphone	12 days in 5 weeks (for a total of 75 min with an average per session of 6.32 min)	Online through mobile App “Smiling mind”	https://www.smilingmind.com.au (accessed on 22 November 2025)	Not specified	Not specified	Online, before theoretical lessons
40	Bonfe (2023) [65]	Short Mindfulness Meditation exercise online	five minutes before the exam for six exams	Online before the exam through the semester	https://www.youtube.com/watch?v=3yxgFAW7wTc (accessed on 22 November 2025)	Not specified	The researcher who implemented the project. He completed a 20 min basic training in mindfulness. The training included motivation and guide to exercise of awareness.	Introduced during the pharmacology course. Before the exams of the whole semester
41	Davis (2020) [66]	Koru Mindfulness Meditation	Seventy-five minutes per session; one session per week for four weeks	Online	-	Not specified	Psychiatrist and Koru Mindfulness Meditation instructor. Expert Certificate	Extra-Curricular Course
42	Dodson (2021) [67]	MBSR: Burdick’s Workbook	Every week in class before class for 15–20 min before the placement test	In presence	-	Not specified	Expert-trained teachers. Three weeks in the first half according to the Burdick protocol (2013)	Before the classroom lesson until the final test (1st semester)
43	Foster (2017) [68]	Theoretical session and Mindfulness Practices	one hour session for eight sessions	In presence with Supplementary Materials for home practice	-	Not specified	Mindfulness Facilitator. Formal training in mindfulness-based stress reduction (MBSR) from the Oasis Institute (University of Massachusetts, 2014), which is part of the Center for Mindfulness in Medicine, Healthcare, and Society (CMMHCS)	Ad hoc workshop before the internship experience
44	Heinrich (2022) [69]	Listening to 10 min of Meditation recording	Ten minutes of Meditation recording three days a week for four weeks	Audio resources	-	Not specified	Recorded Meditation. Not specified author’s competences.	Not specified
45	Kinney (2022) [70]	Mindful Breathing	One minute and 30 s	Online	Dr. Andrew Weil, (breathing 4-7-8) https://www.youtube.com/watch?v=YRPh_GaiL8s (accessed on 22 November 2025)	Not specified	The host of the video is Dr. Andrew Weil. Dr. Andrew Weil is a holistic physician	Before and after the theoretical lessons
46	Klich (2019) [71]	Mindfulness Meditation Book and the CD by Sharon Salzberg	Fifteen minutes a day of meditation and 30 min a week of reading for four weeks	At home with Supplementary Materials for practice (Book and the CD by Sharon Salzberg)	-	“No adverse events occurred. Recruits were briefed on potential risks and provided with contact information for mental health services as a precaution.” (Au)	Book and cd by Sharon Salzberg’s Real Happiness: The Power of Meditation: A 28-Day Program book and audio CD. Sharon Salzberg is a meditation teacher	Extracurricular at home
47	Leggett (2010) [72]	Mindfulness Meditation	A session of one hour and half per week for three weeks	In presence with daily practice of 20 min at home	-	Not specified	Experienced Mindful Breathing Facilitator	Ad hoc workshops
48	Lynch (2023) [73]	Short Mindfulness Meditation exercise online	Not specified	Online	-	Not specified	Not specified	Not specified
49	Medari (2023) [74]	MindShift^®^ CBT App from device	Ten minutes five times a week for six weeks	Online through mobile App MindShift^®^ CBT	https://www.youtube.com/embed/0Ka10cf9dSY?feature=oembed (accessed on 30 October 2024)	Not specified	Not specified	Academic Semester
50	Oseguera (2019) [75]	Mindfulness Meditation	Five minutes a day for four weeks	In presence with online video	-	Not specified	The teachers. Not specified competences.	In the first five minutes of the theory lesson
51	Ross (2022) [76]	Adapted MBSR	Fifteen minutes sessions before the start of the theoretical course (7:35 am to 7:50 am; lesson start time 8:00 am) one session per week	In presence with daily practice of 10–30 min at home	-	Not specified	The first researcher under the supervision of the course teacher who had an interest in Mindfulness Meditation	During the first semester before the begin of the course
52	Simonton (2021) [77]	Koru Mindfulness Meditation Online	Five-ten minutes of MK per day for five weeks	Online	-	Not specified	Not specified	Before High-Fidelity Simulations
53	Tenrreiro (2022) [78]	MBSR	Not specified	Not specified	-	Not specified	Not specified	Not specified
54	Teribury (2021) [79]	Mindfulness Meditation	Eight weeks. Not specified the time of each connection to the App	Online through mobile App Headspace	https://www.headspace.com/headspace-meditation-app (accessed on 22 November 2025)	Not specified	Not specified	During a semester
55	Tung (2019) [80]	Mindful Self Compassion (MSC) programme	Eight weeks with one session per week lasting three hours and a half-day retreat scheduled between the fourth and fifth sessions	In presence	-	Not specified	MSC Certified Teacher	Not specified

## Data Availability

No new data were created or analyzed in this study. Data sharing is not applicable to this article.

## Supplementary Materials

The following supporting information can be downloaded at https://www.mdpi.com/article/10.3390/healthcare14010130/s1: Supplementary File S1: PRISMA checklist. Supplementary File S2: Data Extraction Table ScR.

## Abbreviations

The following abbreviations are used in this manuscript:

MBIs	Mindfulness-Based Intervention
DM	Dispositional Mindfulness
MSC	Mindful Self Compassion
ScR	Scoping Review
MBSR	Mindfulness-Based Stress Reduction
MBCT	Mindfulness-Based Cognitive Therapy
JBI	Joanna Briggs Institute

## Appendix A

### Search Strategy

**Database**	**Search String**
Pubmed	((“nursing student*”[All Fields] OR “baccalaureate”[All Fields] OR “baccalaureates”[All Fields] OR “undergraduate nursing student”[All Fields] OR “pre-licensure”[All Fields]) AND (“mind s”[All Fields] OR “minded”[All Fields] OR “mindful”[All Fields] OR “mindfulness”[MeSH Terms] OR “mindfulness”[All Fields] OR “minding”[All Fields] OR “minds”[All Fields] OR (“self compassion”[MeSH Terms] OR “self compassion”[All Fields] OR (“self”[All Fields] AND “compassion”[All Fields]) OR “self compassion”[All Fields])) AND (“self care”[MeSH Terms] OR (“self”[All Fields] AND “care”[All Fields]) OR “self care”[All Fields] OR (“psychologic”[All Fields] OR “psychological”[All Fields] OR “psychologically”[All Fields] OR “psychologization”[All Fields] OR “psychologized”[All Fields] OR “psychologizing”[All Fields]) OR “well*”[All Fields] OR (“psychological well being”[MeSH Terms] OR (“psychological”[All Fields] AND “well being”[All Fields]) OR “psychological well being”[All Fields] OR (“psychological”[All Fields] AND “well”[All Fields]) OR “psychological well being”[All Fields]) OR (“self efficacy”[MeSH Terms] OR (“self”[All Fields] AND “efficacy”[All Fields]) OR “self efficacy”[All Fields]) OR (“stress”[All Fields] OR “stressed”[All Fields] OR “stresses”[All Fields] OR “stressful”[All Fields] OR “stressfulness”[All Fields] OR “stressing”[All Fields]) OR (“anxiety”[MeSH Terms] OR “anxiety”[All Fields] OR “anxieties”[All Fields] OR “anxiety s”[All Fields]) OR “emotional intelligence”[All Fields] OR (“empathy”[MeSH Terms] OR “empathy”[All Fields]) OR (“resilience, psychological”[MeSH Terms] OR (“resilience”[All Fields] AND “psychological”[All Fields]) OR “psychological resilience”[All Fields] OR “resilience”[All Fields] OR “resiliences”[All Fields] OR “resiliencies”[All Fields] OR “satisfaction compassion”[All Fields] OR (“fatiguability”[All Fields] OR “fatiguable”[All Fields] OR “fatigue”[MeSH Terms] OR “fatigue”[All Fields] OR “fatigued”[All Fields] OR “fatigues”[All Fields] OR “fatiguing”[All Fields] OR “fatigueability”[All Fields]) OR “resiliency”[All Fields] OR “resilient”[All Fields] OR “resilients”[All Fields])) AND (“hasabstract”[All Fields] AND “loattrfull text”[Filter]) AND (“hasabstract”[All Fields] AND “loattrfull text”[Filter])) AND ((fha[Filter]) AND (fft[Filter]))
	
**Database**	**Search String**
Cinahl	(nursing students or student nurses or undergraduate student nurses or pre-licensure nurse) AND (mindfulness based stress reduction or mindfulness or mbsr or mindfulness intervention or mindfulness-based interventions or mindful self-compassion or self compassion or self-compassion or MSC) AND (wellbeing or well-being or well being or quality of life or wellness or health or positive affect or mental health or psychological well-being or self-care) Limits: Abstract; full text
PsycInfo	(nursing students or student nurses or undergraduate student nurses or pre-registration nursing student) AND (mindfulness based stress reduction or mindfulness or mbsr or mindfulness intervention) OR (mindful self compassion or mindful self-compassion) AND (wellbeing or well-being or well being or quality of life or wellness or health or positive affect or mental health) AND (trial or study or experiment or treatment or intervention) Limits: link full-text
ProQuest	AB,TI(“nursing students”) AND (“mindfulness-based interventions” OR “mindful self-compassion”) AND (experimental study OR quasi-experimental study) Limit: full-text
ERIC	“nursing students” AND self-compassion
	“nursing students” AND mindfulness
	
**Other retrivies**	**Search string**
Google Scholar	“undergraduate nursing students” AND (“mindfulness-based interventions” OR “mindful self-compassion”) AND well-being Limits: 2020
