# Supplementary material for: Mindfulness-Based Interventions to Implement the Psychological Well-Being of Nursing Students: A Scoping Review
Source: Healthcare (Basel). 2026 Jan 4;14(1):130. doi: 10.3390/healthcare14010130 (PMC12786117; doi:10.3390/healthcare14010130)
Supplement: Supplementary file 1 [file healthcare-14-00130-s001.zip › Suppl Files/Supplementary file 2_ DATA EXTRACTION TABLE ScR.pdf]

Mindfulness-Based Interventions to Implement the Psychological Well-Being of Nursing Students: A Scoping Review- Data Extraction Table

Au: Milena Consorte, Elena Morotti, Fabio Nanni, Alessandro Giannandrea, Stefano Benini and Monica Martoni

**Legends:**	
**MBIs**	**Mindfulness-based interventions**
**DM**	**Dispositional Mindfulness**
**(D/T)**	**Dissertations/Theses**
**E-C**	**Experimental-Control**
**Mm**	**Mindfulness Meditation**
**MM**	**Mixed Method**
**NP**	**Not possible (no email reference)**
**NS**	**Not specified**
**QE**	**Quasi-experimental study**
**QL**	**Qualitative study**
**QN**	**Quantitative study**
**RCT**	**Randomized Controlled Trial**

**Reference** [19]	Niessen & Jacob, 2015
**Country**	Oland
**Study Design**	MM (Pilot Cohort Study)
**Sample Size**<br><br>**Dropout score**	201 nursing students who attended the 1st academic year -Nursing Degree<br>(E: 123; C: 63)<br>*Dropout rate*<br>E: 95 (23%); C:70 (10%)
**MBIs applied**	NS
**MBIs duration**	The intervention consisted of the four-week mindfulness training and the pilot included an intervention group and a waiting list control group
**Mode of Delivery**	NS
**Outcome(s)**<br><br>**Variable(s)**	QN part:<br><br>What are the effects of a four-week mindfulness intervention on self-reported mindfulness and (self-) compassion in first-year nursing students?<br><br>QL part:<br><br>What are the experiences of students and trainers on the course with the four-week mindfulness intervention as part of the wider goals of the person-centred nursing module
**Outcome(s) Measures**	QN part:<br><br>- Five Facet Mindfulness Questionnaire Short-Form<br>- Self-Compassion Scale Short-Form<br>QL part:<br><br>focus group
**Any adverse events related to MBIs interventions**	NN
**Facilitator(s)**	NS
**Facilitator's competencies**	NS

**Context (e.g.: course year, theoretical lessons; internship)**	1st academic year<br><br>Mindfulness practices were introduced into the nursing curriculum as part of a person-centred nursing project
**Finding**	QN part:<br><br>no statistically relevant effects could be reported between the intervention group and the control group<br><br>QL part:<br><br>For most of the students, it was a useful experience both for their personal lives and for their professional future, helping them to understand what they feel and that it is right to dedicate time to their own care
**Limits**	Small size sample
**If unclear content, the author of the correspondence has been contacted**	NP
**He replied within the indicated time (one week)**	
**Reference** [27]	Beddoe & Murphy 2004
**Country**	USA
**Study Design**	MM
**Sample Size**<br><br>**Dropout score**	23 nursing students.<br><br>Dropout rate: -7 (30%)
**MBIs applied**	MBSR in presence and audio guide recorded with diary of the practice at home.<br>Practices:<br>* The body scan, a progressive relaxation in which participants direct attention and observe sensations.<br><br>* Seated meditation, which involves observing one's breathing, sensations, emotions, sounds, and thoughts.<br><br>* Hatha yoga, which involves gentle physical poses integrated with breathing to develop strength, flexibility and balance.<br><br>* Walking meditation, which involved slow, careful walking.<br><br>The course also explored the use of mindfulness in daily life, the psychological and physiological effects of stress, and journaling.

**MBIs duration**	eight weeks with one session per week of two hours in presence and a commitment of 30 minutes at home (listening to audio recording)
**Mode of Delivery**	In-person and at home
**Outcome(s)**<br><br>**Variable(s)**	- Stress reduction<br>- Increased Empathy Levels<br>- Behavior change<br>- emotional intelligence<br>- Investigating student perception
**Outcome(s) Measures**	- The Interpersonal Reactivity Index (IRI) for empathy<br>- The Derogatis Stress Profile (DSP) for stress<br>- Homework questionnaire<br>The questions required analogue, Likert and narrative answers. Using an 11-point Likert scale, participants were asked to rate the benefits of mindfulness techniques on a scale from 0 (practice was futile) to 10 (practice was very helpful).<br>Another section asked students to quantify the amount of change they experienced in various attitudes and behaviors as a result of the course.<br>During the course, homework was also entered in the diary and a log was provided to keep track of the tasks.
**Any adverse events related to MBIs interventions**	NS
**Facilitator(s)**	NS
**Facilitator's competencies**	NS
**Context (e.g.: course year, theoretical lessons; internship)**	Ad hoc and home workshop with recorded audio tracks
**Finding**	Significantly reduced student anxiety (p>.05). Favorable trends were observed in a number of dimensions of stress, including attitude, time pressure, and total stress.<br>Two dimensions of empathy, personal discomfort and fantasy, have also shown favorable downward trends.<br>Regular home meditation was related to additional benefits.<br>Participants reported using meditation in their daily lives and experiencing increased well-being and better coping skills as a result of the program.<br>The findings suggest that being mindful can also reduce the tendency to deal with the negative emotions of others. Coping with stress and promoting affective dominance are important aspects of nursing education that can be facilitated by training.
**Limits**	The results of this study are limited by a small, self-selected sample, the absence of a control group, a single location, a 22% path dropout rate, and another 8% with no post-test pre-test matches.<br>Students who volunteered may have perceived that they were suffering from stress or were naturally attracted to meditation and yoga and, therefore, were more motivated than individuals included in a randomized sample. Other limitations include the fact that the content of the intervention emphasized self-care but did not directly encourage empathy

	for others. Despite these limitations, the results indicate that nursing students' anxiety was reduced through mindfulness practice. Although the data on empathy did not show statistically significant changes, the trends were positive in the expected direction regarding the dimensions of personal distress and fantasy. These results provide strong support for a more comprehensive study that includes a randomized control group and a larger sample. It is recommended that the MBSR be adapted for nursing students so that the course integrates the cognitive component of empathy with poetry, discussion, and specific meditations on compassion, and that the course includes more yoga intervention, a frequent request from students.
**If unclear content, the author of the correspondence has been contacted**	April 2, 2024
**He replied within the indicated time (one week)**	April 2, 2024. He had not kept the study documentation (for the past time)
**Reference** [28]	Bultas et al., 2021
**Country**	USA
**Study Design**	MM
**Sample Size**<br><br>**Dropout score**	48 students who attended the 2nd academic year - Nursing Degree<br>(E: 24-C: 24)<br>*Dropout rate:* (18,75%) E: -9 C: 0
**MBIs applied**	E: short pre-examination mindfulness meditation exercise on YouTube (Executive Mind, 2016)<br>C: usual care
**MBIs duration**	20 minutes before the exam for 5 exams
**Mode of Delivery**	A Mindfulness Meditation exercise (breathing and attention) on YouTube<br>https://www.youtube.com/watch?v=3yxgFAW7wTc
**Outcome(s)**<br><br>**Variable(s)**	- Reduced anxiety<br>- Increased DM levels<br>- Increased levels of resilience<br>- Student perception
**Outcome(s) Measures**	Mindfulness Attention Awareness Scale (MAAS);<br>Perceived Stress Scale (PSS)<br>10-item Connor-Davidson Resilience Scale (CD-RISC-10)
**Any adverse events related to MBIs interventions**	No adverse events reported
**Facilitator(s)**	The authors administered the intervention on YouTube
**Facilitator's competencies**	The authors did not have skills as facilitators and for this reason they used YouTube.<br>NS the skills of the author of the video.
**Context (e.g.: course year, theoretical lessons; internship)**	Online before exams

**Finding**	QN: Results are not statistically significant but there is a tendency to reduce the level of anxiety, to increase the level of resilience. The changes to the MAAS are not significant;<br>QL: greater sense of calm, confidence, awareness
**Limits**	The small sample size was a limitation in this study as it is difficult to ascertain any true benefit or harm from the intervention without adequate potency, and generalization of results is limited. In addition, the researchers were aware that practicing true mindfulness requires regular practice over a period of time for participants to become competent. However, the objective of this study was to assess whether any effect could be ascertained through the use of a brief intervention that any untrained student could adopt before taking the exams.
**If unclear content, the author of the correspondence has been contacted**	May 23, 2024
**He replied within the indicated time (one week)**	May 23, 2024.<br>The following sections have been integrated: Any adverse events; Who administered the surgery; The skills of the facilitator.
**Reference** [29]	Burner & Spadaro, 2023
**Country**	USA
**Study Design**	QE
**Sample Size**<br><br>**Dropout score**	67 nursing students<br>*Dropout rate:* -9 (13%)
**MBIs applied**	MBIs:<br>- Raisin meditation<br>- Mindful breathing<br>- Body Scan<br>- Mm Informal practice (hand washing)<br>*Description of the intervention:*<br>The facilitator (S. C.) began session one with an overview of mindfulness concepts and with a mindful eating practice.<br>For the second session, S. C. discussed breath awareness and conducted a 5-minute guided exercise in class.<br>In the third session, S. C. He discussed body scanning which involves sequentially paying attention from the toes upwards to the head noticing sensations (heat, cold, pressure, or lack of sensation) in different parts of the body.<br>The third session included a 3-minute sit-down body scanning exercise guided by the S. C.<br>During session four, S. C. had the students visualize conscious hand washing (paying attention to sounds, sensations of soap and water, visualizing away stress, negative thoughts and/or tension).<br>The week following session four, participants again completed the MSCS and PSS and provided qualitative and open comments on a feedback form. During the study, S. C. uploaded that week's mindfulness content to a "Stress Reduction" folder in the Online Learning Management System (LMS) and posted reminders in the News Forum. S. C. also sent about one email per week to participants, offering additional awareness resources in the form of websites, videos, or articles. Four weeks after completing the sessions, S. C. held a follow-up session with all students about their experiences using mindfulness techniques and to get feedback on the sessions. Participants once again completed the PSS and MSCS.

**MBIs duration**	Sessions took place weekly or every other week, depending on the weeks in which the skills workshops were scheduled. The sessions lasted from 10 to 30 minutes depending on the time available in the skills lab program. Overall time of four weeks with four-week follow-up.
**Mode of Delivery**	In presence with supplementary materials for home practice
**Outcome(s)**<br><br>**Variable(s)**	- Stress reduction<br>- Self-care skills training
**Outcome(s) Measures**	Perceived Stress Scale (PSS)<br>Mindful Self-Care Scale (MSCS)
**Any adverse events related to MBIs interventions**	No adverse events reported
**Facilitator(s)**	The first female researcher
**Facilitator's competencies**	Self-paced online courses. MBSR practitioner. Specialized in Mental Health Nursing
**Context (e.g.: course year, theoretical lessons; internship)**	Before the nursing skills workshop session
**Finding**	There was no significant change in PSS scores from pre to post (p = .223; 95% confidence interval [CI] -3.6%-15.0%). At four-week follow-up, mean PSS scores increased by 3.9% (p = .067; CI = -0.4%-15.3%) from post-intervention scores, although again not significantly perhaps for academic load of subsequent semesters. significant differences in MSCS scores between time points (p <.0001). To assess which time points differed significantly from each other in this ANOVA, pairwise tests were conducted. MSCS scores did not change significantly from pre-intervention to post-intervention for participants as a whole (p = .605; 95% CI = -2.8%-4.7%). Students (n = 14) who practiced one or more mindfulness techniques (mindful eating, breath awareness, body scanning, and/or mindful handwashing) more than 8 times outside of the classroom were considered "engaged" with mindfulness intervention. Those who practiced eight or fewer times outside of the classroom were considered "less engaged." For engaged students, post-intervention MSCS scores increased by 9.5% (p = 0.01, 95% CI 2.7%-16.2%), while for less engaged students there was no difference (p = 0.197). However, increases in MSCS score were not sustained at four weeks follow-up, when scores decreased significantly from post-intervention scores for both engaged participants (p = 0.0006) and less engaged participants (p = 0.0004) four-week follow-up, mean MSCS scores decreased by 8.8% (p <,001; CI = -12.0%-5.6%) compared to post-intervention scores.<br>The study was shown to be feasible and acceptable to students (86.6% retention) and appeared effective in increasing self-care when put into practice.
**Limits**	One limitation was that mindfulness sessions were often timed prior to skills lab check-offs due to the nursing instructor's request, and students reported difficulty focusing on mindfulness content under those circumstances. Another limitation was the brevity of the individual sessions and the general intervention which may not have allowed the full

	assimilation of the mindfulness training given the inherent pressure of the clinical skills course in general. Finally, nursing students may not have taken this mindfulness skill training as seriously as learning the other nursing skills. Students should understand that nursing takes a toll on their physical and mental health and that mindfulness improves nursing outcomes and holistic self-care through stress management, and personal health.
**If unclear content, the author of the correspondence has been contacted**	May 23, 2024
**He replied within the indicated time (one week)**	May 24, 2024<br><br>The following sections have been integrated: Any adverse events; who administered intervention; The skills of the facilitator
**Reference** [30]	Chiam et al., 2020
**Country**	China
**Study Design**	QL
**Sample Size**<br><br>**Dropout score**	32 nursing students<br><br>Dropout rate<br><br>-12 (37,5%)
**MBIs applied**	MIND-NURSE program.<br>Each session included a didactic component and a guided mindfulness practice.<br>In addition to the face-to-face sessions, daily practice at home was required.<br>In addition, participants were given an educational booklet containing theoretical and practical information and a logbook to record home practice.<br>Theoretical part:<br>- Conscious stress management<br>- Body and sensations<br>- Awareness of emotions<br>- Conscious Thought Management<br>- Interpersonal awareness<br>- Loving-kindness and compassion<br>- Integrating mindfulness into daily activities<br>- Established practice of mindfulness<br><br>Experiential part:<br>- Mindful breathing<br>- Body Scan<br>- Letting go of thoughts<br>- Loving-kindness meditation
**MBIs duration**	8 sessions of 1.30 hours and 10-20 minutes practice at home
**Mode of Delivery**	In presence
**Outcome(s)**<br><br>**Variable(s)**	Exploring students' perceptions of the MBP program

**Outcome(s) Measures**	focus groups. The themes that emerged were: a) greater awareness of the present moment; b) greater serenity through the practice of mindfulness; c) triad of thoughts, emotions and behaviors; d) work towards interpersonal awareness; e) adapt awareness to nursing practice; and f) overcoming challenges for the practice of mindfulness
**Any adverse events related to MBIs interventions**	"I have not received any reports of side effects. Some students felt asleep during the mindfulness practice during the session."
**Facilitator(s)**	Last author of the article
**Facilitator's competencies**	Mindfulness facilitator experiences.<br>Specialization in Mental Health Nursing. Formal training (with certificate) on the Mindfulness-Based Stress Reduction (MBSR) program in Singapore. Continuing informal training (monthly training) from a meditation practitioner in Thailand.
**Context (e.g.: course year, theoretical lessons; internship)**	Ad hoc workshop and home practice
**Finding**	Six themes were derived from the data including: a) enhanced awareness of the present moment; b) increased serenity through mindfulness practice; c) triad of thoughts, emotion and behaviours; d) working towards interpersonal mindfulness; e) adapting mindfulness into nursing practice; and f) overcoming the challenges for mindfulness practice.<br>Extensive information from this research suggested that the MIND-NURSE PROGRAM has been beneficial for nursing students and can be integrated into undergraduate nursing curricula.<br>The program can also be extended to nursing students in other programs (Master's and Doctoral programs).<br>Future research may further test the effects of the MIND-NURSE program on other nursing programs.<br>Follow-up assessments can be measured to find out if the program has long-term effects on nursing students.
**Limits**	The results of this study may not be transferred to other contexts given that the participants were predominantly Chinese with few Malays and Indians. Future research should make more efforts to recruit from minority groups.
**If unclear content, the author of the correspondence has been contacted**	May 23, 2024
**He replied within the indicated time (one week)**	May 25, 2024<br><br>Integrate the sessions: Any adverse events; The skills of the facilitator
**Reference** [31]	Kou et al., 2022
**Country**	China
**Study Design**	RCT
**Sample Size**	60 Nursing students (E: 30- C: 30)<br>*Dropout rate: 3,33%*

**Dropout score**	E: -1; C: -1
**MBIs applied**	E: MBSR and MBCT.<br>Theoretical part: Introduction to the course; waking up the body; being and acting; feeling the present; recognizing the avoidance response; how to live with difficulties; being the master of your thoughts; mindfulness action; bringing awareness into your life.<br><br>Experiential part:<br>- Raisin meditation<br>- Body Scan<br>- Mindful breathing<br>- Three-minute breathing<br>- Awareness of a pleasant and unpleasant experience every day<br>- Mindful walking<br>- Mindfulness Meditation in Painful Feelings or Physical Tensions<br>C: Two-hour lectures on mindfulness in eight weeks<br>*Description of the intervention.*<br>Preparation session.<br>Argument. Introduction to the course.<br>Contents. Introduce awareness and group rules including respect, confidentiality, absence of judgment, common growth.<br>**Session 1.**<br>*Topic*. Wake up the body.<br>*Contents.* The raisin meditation; the scanning of the body.<br>*Homework.* Practice body scanning and jotting down perceptions; mindfully bring into daily life; practice mindful eating; practice journal compilation.<br>Session 2.<br>*Topic*. To be and to act.<br>*Content*. To experience the being and the modes of action through conscious breathing and body scanning.<br>*Homework. Practice*body scanning and noting perceptions; 10 minutes of mindful breathing; bringing mindfulness into daily life; awareness of a pleasurable experience every day.<br>**Session 3.**<br>*Topic*. Feel the present.<br>*Contents.* Disengage from nonsense and aimless thoughts through the mindfulness tract and three minutes of mindfulness breathing.<br>*Homework.* Practice of three-minute mindful breathing three times a day; awareness of an unpleasant experience every day.<br>**Session 4.**<br>*Topic*. Recognize the screwing response.<br>*Contents.* Mindfulness Meditation by Focusing on Negative Thoughts and Negative Moments<br>*Homework.* Practice mindful walking; practice mindful breathing three minutes a day.<br>**Session 5.**<br>*Topic*. How to live the difficulties.<br>*Contents.* Mindfulness meditation, learning to consciously deal with and accept negative emotions.<br>*Homework.* Practice three-minute breathing three times a day; mindful breathing when you encounter painful feelings or physical tension in daily life.

	**Session 6.**<br>*Topic*. Be the master of your thoughts.<br>*Contents.* Mindfulness Meditation by Exploring Thoughts and Emotions<br>*Homework.* Practice three-minute breathing three times a day; mindful breathing when encountering painful feelings or physical tensions in daily life; be mindful, recognize and register the patterns and signals of the body and mind when the mood is lowering.<br>**Session 7.**<br>*Topic*. Mindfulness action<br>*Contents.* Mindfulness Meditation: Interpreting Actions to Modulate Emotions<br>*Homework.* Practice of three-minute mindful breathing three times a day; mindful breathing when encountering painful feelings or physical tension in daily life.<br>**Session 8.**<br>*Topic*. Bring awareness into your life.<br>*Contents.* Body scan and retrospection on previous practices<br>*Homework.* Continue to practice mindfulness in everyday life.
**MBIs duration**	Two hours per session, one session per week for eight weeks.<br>Three-month follow-up.
**Mode of Delivery**	In person and at home
**Outcome(s)**<br><br>**Variable(s)**	- Implement verbal and non-verbal support communication skills;<br>- Developing emotional intelligence<br>- Developing human care
**Outcome(s) Measures**	Supporting Communication Scale (SCS)<br>Emotional Intelligence Scale (EIS)<br>Caring Ability Inventory (CAI)
**Any adverse events related to MBIs interventions**	NO
**Facilitator(s)**	The Researcher
**Facilitator's competencies**	Experienced mindfulness facilitator (>5 years) who has incorporated the principles of the Unified Mindfulness system into the training
**Context (e.g.: course year, theoretical lessons; internship)**	Extracurricular course in presence and at home
**Finding**	The mindfulness group showed higher total scores on all three scales than the control group (all ps < 0.05). For the subscales, the mindfulness group showed higher scores on the coaching and counseling subscale on the supportive communication scale and on the rating and expression of one's emotion subscale on the emotional intelligence scale. He indicated that mindfulness training could significantly improve supportive communication ability, emotional intelligence, and human caring ability. Eight-week mindfulness training could significantly improve nursing students' supportive communication skills, emotional intelligence, and human care ability. The effects of the training have been long-lasting and may persist for at least 3 months. Emotional intelligence causally influenced supportive communication, and the improvement of supportive communication was largely through the improvement of emotional

	intelligence, indicating the crucial role of emotional intelligence in the effectiveness of mindfulness training.
**Limits**	Relatively small sample size as the groups were small; Other factors such as cognitive reappraisal were not examined, which may be another potential mediator of the effect of mindfulness practice. Measurement of outcomes through self-reporting tools.
**If unclear content, the author of the correspondence has been contacted**	May 23, 2024
**He replied within the indicated time (one week)**	May 25, 2024.<br><br>The adverse events section has been integrated.
**Reference** [32]	Lam et al., 2024
**Country**	China
**Study Design**	MM
**Sample Size**<br><br>**Dropout score**	126 Nursing students. 39 students who attended the 2nd academic year; 87 students who attended the 3rd academic year -Nursing Degree.<br>*Dropout rate*: 12%<br>-15
**MBIs applied**	Mindfulness in Peer-assisted Learning (PAL).<br>Firstly, the programme adopted a mindfulness mobile application (app) developed by the university that provided Cantonese audio guides of various durations (5-40 minutes) to support mindfulness practice at home. Participants engaged in an online peer-assisted learning (PAL) mindfulness program that consisted of mindfulness practice, senior students sharing their experiences in the peer-assisted group. Peer leaders and nurses who had experience in mindfulness practice prepared 12 such videos, in which they offered clinical advice to aid students' preparation for clinical learning<br>In addition, student-led online peer support groups have been established to promote annual support, collaborative learning, and transition to clinical practice. Each group consisted of a peer leader and 10 peer learners who engaged in biweekly online discussions. These discussions followed a semi-structured format, covering topics such as mindfulness practice and reflections on clinical advice, sharing clinical positioning experiences, clinical preparation, and future plans for both clinical preparation and mindfulness practice<br><br>*Description of the intervention:*<br>Week 1.<br>Introduction to the workshop program; mindfulness practice "feeling the body"; Team greeting.<br>Week 2.<br>Mindfulness practice "feeling the body";<br>Video tutorial on clinical skills "how to be a good nurse"<br>Online discussion forum:<br>Introduction to the discussion forum; participants' expectations: basic rules; setting personal goals.<br>Week 3.<br>Mindfulness practice "feeling thoughts and emotions"

	Video tutorial on clinical skills "professional communication"<br>Week 4.<br>Video tutorial on clinical skills "knowledge preparation and stress management"<br>Online discussion forum: sharing the experience of mindfulness practice; leaders who share clinical practice.<br>Week 5.<br>Practice of awareness "compassion".<br>Video tutorial on clinical skills "skills preparation; conscious coping in clinical settings"<br>Week 6.<br>Mindfulness practice "integrated practice"<br>Video tutorial on clinical skills "the roles and responsibilities of the professional"<br>Online discussion forum: sharing the experience of mindfulness practice; Application of mindfulness in the clinical scenario<br>Week 7.<br>Mindfulness practice "integrated practice"<br>Video tutorial on clinical skills: "managing work effectively"<br>Week 8.<br>Mindfulness practice "integrated practice"<br>Video tutorial on clinical skills "sharing among nursing managers<br>Online discussion forum: sharing the experience of mindfulness practice; reflections on practice; Planning awareness and practice in the clinic in the future<br>Week 9 to Week 16.<br>Supporting study participants and clinical learning
**MBIs duration**	5-40 minutes for 8 weeks; NS supporting study (weeks from 9 to 16)
**Mode of Delivery**	Online workshops and audio guides at home.
**Outcome(s)**<br><br>**Variable(s)**	- Reduction in the level of depression<br>- Reduced anxiety level<br>- Reduced stress level<br>- Reduction of Burnout<br>- Increased level of perception of self-efficacy
**Outcome(s) Measures**	Depression Anxiety Stress Scale-21 (DASS-21)<br>Oldenburg Burnout Inventory – Student (OLBI-S)<br>General Self-Efficacy Scale (GSES)
**Any adverse events related to MBIs interventions**	NO
**Facilitator(s)**	Research Assistant and Peer Leader
**Facilitator's competencies**	The Research Assistant Trainer is a qualified mindfulness instructor who has extensive experience in providing mindfulness training to healthcare professionals.<br>The training covered the duties, responsibilities, and boundaries of peer leaders; communication skills; leadership and facilitation of discussion; the concept and practice of mindfulness; security and confidentiality; awareness and prevention of crises and suicide; and mental health support resources available at school and university.
**Context (e.g.: course year, theoretical**	Extracurricular peer to peer online course

**lessons; internship)**	
**Finding**	The program significantly improved participants' self-efficacy (β = 1.44, 95 percent confidence interval (CI): 0.58 to 2.30, p = 0.001) and decreased their level of burnout (β = -2.31, 95 % CI: -3.24 to -1.38, p < 0.001) but did not significantly alleviate their depression, anxiety, or stress over time (p > 0.05).<br>QUAL: topics under themes related to the experiences of the participants in the programme.<br>Theme 1: Nourishing the capacity for self-care through awareness.<br>Sub-themes: awareness of inner experience; create mental space with fast thoughts; manage academic stress; and emotions when facing clinical challenges.<br>Theme 2: Enhancing essential skills for the profession.<br>Sub-themes: understanding of the clinical environment; develop professional skills; build clinical judgment.<br>Theme 3: Maintaining momentum along with the challenges facing the profession.<br>Sub-themes: reliability and flexibility; incentive motivation; cognitive and social congruence; practical barriers.
**Limits**	The sample consisted of only one group, used a single-center study design, had a small sample size, and may have been subject to researcher bias. In addition, participants were selected to participate in the focus group interview based on a voluntary expression of interest, which could introduce a degree of homogeneity and potentially impact the richness and depth of the data collected. the perception that it is boring and useless, limit its effectiveness and successful implementation.
**If unclear content, the author of the correspondence has been contacted**	May 23, 2024
**He replied within the indicated time (one week)**	May 24, 2024.<br><br>Adverse events section integrated
**Reference** [33]	Pollard et al., 2020
**Country**	Canada
**Study Design**	RCT
**Sample Size**<br><br>**Dropout score**	120 nursing students who attended the 4th and final academic year- Nursing Degree<br>*Dropout rate*<br>-13 (11%)<br>E:86 – C: 21
**MBIs applied**	E: **Mindful moment.**<br>A 2-minute mindfulness-based guided intervention incorporated into the prebriefing component of the simulation.<br>This preparatory action became known as the "Mindfulness Moment". After completing the mindfulness-based intervention, participants in the treatment group moved on to simulation.<br>The Mindfulness Moments scripts varied weekly and targeted different bodily sensations (e.g., hearing, touch, smell, or taste) to facilitate the construction of greater awareness of oneself and the space they occupy in the world. After the simulation debriefing, both the

	treatment and control group participants had 10 minutes to complete the NASA-TLX. Typically, the survey took 2 minutes to complete.<br>*Example script.*<br>You are invited to do this exercise with your eyes open or closed. If you leave your eyes open, please lower your gaze. If I ask questions during this exercise, they are meant to guide your attention: no verbal response is required.<br>Draw attention to the breath. (Pause) Note the inhalation and note the exhale. (Pause) Notice the sensation of the breath as it enters the nose and then notice the breath as it comes out of the nose. (pause) Notice the rhythm of your breathing. Is it fast, slow, even, rough? (pause). Is there a pause between inhalation and exhalation, or between exhalation and inhalation, or does the breath occur without a pause? How is your breathing today? Let your focus shift to the sensations of the body. Today, notice what you are listening to (pause).<br>What sounds catch your attention? (pause) What sounds are close? (pause) What sounds are far away? (pause) Keeping your attention on your hearing, notice what you hear in the next minute of silence. (1 minute of silence)<br>(in silence...) Now bring the focus back to the breath.<br>In your free time, take two deep breaths, open your eyes, bring your attention back to the room, and get ready to participate in the workshop.<br><br>C: No intervention
**MBIs duration**	Two minutes before the three-hour, five-match simulation
**Mode of Delivery**	In presence
**Outcome(s)**<br><br>**Variable(s)**	Assessment of mental demands, physical demands, time demands, level of perceived exertion, performance and levels of perceived frustration.
**Outcome(s) Measures**	National Aeronautics and Space Administration Task Learning Index that measures mental demands, physical demands, time demands, effort, performance, and frustration
**Any adverse events related to MBIs interventions**	NO
**Facilitator(s)**	Course Facilitators
**Facilitator's competencies**	Script preparations and facilitator training by a mindfulness expert, Dr. Shelley Winton of Alberta Health Services for her leadership in developing the mindfulness-based intervention and training the investigators to deliver the mindfulness-based intervention.
**Context (e.g.: course year, theoretical lessons; internship)**	Before participating in the gestural simulation workshops.
**Finding**	The control group experienced significantly different perceived workload demands in two domains (temporal and exertion).
**Limits**	Results limited to this cohort.
**If unclear content, the author of the correspondence**	May 23, 2024

**has been contacted**	
**He replied within the indicated time (one week)**	May 24, 2024<br><br>Integration of the adverse events section
**Reference** [34]	Schwarze & Gerler, 2015
**Country**	USA.
**Study Design**	QE pre/post-test (case report)
**Sample Size**<br><br>**Dropout score**	5 nursing students. Dropout rate: -2 (40%)
**MBIs applied**	Modified Mindfulness-Based Cognitive Therapy (MBCT) Protocol: Pre-recorded guided meditations for body scans and breathing taken from the podcast Maddux and Maddux (2006).<br>https://itunes.apple.com/us/podcast/meditation-oasis/id204570355?mt=2
**MBIs duration**	Six one-hour sessions
**Mode of Delivery**	In presence with home activities
**Outcome(s)**<br><br>**Variable(s)**	- Reduced stress level<br>- Increased level of attention
**Outcome(s) Measures**	Perceived Stress Scale (PSS)<br>Mindful Attention Awareness Scale (MAAS)
**Any adverse events related to MBIs interventions**	NO
**Facilitator(s)**	Podcast authors are MM experts
**Facilitator's competencies**	Licensed Professional Counselor Trainer introduced the podcast
**Context (e.g.: course year, theoretical lessons; internship)**	Spring Semester
**Finding**	The results indicate that exposure to an MBCT intervention can have a positive impact on self-reported stress scores as measured by the PSS-10 and increase self-reported mindfulness scores as measured by the MAAS.
**Limits**	UOT drops, causes: unforeseen illnesses and time constraints (2/5 participants gave up)
**If unclear content, the author of the correspondence has been contacted**	May 31, 2024

**He replied within the indicated time (one week)**	June 3, 2024<br><br>Embedded sections dedicated to adverse events, Administered intervention MBIs, facilitator skills
**Reference** [35]	Uysal & Çalışkan, 2022
**Country**	Turkey
**Study Design**	QE pre/post-test
**Sample Size**<br><br>**Dropout score**	71 nursing students who attended the 1st academic year- Nursing Degree<br>Drop-out: 19 students<br>Sample: 52 students.<br>E: 17-C:35
**MBIs applied**	MBSR: MBSR practices included concentration on breathing, breathing and body exercises, body scanning, sitting meditation, mindful walking, mindful yoga, affective meditation, and creating a calendar of pleasant and unpleasant moments.<br>Starting in the second session, students were asked at the beginning of each session to share their experiences while doing their homework. They were questioned about situations in which they had difficulty carrying out the activities and what kind of awareness these activities gave them.
**MBIs duration**	Two hours two days a week for four weeks
**Mode of Delivery**	In presence with home activities
**Outcome(s)**<br><br>**Variable(s)**	- Reduced stress level<br>- Increased level of awareness
**Outcome(s) Measures**	Perceived Stress Scale (PSS)<br>Physio–Psycho–Social Response Scale (PPSRS)<br>Mindfulness Attention Awareness Scale (MAAS).
**Any adverse events related to MBIs interventions**	One student said that his mindfulness practice had increased his awareness, but that his stress level had also increased. He described himself as a person with a lot of anxiety and stress. However, he remained in the intervention group
**Facilitator(s)**	Researcher
**Facilitator's competencies**	Trained in the MBSR protocol
**Context (e.g.: course year, theoretical lessons; internship)**	Extra-Curricular Course
**Finding**	<u>RSS:</u> Increased awareness and decreased stress levels.
**Limits**	Small sample size. Convenience sample. Not general information to the nursing student population.
**If unclear content, the author of the correspondence has been contacted**	May 24, 2024

**He replied within the indicated time (one week)**	May 24, 2024<br><br>Integration of the session dedicated to adverse events
**Reference** [36]	Chen et al., 2013
**Country**	China
**Study Design**	RCT
**Sample Size**<br><br>**Dropout score**	60 Nursing students (E: 30-C: 30)
**MBIs applied**	E: Mindful breathing<br>C: No intervention<br>*Description of the intervention:*<br>On day 1, participants were taught how to focus on breathing with their eyes closed and to become non-judgmentally aware of their thoughts, feelings, and sensations while focusing on the flow of breath through their nostrils.<br>The underlying philosophy of mindfulness was also taught, emphasizing that participants should try to calm the mind by simply allowing invasive or discursive thoughts in the past, paying attention to the breath, and focusing on the dynamic sensations of the whole body.<br>On days 2-7, participants practiced mindfulness guided by 30 minutes of standardized instruction, modified according to Salmon et al. (2004).<br>This practice incorporated traditional Chinese Buddhist cultural concepts, such as an emphasis on using "inner eyes" to scan bodily sensations, according to the method used in an empirical study. Background music was played throughout the meditation process to help practitioners maintain concentration.
**MBIs duration**	30 minutes a day for 7 consecutive days
**Mode of Delivery**	In presence
**Outcome(s)**<br><br>**Variable(s)**	- Reduced anxiety<br>- Reduction of depression<br>- Evaluation of the effects on the activity of the autonomic nervous system (pA and HR)
**Outcome(s) Measures**	Self-Rating Anxiety Scale (SAS);<br>Self-Rating Depression Scale (SDS);<br>Heart rate; Blood Pressure
**Any adverse events related to MBIs interventions**	NS
**Facilitator(s)**	Senior Consultant Psychologist
**Facilitator's competencies**	Expert in the practice of Mindfulness meditation techniques
**Context (e.g.: course year, theoretical lessons; internship)**	Ad hoc workshop

**Finding**	The short mindfulness meditation has been helpful for Chinese nursing students in reducing anxiety symptoms and lowering systolic blood pressure and heart rate. Individuals with moderate anxiety are more likely to benefit from a short-term mindfulness meditation program.<br>The Mindfulness Meditation program is a non-pharmacological approach that teaches skills in the regulation of the emotional and autonomic nervous systems. It is effective for stress management and burnout reduction among nurses.<br>It can provide Chinese nursing students with an easy and effective way to cope with anxiety, reduce physical arousal brought on by anxiety, and achieve mental well-being and professional growth in their future careers.
**Limits**	Limitations of the study are not reported.
**If unclear content, the author of the correspondence has been contacted**	May 23, 2024
**He replied within the indicated time (one week)**	The email could not be delivered due to an unreachable address.
**Reference** [37]	Kang et al., 2009
**Country**	Korea
**Study Design**	RCT
**Sample Size**<br><br>**Dropout score**	41 nursing students (E: 21; C: 20)<br>*Dropout rate* 21,95%<br>E: -5; C: -4
**MBIs applied**	Stress Coping Program MBIs<br>**Session 1:**<br><u>Contents:</u><br>Presentation of the participants; Introduction of procedures; Stretching; Body scanning; inquiry.<br>**Session 2.**<br><u>Contents:</u><br>Questions and answers; stretching; body scanning; Share impressions<br>**Session 3.**<br><u>Contents:</u><br>Questions and answers; body scanning; stretching; conscious breathing; performance guide; Share impressions.<br>**Session 4.**<br><u>Contents:</u><br>Questions and answers; stretching; conscious breathing; gratitude; Share impressions.<br>**Session 5.**<br><u>Contents:</u><br>Questions and answers; stretching; musical meditation and happy memories; mindful walking; drive performance; Share impressions.<br>**Session 6.**<br><u>Contents:</u>

	Questions and answers; stretching; conscious breathing; mindful walking; Share impressions<br>**Session 7.**<br>Contents:<br>Questions and answers; stretching; body scanning; sitting meditation; meditation of gentle loving-kindness; Sharing impressions<br>**Session 8.**<br>Contents:<br>Questions and answers; my funeral; sitting meditation; share impressions; new beginning.
**MBIs duration**	90 minutes per session for eight weeks (one session per week)
**Mode of Delivery**	In presence
**Outcome(s)**<br><br>**Variable(s)**	- Stress reduction<br>- Reduced anxiety<br>- Reduction of depression<br>- Evaluation of the effects on the activity of the autonomic nervous system (pA and HR)
**Outcome(s) Measures**	Psychosocial wellbeing index-short form (PWI-SF)<br>State Trait Anxiety Inventory (STAI)<br>Beck depression inventory (BDI)
**Any adverse events related to MBIs interventions**	NS
**Facilitator(s)**	The researcher
**Facilitator's competencies**	Professional training in mindfulness meditation with eight years of conducting experience.
**Context (e.g.: course year, theoretical lessons; internship)**	Ad hoc workshop during the internship
**Finding**	A significant difference in stress scores ($F=6.145$, $p=0.020$);<br>A significant difference in anxiety scores ($F=6.985$, $p=0.013$)<br>No significant difference in depression scores ($t=1.986$, $p=0.056$), HR, PA
**Limits**	Small sample size. No blindness.
**If unclear content, the author of the correspondence has been contacted**	May 23, 2024
**He replied within the indicated time (one week)**	Email address unreachable
**Reference** [38]	Song & Lindquist, 2015
**Country**	Korea
**Study Design**	RCT

**Sample Size**<br><br>**Dropout score**	50 nursing students E: 25- C: 25<br>*Dropout rate: 12%*<br>E: -4; C:- 2
**MBIs applied**	E: MBSR Protocol: Yoga, Sitting Meditation, Mindful Walking Meditation, Breath-Work, Body Scanning, and Eating Meditation<br>C: wait list
**MBIs duration**	Two hours a week for eight weeks
**Mode of Delivery**	In presence with home activities
**Outcome(s)**<br><br>**Variable(s)**	- Reduction in the level of depression<br>- Reduced anxiety level<br>- Reduced stress level<br>- Increased level of awareness
**Outcome(s) Measures**	Anxiety and Stress Scale-21 (DASS-21)<br>Mindfulness Attention Awareness Scale (MAAS)
**Any adverse events related to MBIs interventions**	NS
**Facilitator(s)**	Mindfulness Instructor
**Facilitator's competencies**	Over 10 years of experience in MSR
**Context (e.g.: course year, theoretical lessons; internship)**	Extra-Curricular Course
**Finding**	Compared to WL participants, MBSR participants reported significantly greater decreases in depression, anxiety, and stress, and a greater increase in mindfulness. The average anxiety in the MBSR group was reduced by 3.9 points, while scores in the WL group remained unchanged; there was a statistically significant difference between the two groups ($F = 5.61$, $df = 1$, $p = .023$). The average stress in the MBSR group decreased by 27.1 and stress decreased by 16.3 in the WL group; there was a statistically significant difference between the groups ($F = 15.31$, $df = 1$, $p < .001$). The effect of MBSR on mindfulness was also significant. Awareness increased by an average of 10.8 in the MBSR group, compared to 1.3 in the WL group ($F = 5.03$, df = 1.p = .010).
**Limits**	Small sample size. Non-generalization of results
**If unclear content, the author of the correspondence has been contacted**	May 24, 2024
**He replied within the indicated time (one week)**	Email address unreachable.
**Reference** [39]	Alhawatmeh et al., 2022
**Country**	Jordan

**Study Design**	RCT
**Sample Size**<br><br>**Dropout score**	112 nursing students E: 56; C: 56<br>*Dropout rate:* (3,57%)<br>E: -2; C: -2
**MBIs applied**	E: Mindfulness Meditation<br>C: Sitting quietly and with your eyes closed<br>*Description of the interventions.*<br>(Open) meditation "of the senses" (ABC *Relaxation Theory protocol* by Smith https://psycnet.apa.org/record/2005-13005-000);<br>Participants were asked to sit upright in a comfortable position, place their feet on the floor, and silently observe and reflect on internal and external stimuli such as breathing, thinking, feeling, physical sensation, and sound, without reactions, judgments, or evaluations.<br>They were asked to pay attention, write down and silently let go of all internal and external stimuli.<br>They were reminded that they should not try to think, reject, or do anything with these stimuli, and that they should not focus on understanding the connections between stimuli. They were asked to simply let each stimulus come and go and wait for the next stimulus. Finally, participants were told that they should not worry about distractions and that, when faced with a distraction, they should notice it as yet another passing stimulus (e.g., *"Ah, a distraction... what an interest")*
**MBIs duration**	3 hours of plenary to explain the study;<br>5 weekly sessions of 30 minutes of mindfulness meditation<br><br>The timing of the control group's sessions was similar to that of the experimental groups
**Mode of Delivery**	in presence
**Outcome(s)**<br><br>**Variable(s)**	- Stress reduction<br>- Increased levels of dispositional mindfulness (DM)<br>- Reduction of Cortisol and C-Reactive Protein
**Outcome(s) Measures**	Mindfulness Attention Awareness Scale (MAAS); Perceived Stress Scale (PSS) and Biomedical Markers: ELISA methods for Cortisol and CRP (C-Reactive Protein)
**Any adverse events related to MBIs interventions**	NS
**Facilitator(s)**	NS
**Facilitator's competencies**	NS
**Context (e.g.: course year, theoretical lessons; internship)**	Ad hoc workshop
**Finding**	The results showed that mindfulness meditation (MM) was significantly effective in reducing serum cortisol levels and perceived stress. MM also reduced CRP levels and

	increased the disposition to Mindfulness even if, in the latter two cases, the outcomes did not reach statistically significant levels.<br>These findings underscore the need for serious consideration for MM in nursing schools to reduce stress and increase immunity in this vulnerable population.
**Limits**	convenience sampling (on a voluntary basis). No blindness. No follow up
**If unclear content, the author of the correspondence has been contacted**	May 23, 2024
**He replied within the indicated time (one week)**	NO
**Reference** [40]	Alsaraireh et al., 2017
**Country**	RCT
**Study Design**	Jordan
**Sample Size**<br><br>**Dropout score**	200 Nursing students E: 100; C: 100<br>*Dropout rate*<br>(9,5%)<br>E: -9; C: -10
**MBIs applied**	E: Mindfulness Meditation<br>C: Physical exercises<br>*Description of the interventions.*<br>E: Each session contained three different techniques, 20 minutes each for mindful breathing, mindful body scanning, and mindful walking.<br>During the breathing section, participants were asked to assume a sitting position, breathe naturally, and focus their attention on inhaling and exhaling.<br>During the walking section, they moved back and forth between two points 10 meters apart, starting slowly for 5 minutes, then slower for another 5 minutes, and rather slowly for the rest of the time.<br>During the body mental scan section, they were instructed to sit on a mat, cover themselves with a blanket, and rest their heads on a pillow; Then, go through the whole body with awareness from top to bottom and notice sensations such as pain, non-acceptance, or tension in any part.<br><br>C: Gym classes. The exercises were of three types:<br>stretching for 20 minutes, followed by 20 minutes of aerobics and then another 20 minutes of strength exercises.<br>The stretch included 2 minutes each of running, a lateral stretch in a standing position, lean forward and lunges down with 1 minute of stretching at a right angle.<br>A 2-minute break between two sets was allowed.<br>Aerobic exercises included 2 minutes each of jumping rope, bunny hop (a forward jump in a squatting position) and lateral delimitation, and 3 minutes of jumping jumps.<br>Aerobic exercise was repeated, with a 2-minute break between the two sets.<br>Strength exercises included flexion exercises for 2 minutes, squat exercises for 4 minutes, T-rotation plank exercises for 2 minutes (1 minute per side), and reverse lunge with front twist for 2 minutes. A 1-minute break was allowed between each minute of exercise.

**MBIs duration**	1 hour for 3 days a week for 10 weeks for the intervention group and the control group.
**Mode of Delivery**	in presence
**Outcome(s)**<br><br>**Variable(s)**	Reduction in the level of depression
**Outcome(s) Measures**	Center for Epidemiologic Studies Depression Scale (CESD-R)
**Any adverse events related to MBIs interventions**	NS
**Facilitator(s)**	NS
**Facilitator's competencies**	NS
**Context (e.g.: course year, theoretical lessons; internship)**	Ad hoc workshop
**Finding**	In the post-test, both groups showed a significant decrease in depression-related scores; However, mindfulness meditation participants showed a significantly greater reduction in their depression score than the group that exercised physically, T(179) = 5.2; p = .00.
**Limits**	convenience sampling. No blindness. No follow up
**If unclear content, the author of the correspondence has been contacted**	May 23, 2024
**He replied within the indicated time (one week)**	NO
**Reference** [41]	Burger & Lockhart, 2017
**Country**	Hawaii
**Study Design**	RCT
**Sample Size**<br><br>**Dropout score**	60 nursing students (E: 32; C: 28)<br>*Dropout rate:* (13,33%) E: -4 C: -4
**MBIs applied**	E: Mm Online<br>C: wait list

	E: Students received online instruction on Mm techniques and guidelines for performing a daily 10-minute meditation practice. In addition, an audio file of a professional-led Mm was included for participants to use on their personal computers or smartphones
**MBIs duration**	10 minutes a day for 4 weeks
**Mode of Delivery**	online<br>University of Wisconsin Podcast- Department of Public Health<br>https://www.fammed.wisc.edu/mindfulness-meditation-podcast-series/
**Outcome(s)**<br><br>**Variable(s)**	- Improve attention level<br>- Stress reduction<br>- Increased DM levels
**Outcome(s) Measures**	Attention Network Test (ANT)<br>Perceived Stress Scale (PSS)<br>Five-Facet Mindfulness Questionnaire (FFMQ)
**Any adverse events related to MBIs interventions**	NS
**Facilitator(s)**	Senior Researcher
**Facilitator's competencies**	trained as a mindfulness facilitator and daily practice
**Context (e.g.: course year, theoretical lessons; internship)**	Online at home in the first half of the year
**Finding**	After 4 weeks of meditation, students in the MM group showed greater executive attention efficiency, lower perception of stress, and better awareness, compared to the control group<br>Participants in the MM group had a greater improvement in executive attention than those in the control group with an adjusted mean difference of 17.4 (SE = 8.42).<br><br>The Mm group had significantly higher scores on measures of mindfulness characteristics ($p = 0.013$), as well as significantly lower perceived stress ($p = 0.000$), after 4 weeks of meditation compared to participants in the control group.
**Limits**	An a priori sample size (N = 82) was calculated for a mean effect size of 0.25, alpha of 0.05, and power of 0.95, but in the end, only 52 students completed the study, thus reducing post hoc power. The use of face-to-face MM sessions, along with individual practice, can encourage future participation and retention. Some restriction of results is also possible, as 14% of meditators reported a lack of complete adherence to treatment (meditating only 3 or 4 days a week. In addition, there is a need to recruit more diverse groups of participants to determine the effect of culture on study variables.
**If unclear content, the author of the correspondence**	May 23, 2024

**has been contacted**	
**He replied within the indicated time (one week)**	NO
**Reference** [42]	Can Gür & Yilmaz, 2020
**Country**	Turkey
**Study Design**	RCT
**Sample Size**<br><br>**Dropout score**	132 nursing students<br>*Dropout rate* (7%)<br>-9 nursing students<br>E: 61- C: 62
**MBIs applied**	E: Mindfulness-Based Empathy Training (MBET)<br>C: No intervention<br><br><u>*Description of the intervention:*</u><br>Mindfulness-Based Empathy Training (MBET) program.<br><u>Formal practices</u> in the classroom and at home included mindful breathing, body scan meditation, thought awareness, and raisin meditation.<br><u>Informal practices</u> included mindfulness in activities of daily living, communication, autopilot, and presence.1) Study information: To provide information about the time and location of sessions, program application, and group rules (2 hours, 1 day per week).<br><br>2) Aging and characteristics of old age: to provide students with information on the definition of old age and the characteristics of cognitive, social and emotional development in old age (2 hours, 1 day per week).<br><br>3) Basic communication concepts, verbal and non-verbal communication: Provide information about communication and factors affecting communication in older individuals (4 hours, 2 days a week).<br><br>4) Empathy and its importance: learn the definition of empathy, the levels of empathic response, the importance of empathy in interpersonal communication and work in the elderly (4 hours, 2 days a week).<br><br>5) Giving information about mindfulness: Giving information about mindfulness breathing exercise, mindfulness in daily living activities, mindfulness of thinking and dried grape meditation and teaching their application (4 hours, 2 days a week).<br><br>6) Body Scan Meditation: To allow participants to direct their awareness of their current mental and physical events and connect with them (4 hours, 2 days a week).<br><br>7) Accept and allow: Learn that accepting and dealing with others is an important aspect of empathy (4 hours, 2 days a week).<br><br>8) Awareness of the judgmental mind: To ensure that participants are aware of their judgments and observe them without interpreting them (4 hours, 2 days a week).

	9) Effective/Empathic/Active Listening: To ensure that participants practice proper listening (4 hours, 2 days a week).
**MBIs duration**	8 weeks: one hour twice a week for a total of 16 meetings
**Mode of Delivery**	in presence and online traces at home
**Outcome(s)**<br><br>**Variable(s)**	- Increased Empathy Levels<br>- Reduction of attitudes to age discrimination (aging)
**Outcome(s) Measures**	Jefferson Empathy Scale (JSENS)<br>Age Discrimination Attitude Scale (ADAS)
**Any adverse events related to MBIs interventions**	NS
**Facilitator(s)**	First author
**Facilitator's competencies**	NS
**Context (e.g.: course year, theoretical lessons; internship)**	Ad hoc workshop
**Finding**	The analyses revealed that participants in the training group reported a significant increase in the level of empathy compared to the control group. No significant change in total age discrimination scores was recorded in either group.
**Limits**	Due to the nature of the study, it was impossible to blind the intervention to the participants. The questionnaires used in the study do not measure the impact of the program on participants' actual clinical experiences and relationships with patients or the healthcare team. They only show the perceptions of the participants. We have only assessed the effect of the programme in the short term. A longer-term, comprehensive study is needed to assess the permanence of the program's impact on empathy and discrimination.
**If unclear content, the author of the correspondence has been contacted**	May 23, 2024
**He replied within the indicated time (one week)**	NO
**Reference** [43]	Chase-Cantarini & Christiaens, 2019
**Country**	USA
**Study Design**	QL

**Sample Size**<br><br>**Dropout score**	64 nursing students. *Dropout rate*: Not specified
**MBIs applied**	Mindfulness moments (each lesson a different intervention):<br>- Mindful breathing<br>- Mantra<br>- Music<br>- Progressive muscle relaxation<br>- Well-being remembered<br>- Body Scan<br>- Guided images<br>- Loving-kindness<br><br>*Description of the intervention:*<br>Concentration on the breath:<br>Invite students to notice what it feels like to inhale and exhale, observing the movement of the muscles, the air moving through the nostrils, posture, while releasing judgment and expectations.<br>When your mind wanders, call it back to observe your breath.<br>Mantra-based meditation:<br>Repeat a symbol, word, or phrase during mindfulness meditation.<br>Students choose a mantra to repeat, such as "Love" or "I am at peace" or "Om" or a positive affirmation.<br>When the mind wanders it returns to focus on the chosen mantra.<br>Music-Based Meditation:<br>Have students focus their minds on the music during mindfulness meditation. Use slow, peaceful music with no lyrics. When your mind wanders, focus on the music.<br>Progressive muscle relaxation:<br>Verbally guides the student to tense and then relax one large muscle group at a time, noting the difference between tension and relaxation.<br>Remembered Well-being:<br>Verbally guides students to remember and focus on a time when they were in optimal health.<br>Engage all five senses by asking students to notice how optimal well-being felt physically, emotionally, spiritually, socially, cognitively, etc.<br>Body scan:<br>With each inhale, have students mentally scan their bodies from finger to finger, looking for areas of tension. With each exhale, release tension from head to toe.<br>Guided Imagery (Special Place):<br>Verbally guides students through an imaginary scenario that involves all five senses.<br>For example: Have students create a "special place" in their imagination and notice what they hear, see, hear, smell, and taste while in the imaginary place.<br>Loving-kindness:<br>Verbally guide students to focus on feelings of compassion and wish others well.<br>For example: Ask students to imagine other living things, and silently repeat three times, "May she live with ease, happiness, and good health."
**MBIs duration**	5-10 minutes before the theory lesson every week of a semester
**Mode of Delivery**	in presence before the lesson

**Outcome(s)**<br><br>**Variable(s)**	- Reduced stress and fatigue<br>- Increased levels of engagement, focus, and learning
**Outcome(s) Measures**	The survey asked students to comment on four open-ended questions about the course: what was going well, what wasn't going well, suggestions for improvement, and feedback on moments of mindfulness.
**Any adverse events related to MBIs interventions**	NS
**Facilitator(s)**	The two authors
**Facilitator's competencies**	nurse educators without specific training
**Context (e.g.: course year, theoretical lessons; internship)**	Before the theoretical lessons
**Finding**	59 (93.6%) of students provided positive comments such as "I love it", "I really like it" and "it's a nice time to relax". Only one student answered in the negative, stating, "Not a fan." Six students suggested that mindfulness moments should be longer, and three students suggested doing mindfulness moments at the end of class so they could go home relaxed. Five students said it made them tired or sleepy. Other themes included the appreciation of being introduced to mindfulness meditation. Several students said they had used relaxation methods in other areas, such as while standing in line at the grocery store or studying for exams in other classes.
**Limits**	The fear of teachers to teach mindfulness techniques. The teachers had no specific training. Using resources on the web could entice teachers to start the lesson with a few minutes of Mm.
**If unclear content, the author of the correspondence has been contacted**	May 25, 2024
**He replied within the indicated time (one week)**	NO
**Reference** [44]	Cheli & De Bartolo, 2020
**Country**	Italy
**Study Design**	RCT
**Sample Size**<br><br>**Dropout score**	82 nursing students (E: 36; C: 46)<br>*Dropout rate*<br>*(8.53%)*<br>*E: -2; C: -5*

<table>
<tr><td>MBIs applied</td><td>E: Mindfulness Meditation
C: Normal Educational Practice
<u>Description of the intervention</u>
MBEP (Mindful Based Educational Program) called Self-Aware Nurse Project (SANP):
The table shows the mindfulness techniques administered to the control and intervention group which also included practices of compassion and loving-kindness

<table>
<tr><th></th><th>GPC TRAINING</th><th>MINDFULNESS EXERCISE</th><th>MINDFULNESS PSYCHOEDUCATION</th></tr>
<tr><td colspan="4">PERSONAL DIMENSION</td></tr>
<tr><td>Week 1</td><td>Constructivist Theories of Education- Introduction</td><td>Autopilot; sitting meditation; Standing yoga</td><td>Mindfulness practice and programs; Autopilot</td></tr>
<tr><td>Week 2</td><td>Coping and stress patterns</td><td>Autopilot; the day I suffered; Meditation on the breath</td><td>Mental wandering; Thoughts and feelings</td></tr>
<tr><td colspan="4">Relational dimension</td></tr>
<tr><td>Week 3</td><td>Constructivist Theories of Education - Plaget & Bruner</td><td>Autopilot; meditation on the breath; Standing yoga</td><td>Automatic thoughts; acceptance</td></tr>
<tr><td>Week 4</td><td></td><td>meditation on the breath; sitting meditation; The compassion that flows out</td><td>Psychological flexibility; compassion</td></tr>
<tr><td colspan="4">Organizational size</td></tr>
<tr><td>Week5</td><td>Burnout patterns in the health professions</td><td>sitting meditation; three pillars of death; Mindful walking</td><td>Self-compassion; loving-kindness</td></tr>
<tr><td>Week 6</td><td>Healthcare Communication Model</td><td>sitting meditation; loving-kindness; The compassion that flows out</td><td>Compassionate mind</td></tr>
<tr><td colspan="4">Day of Silence</td></tr>
<tr><td>Final session</td><td>Nobody</td><td>Autopilot; sitting meditation; seated yoga; meditation on the breath; walking consciously; loving-kindness; the compassion that flows; compassion flowing; breathing group</td><td>summary</td></tr>
</table>
</td></tr>
<tr><td>MBIs duration</td><td>6-week MBEP:
- 5 regular sessions of 3 hours
- 1 session: 4.30 hour lesson that was based in part on the tradition of mindfulness-based stress reduction, along with some components of mindfulness-based cognitive therapy and compassion-focused therapy</td></tr>
<tr><td>Mode of Delivery</td><td>In presence</td></tr>
</table>

**Outcome(s)**<br><br>**Variable(s)**	- Acceptability of the intervention (MBEP) by nursing students (10% Drop-out)<br>- The proposed MBEP is effective in reducing burnout<br>The proposed MBEP is effective in increasing dispositional mindfulness
**Outcome(s) Measures**	Copenhagen Burnout Inventory<br>Mindful Attention Awareness Scale
**Any adverse events related to MBIs interventions**	NS
**Facilitator(s)**	NS
**Facilitator's competencies**	Specific training and supervision, at least 2 years of experience as a Mindfulness teacher
**Context (e.g.: course year, theoretical lessons; internship)**	Replacement of a pre-existing course in General Pedagogy, with a tailor-made MBIs, delivered in conjunction with the final internship.<br>Standard curricular objectives:<br>(a) promote reflexivity as a general ability to monitor personal experience<br>(b) provide strategies and tools to promote health education in patients.<br>Students in the third and final year of the course.
**Finding**	Effectiveness was defined as an increase in dispositional mindfulness and a decrease in perceived burnout at the end of the standard 3-month internship in a statistically significant manner compared to the control group.<br>The dropout rate < to 10% may indicate a good level of acceptability of the intervention by the study cohort
**Limits**	- low sample size once Drop-out are excluded (no. 75)<br>- absence of a randomization of subjects<br>presence of only two groups
**If unclear content, the author of the correspondence has been contacted**	NN
**He replied within the indicated time (one week)**	NN
**Reference** [45]	Colburn, Guerrazzi-Young & Durant, 2023
**Country**	USA
**Study Design**	QL
**Sample Size**<br><br>**Dropout score**	69 nursing students<br><br>Dropout rate: not specified
**MBIs applied**	Mindful Self-Compassion Workshop<br>*Description of the intervention:*<br>sandtray constructions, lectures and training on the principles and techniques of mindfulness and self-compassion, and a discussion on trauma-informed principles of care<br>**Protocol:**<br>4 hours of conscious self-compassion workshop

	1-Self-kindness 2- Recognizing shared humanity/human experience 3- Mindfulness<br>- Sand Tray Construction: "Intention Tray" - Clinical Instructor -2<br>- Sandtray build: "tray for reflection and affirmation of strength" – participants-2<br>- Fundamentals: Window of Tolerance – framework for understanding brain/body reactions to emotional stress<br>- Self-compassion practices: Yin/Yang of Compassion; words of affirmation-1<br>- Mindfulness Practices: Grounding, Breathing, Reflection, Mindful Movement-3<br>- Sand Tray Construction: "Cases in the Sand" – Participants-2<br>- Advanced: Principles of Trauma-Informed Care: How This Allows You to Present Yourself Better to Your Patients
**MBIs duration**	4-hour workshop
**Mode of Delivery**	In presence
**Outcome(s) Variable(s)**	The Intervention Participants' Perceptions of Using Sandtray as a Tool to Teach Conscious Self-Compassion
**Outcome(s) Measures**	Word cloud analysis to answer questions related to:<br>- their thinking about the standtray<br><br>The impact of the experience
**Any adverse events related to MBIs interventions**	NS
**Facilitator(s)**	Experimenter
**Facilitator's competencies**	A Certificate of Advanced Practice in Mind-Body Medicine
**Context (e.g.: course year, theoretical lessons; internship)**	Workshop part of the curriculum of nursing students
**Finding**	The thematic analysis indicated that participants overwhelmingly appreciated the sandtray component, finding it useful for reflecting on strengths, life, emotions and experiences, and for expressing themselves. In addition, the sandtray component was indicated by the participants as the most impactful practice offered in the workshop.
**Limits**	- Small sample size lack of qualitative methodological rigor
**If unclear content, the author of the correspondence has been contacted**	NN
**He replied within the indicated time (one week)**	NN

**Reference** [46]	Dai et al., 2022
**Country**	China
**Study Design**	RCT
**Sample Size**<br><br>**Dropout score**	120 nursing students (E: 60- C: 60)<br>Drop-out: 10%<br>E: -8; C: -4
**MBIs applied**	E: Mindfulness Living with Challenge (MLWC)<br>C: Health Education<br><br>*Description of the intervention:*<br>gee.<br>**Week 1.**<br>Practice:<br>Conscious eating; conscious breathing<br>Contents:<br>Introduction to mindfulness; how to incorporate mindfulness into daily life; evidence on interventions based on Mindfulness.<br>Home activities:<br>Practices of mindfulness of daily life and mindful breathing for 5-10 minutes a day, seven days a week.<br>**Week 2.**<br>Practice:<br>Body scanning; three-minute breathing<br>Contents:<br>Introduction to the way of acting and being of the mind; instruct participants to speak with their bodies.<br>Home activities:<br>Body scanning; three-minute breathing; Compilation of the calendar of pleasant and unpleasant experiences, respectively for 10-15 minutes a day for seven days a week<br>**Week 3.**<br>Practice:<br>Sitting meditation (mountain meditation and mindful sleep meditation); mindful walking; sitting meditation (meditation on the lake).<br>Contents:<br>Introduction to the scientific understanding of sleep, and to sleep hygiene and to seven attitudes of mindfulness practice.<br>Home activities:<br>Sitting meditation and mindfulness clock for 15 minutes a day, seven days a week.<br>**Week 4.**<br>Practice:<br>Mindful walking; Sitting meditation (meditation on the lake)<br>Contents:<br>Introduction to mindful life with thoughts using the "STOP" and "RAIN" principle to deal with a storm of thoughts and emotions<br>Home activities:<br>practice of mindfulness listening; mindful walking; STOP/RAIN for 15-20 minutes a day for seven weeks.<br>**Week 5.**<br>Practice:<br>Mindful Yoga<br>Contents:<br>Introduction to conscious movement to relax the body and mind; explanation of identifying the avoidance response allowing you to let go.<br>Home activities:

	Sitting meditation for 15 minutes a day seven days a week and practicing mindful yoga three times a day as appropriate.<br>**Week 6.**<br>Practice:<br>Sound meditation; meditation of love and kindness<br>Course unit contents:<br>How to balance daily life; conscious living is a challenge; Living in the present<br>Home activities:<br>And list the activities that nourish and those that consume and weave the parachute of awareness
**MBIs duration**	variable per intervention; 30-40 minutes per lesson (two lessons per session) for 6 weeks
**Mode of Delivery**	Online
**Outcome(s)**<br><br>**Variable(s)**	- Reduction of depression<br>- Reduced anxiety<br>- Stress reduction<br>- DM Level Boost<br>- Increased level of perceived social support
**Outcome(s) Measures**	DASS-21 depression-anxiety-stress scale;<br>Short Formed Five Facets of Mindfulness Questionnaire (FFMQ-SF) PSSS Perceived Social Support Scale
**Any adverse events related to MBIs interventions**	NS
**Facilitator(s)**	Two psychiatrists
**Facilitator's competencies**	Certified Mindfulness Facilitator at Mindfulness Awareness Research Center California
**Context (e.g.: course year, theoretical lessons; internship)**	Extracurricular course
**Finding**	The online mindfulness intervention implemented in this study is effective in improving mental health among undergraduate nursing students interested in this intervention: statistically significant reduction of anxiety, stress, and improvement in level of awareness. depression not statistically significant
**Limits**	Further research could be planned on multicenter studies to confirm the effect of MLWC intervention on undergraduate nursing students and other populations
**If unclear content, the author of the correspondence has been contacted**	May 23, 2024
**He replied within the indicated time (one week)**	NO
**Reference** [47]	Ebrahem M et al., 2022
**Country**	Egypt

**Study Design**	QE
**Sample Size**<br><br>**Dropout score**	78 nursing students who attended the 1st academic year-Nursing Degree<br>*Dropout rate*<br>*-1 (1,28%)*
**MBIs applied**	Theoretical part: obsessive compulsive disorders, coping methodologies, mindfulness.<br><br>Experiential part:<br>- Mindful breathing<br>- Muscle and progressive relaxation
**MBIs duration**	NS
**Mode of Delivery**	NS
**Outcome(s)**<br><br>**Variable(s)**	- Increased self-efficacy<br>- Reduction of suicidal ideation
**Outcome(s) Measures**	Arabic obsessive-compulsive scale (AOCS);<br>Suicidal Ideation Scale ;<br>Self-efficacy scale;<br>Mindfulness Scale
**Any adverse events related to MBIs interventions**	NS
**Facilitator(s)**	NS
**Facilitator's competencies**	NS
**Context (e.g.: course year, theoretical lessons; internship)**	NS
**Finding**	Mindfulness skills had a positive effect on improving self-efficacy and decreasing suicidal ideation and obsessive-compulsive symptoms among first-year nursing students during the COVID-19 pandemic. Further study is needed in all nursing faculties for the early identification of students with obsessive-compulsive symptoms and to help them overcome their difficulties using mindfulness skills.
**Limits**	Small sample size. Non-generability of results. Failure to randomize
**If unclear content, the author of the correspondence has been contacted**	NP
**He replied within the indicated time (one week)**	-
**Reference** [48]	ElKayal & Metwaly, 2022
**Country**	Egypt
**Study design**	QE

**Sample**	160 nursing students (8 groups of 20 students)
**Mindfulness-based intervention applied**	Theoretical part:<br>Concept of stress and post-traumatic stress; the causes and signs and symptoms; mindfulness its effects; Mindfulness practices.<br><br>Experiential part:<br>- Body Scan<br>- Conscious movement<br>- Sitting meditation<br>- Standing meditation<br>- Mindful breathing<br>- Yoga<br>- Muscle relaxation<br>*Description of the intervention:*<br>"The sessions were as follows:<br>First sessions (45-60 min): The researchers presented an overview of the intervention in this session, including the goal of the intervention, the number of sessions, the duration of each session, and the benefits of the application, as well as the meeting locations and times for each group. The pretest was then administered using the data collection sheet.<br>The second session: In this session, we discussed the concept of stress, as well as the causes, nature, and effects of psychological trauma, and what the signs and symptoms of post-traumatic stress are like. Students were instructed on homework in the form of worksheets that contain information about PTSS, the expression of negative feelings, and how to document them in the worksheet.<br>The third session: After reviewing the previous session, students were provided with knowledge of the signs and symptoms of post-traumatic stress and explained effective ways to cope with these symptoms such as breathing exercises, yoga, and meditation.<br>Fourth session: During this session, the researchers provided information about mindfulness and its effect on the brain, as well as different types of mindfulness practices such as body scanning (a process of shifting attention through the body); mindful movement, sitting meditation; and walking meditation.<br>Fifth session: Breathing exercises, yoga, muscle relaxation and mindfulness practice were then taught using a combination of video modelling and practice during the session. A homework assignment was created in collaboration with the students to allow them to practice these skills each day until the next session.<br>Sixth Session: A discussion from the previous session was followed by video presentations that clarified different types of meditation practice and trained students on rain and sea meditation.<br>Seventh session: All completed tasks were reviewed as tasks. Information is then provided on common problems associated with PTSD, for example, depression, guilt, and how to overcome them.<br>Eighth Sessions: This session included a summary of all sessions, identification of student opinions, and comments on the benefits of the sessions. It also included communication channels and thanking students for their advice and suggestions."
**Time of intervention administration**	45-60 minutes per session for a total of 8 sessions in three months

**Method of intervention administration**	In presence
**Primary outcome**	- Reduction of post-traumatic stress<br>- Increased DM
**Assessment Tool**	Event Impact Scale (IES-R)<br>15-item Five-Facet Awareness Questionnaire (FFMQ-15)
**Any adverse events related to MBIs interventions**	NS
**Who administers the MBIs interventions**	Researchers
**Facilitator's competencies**	To be able to deliver this intervention, the researchers received training in mindfulness-based stress reduction techniques (MBSR) at the Zagazig University Psychiatric Center, 6 hours a day for 3 days, which included theoretical and practical training.
**The context (e.g.: course year, theoretical lessons; internship)**	Ad hoc workshop
**Conclusion**	In light of the present study, it can be concluded that emergency nursing students experience different levels of PTS symptoms in the pre-intervention period, but that these symptoms improved significantly after the application of mindfulness interventions. In addition, the level of awareness improved after the intervention. Symptoms of post-traumatic stress are positively correlated with the number of hours of training and negatively correlated with the student's age, the mother's occupation and the level of education.
**Limits**	Because of their preoccupation with lectures and hands-on training in hospitals, students have difficulty finding time to apply mindfulness intervention.
**If unclear content, the author of the correspondence has been contacted**	May 23, 2024
**He replied within the indicated time so as to make the changes**	NO
**Reference** [49]	Franco, 2022
**Country**	USA
**Study Design**	QE pre/post-test
**Sample Size**<br><br>**Dropout score**	76 nursing students who attended the final-year- Nursing Degree<br><br>Dropout rate not specified

**MBIs applied**	TAO website: content focused on different mindfulness strategies, such as:<br>- Mindful breathing<br>- Letting go<br>- Take a holiday with your mind<br>- Mindful walking<br>- Notice yourself.<br>https://www.taoconnect.org/
**MBIs duration**	12 mindfulness exercises lasting from two to 11 minutes for a total of four weeks
**Mode of Delivery**	Online
**Outcome(s)**<br><br>**Variable(s)**	- Reduction of depression<br>- Stress reduction<br>- Reduced anxiety<br>- Increased level of positive coping<br>- Assess the impact of the experience
**Outcome(s) Measures**	Stress and Depression subscales of the Depression Anxiety Stress Scales (DASS-21)<br>Coping Strategies Inventory-Short Form (CSI-SF)
**Any adverse events related to MBIs interventions**	NS
**Facilitator(s)**	Course Facilitator (Interactive Site)
**Facilitator's competencies**	Therapy Assistance Online (TAO) is a peer-reviewed interactive website developed by Dr. Susan Benton in 2012
**Context (e.g.: course year, theoretical lessons; internship)**	Online during a semester
**Finding**	This study helped fill a gap in the literature by studying the effects of online mindfulness meditation to reduce stressors in fourth-year nursing students. The findings support nurse educators' use of an online mindfulness intervention to effectively reduce nursing students' anxiety and stress and improve their coping mechanisms.<br>Fifty-six students reported practicing mindfulness outside of the module, and 36 students answered question number two. Two common themes identified by the benefits students experienced from mindfulness activities: mindfulness is beneficial and time-worthy. Nursing students reported that online mindfulness activities were helpful in reducing stress, helping with relaxation, and decreasing stress levels. One student reported that the mindfulness grounding technique helped reduce stress and reduce the number of panic attacks experienced. Another student reported that the practice of mindfulness helped reduce stress in the clinical learning environment.
**Limits**	Small sample size. See Drop-out lack of control group. Internal validity: stress disappears regardless of the intervention for the maturity level of the students of the last year. External validity: prevalence of women and absence of control group. Confusing time on academic/clinical practices and periods. In addition, the validity of the CSI-SF emotional disengagement scale construct has been problematic and requires further testing.

**If unclear content, the author of the correspondence has been contacted**	May 23, 2024
**He replied within the indicated time (one week)**	NO
**Reference** [50]	Hutchison et al., 2016
**Country**	U.K.
**Study Design**	QE
**Sample Size**<br><br>**Dropout score**	25 final-year nursing students with learning disabilities (SLD)<br>*Dropout rate* (20%)
**MBIs applied**	Mindfulness in Learning Disability Practice workshop.<br>*Description of the intervention.*<br>FIRST DAY<br>9:15-9:30. Opening of the workshop; Presentation of the teachers and explanation of the reason why the workshop takes place and what it will entail.<br>9:30-9:45. Establish ground rules. Participants agree on the basic rules of the Workshop.<br>9:45-10:30. Icebreaker: Icebreaker activity "getting to know you". Each student is lecturer, introduces himself and explains the origin of his name and then shares an important thing learned during nursing training.<br>11:00-11:30. Power Point presentation of the contents:<br>Introduction to mindfulness, what it is and where it originated, why it is relevant to nursing.<br>11:30-12:00. Group discussion on the topic of autopilot. Discussion about being fully present. Students discuss using autopilot. What are the dangers and risks.<br>12:00-12:20. Practice of mindful body scanning with relaxation CD support.<br>13:20-14:00. Exercise "respond consciously without judging". Or observe the present moment without judgment. Students divide into groups and discuss the absence of judgment. What are the advantages of not judging patients, families, colleagues; what are the negative aspects of being judged. Then, the students pass on the results to the group.<br>14:00-14:15. Practice of conscious breathing with CD support of relaxation.<br>14:15-14:45. Conscious teamwork exercise. PowerPoint presentation of what is meant by teamwork; why it is important in nursing; what is the meaning of conscious teamwork.<br>SECOND DAY.<br>09:15-9:30. General discussion. Opening of the workshop. Summary of the discussions of day one; summary of basic rules; open space for questions.<br>9:30-10:00. Icebreaker activity, "Getting to know you". Each personal group will have to tell two truths and a lie and the others will have to try to guess the lie.<br>10:00-10:15. Practice of mainful compassion with relaxation CDs.<br>Each student is given a blank postcard. On the one hand, they write down what they will do to remain compassionate towards themselves throughout their careers. On the other hand, they write down what they will do to show compassion for others. Each student passes their own postcard around the group so they can write something positive about

	each other. After seeing these comments, the cards are returned to the course managers who keep them until the last day of the students' training.<br>10:45-11:00. PowerPoint presentation on the meaning of compassion in nursing.<br>11:15- 12:00. Communication exercise.<br>How to Listen and Speak with Compassion, Kindness, and Mindfulness<br>The barriers and promoters of effective communication with people and families with learning difficulties. Discuss examples from practice.<br>12:00-12:15. Practice of mindful eating.<br>13:15-14:00. Life balancing practice.<br>Students will complete the exercise "The Wheel of Life" The teacher then discusses the importance of maintaining a balanced life.<br>Mindful breathing practice with relaxation CD.<br>14:00-14:15. Open meditation practice with relaxation CD.<br>14:15-14:45. Closing from the workshop<br>Brief summary of what was covered today<br>Opportunity for students to ask questions<br>Students must fill out the evaluation form ok
**MBIs duration**	Nine hours in two days
**Mode of Delivery**	In presence
**Outcome(s)**<br><br>**Variable(s)**	- Reduction of provocative behaviours<br>- Increased level of calm<br>- Increased DM level
**Outcome(s) Measures**	Feedback was assessed using a combination of open-ended questions and a Likert scale. This aimed to measure changes in students' knowledge of mindfulness, as well as their experience
**Any adverse events related to MBIs interventions**	"College nursing programs need to consider how the application of mindfulness can create emotional dependency in students."
**Facilitator(s)**	psychologist
**Facilitator's competencies**	Lecturer trained at a seminar on leadership development *Decide, Commit, Proceed*. He tasked the *Positive Choices team* with organizing *Decide, Commit, Proceed*, a leadership course for third-year nursing students with learning disabilities.
**Context (e.g.: course year, theoretical lessons; internship)**	Ad hoc in-person workshop
**Finding**	Positive effects of the workshop support the idea of embedding mindfulness training in the nursing curriculum. Mindfulness training will be integrated into the University Programme for Learning Disabilities Nursing at Napier University Edinburgh. Services to consider the role of mindfulness in providing quality support for people with learning disabilities.
**Limits**	"Commitment on the part of the teachers: 3 for small groups. follow up on the use of MBIs techniques. The need for further research on the application of mindfulness and a comparison of the impact on stress reduction and practice. Undergraduate nursing programs need to consider how the application of mindfulness can create emotional dependence in students."

**If unclear content, the author of the correspondence has been contacted**	Not necessary
**He replied within the indicated time (one week)**	-
**Reference** [51]	Karaca & Şişman, 2019
**Country**	Turkey
**Study Design**	RCT
**Sample Size**<br><br>**Dropout score**	114 students who attended the 2nd academic year- Nursing Degree<br>(E: 42; C: 72)<br>*Dropout rate: 8,77%*<br>E: -3; C: -7
**MBIs applied**	E: MBSR<br>C: No intervention<br>Theoretical part: the autopilot<br>staying in the moment, pleasant and unpleasant events, bodily sensations, emotions or thoughts, acceptance and absence of judgment.<br>Experiential part:<br>- Body Scan<br>- Breath meditation<br>- Walking with mindfulness<br>- Mindful eating<br>- Meditation on self-love<br><br>C: No intervention<br><br>*Description of the intervention:*<br>Topics covered each week included the use of mindfulness to exit autopilot mode, body scan meditation, breath meditation, mindfulness during a routine activity (e.g., mindful walking, mindful eating), pleasant and unpleasant events, calendars, self-love meditation, bodily sensations, emotions or thoughts, thought monitoring, non-judgmental action, and acceptance.<br>Each session began by encouraging participants to focus their minds on mindful breathing, as the mind glides into the past or future, the present moment (approximately 5 minutes), followed by a task assessment (approximately 10 minutes), introduction and discussion of the main theme of the session (65 minutes), and assignment of tasks for the following week (5 minutes). Two sessions, involving mindful applications on walking, hearing and vision, were performed outside the classroom, in the schoolyard
**MBIs duration**	90-95 minutes twice a week for a total of 12 weeks
**Mode of Delivery**	In presence with a request for 10 minutes a day at home
**Outcome(s)**<br><br>**Variable(s)**	- Stress reduction<br>- Increase the level of self-confidence<br>- Increase the level of optimism

	- Reducing the impotent approach
**Outcome(s) Measures**	Nursing Education Stress Scale<br>Mindfulness Scale<br>Stress Management Styles Scale
**Any adverse events related to MBIs interventions**	NS
**Facilitator(s)**	Researcher responsible for managing the program was a cognitive and behavioral therapist
**Facilitator's competencies**	European Association for Behavioral and Cognitive Therapies Accreditation Certificate. The researcher was a psychiatric nurse with a Ph.D. and received mindfulness training and supervision as part of a 450-hour cognitive and behavioral therapist training. In addition, the researcher received an 8-week MBSR training course. The other researcher had also previously participated in an 8-week MBSR program.
**Context (e.g.: course year, theoretical lessons; internship)**	MBSR included in the Coping with stress course program during the autumn semester
**Finding**	A statistically significant difference was found in the mean posttest and mean follow-up scores obtained by the control group and the experimental group on the Nursing Education Stress Scale and its sub-dimensions. On the awareness scale, a statistically significant difference was found between the mean pretest and posttest scores obtained by the experiment and control groups.
**Limits**	Non-playability for other students. Only female nursing students. Results based on self-assessment
**If unclear content, the author of the correspondence has been contacted**	May 23, 2024
**He replied within the indicated time (one week)**	-
**Reference** [52]	Ksiksou et al., 2022
**Country**	Morocco
**Study Design**	QE pre/post-test
**Sample Size**<br><br>**Dropout score**	20 nursing students who attended 2nd and 3rd accademicYear-Nursing Degree<br><br>Dropout rate not specified
**MBIs applied**	MBSR: Sitting Meditation, Deep Breathing, Pre- and Post-Intervention Inquiry MBIs. They claim to have used the original protocol.
**MBIs duration**	Sessions of 2.30 hours per week with a minimum of 20 minutes of home practice for a total of eight weeks.
**Mode of Delivery**	In person and at home

**Outcome(s)**<br><br>**Variable(s)**	- Reduced stress level<br>- Increased level of Emotional Intelligence<br>- Explore the motivations for joining the course
**Outcome(s) Measures**	Perceived Stress Scale in Clinical Practicum (PSS-CP)<br>Emotional Intelligence Scale (EIS)
**Any adverse events related to MBIs interventions**	NS
**Facilitator(s)**	Psychiatrist Expert in MBSR
**Facilitator's competencies**	MBSR Specialization
**Context (e.g.: course year, theoretical lessons; internship)**	Extra-Curricular Course
**Finding**	the application of the MBSR protocol reduces stress levels and increases empathy levels in internship students. An inverse correlation between the two variables was also identified in pre- and post-treatment.
**Limits**	Small sample; Convenience Sample
**If unclear content, the author of the correspondence has been contacted**	May 23, 2024
**He replied within the indicated time (one week)**	NO
**Reference** [53]	Munif et al., 2024
**Country**	Indonesia
**Study Design**	QE
**Sample Size**<br><br>**Dropout score**	36 nursing students
**MBIs applied**	SOP protocol designed ad hoc based on Dwidiyanti's work: spiritual awareness vs mindfulness care. NS in detail<br>*Description of the hypothetical intervention (not complete and found from the article by the same author in 2019 which reproduces a very similar study).*<br>The intervention of this study is based on Islamic spiritual awareness and was administered in five sessions over five days.<br>In each session, participants carried out the following activities:<br>1- Arouse a desire to develop spiritual energy and the spirit of worship;<br>2- Introspectively analyze problems;<br>3- Develop full awareness of mistakes;<br>4- Feel the answer in your heart;

	5- Dzikr;<br>6- Acceptance;<br>7- Relaxation.<br><br>*Specification from the Treccani dictionary:*<br>*DHIKR - pronounced zikr in Eastern Arabic, Persian and Turkish dialects. An Arabic word, which in Muslim religious technical usage means to remember (God), whether this is done through meditation or internal prayer, or through uninterrupted repetition of the name of God or his epithets or pious formulas.*
**MBIs duration**	25 minutes for five days at home: morning, afternoon and evening.
**Mode of Delivery**	In presence with home activities
**Outcome(s)**<br><br>**Variable(s)**	Reduced stress levels
**Outcome(s) Measures**	Depression Anxiety and Stress Scale (DASS-42)
**Any adverse events related to MBIs interventions**	NS
**Facilitator(s)**	NS
**Facilitator's competencies**	NS
**Context (e.g.: course year, theoretical lessons; internship)**	NS
**Finding**	This research found that spiritual awareness and mindfulness-caregiving interventions can reduce pathological stress in nursing students. However, a difference was found, namely that spiritual mindfulness interventions were more able to reduce aggressive stress responses in nursing students. This study could open up the possibility of implementing the method of spiritual awareness in health care facilities or other institutions. However, this research needs to be re-tested on a sample group with different and more severe mental problems. Researchers have not found in previous research that spiritual mindfulness is effective for dealing with serious mental problems such as schizophrenia.
**Limits**	Convenience sample. Small sample size. Not general information to the nursing student population.
**If unclear content, the author of the correspondence has been contacted**	May 31, 2024
**He replied within the indicated time (one week)**	NO

**Reference** [54]	Öztürk, 2023
**Country**	Turkey
**Study Design**	RCT
**Sample Size**<br><br>**Dropout score**	64 nursing students who attended 1st academic year- Nursing Degree<br>Dropout rate: (8%)<br>E: 29-C:30
**MBIs applied**	E: Psychoeducation sessions based on an adapted MBSR program.<br>Participants were encouraged to read *Mindfulness for Beginners: Reclaiming the Present Moment-and Your Life* by John Kabat-Zinn and share their thoughts and experiences on mindfulness activities during the sessions.<br>The entire program was implemented online using the Zoom platform and was left to the discretion of the participants to share the video footage.<br><br>C: No treatment.<br><br><u>*Description of the intervention.*</u><br>**Session 1.**<br>Mindful breathing exercises (15 minutes) and sensory awareness exercises (taste-sight-hearing) for 45 minutes.<br>**Session 2.**<br>Body scan for 45 minutes: pay attention to the present moment and promote body awareness<br>Session 3.<br>Breathing and movement awareness: observing body movements and sensations in a curious and non-judgmental way, as if learning and discovering walking for the first time, and cultivating mindfulness during movement with mindful yoga postures.<br>**Session 4.**<br>Sitting meditation/pleasant and unpleasant moments: using awareness of sensations, while breathing as a basic focus and consciously placing your thoughts and emotional levels in the foreground or background of your attention while noticing other bodily sensations and environmental sounds.<br>**Session 5.**<br>Coping with difficult emotions/compassion: exploring and accepting emotions without judgment, feeling compassion and gratitude.<br>**Session 6.**<br>Silence: the practices made during the program are lived again, with the aim of increasing the participant's intuition; The main result of learning is that silence and calm are not the same thing.<br>**Session 7.**<br>Conscious communication: being able to listen to ourselves and others with a non-judgmental, accepting and compassionate attitude<br>**Session 8.**<br>Metaphorical approaches/mountain meditation.<br><br>At the end of each session, sharing the experience for 30 minutes. Mandate for home practices by the teacher. At the opening of each session, 15 minutes of sharing the practice diary.

**MBIs duration**	90 minutes twice a week at the front of 2.30 hours twice a week. Four-hour retreat and not eight hours.
**Mode of Delivery**	Online on Zoom platform
**Outcome(s)**<br><br>**Variable(s)**	- Implementation of psychological well-being<br>- Increased levels of emotional intelligence<br>- Reduced stress levels
**Outcome(s) Measures**	14 items-Perceived Stress Scale (PSS-14)<br>Psychological Well-Being Scale (PWB)<br>Revised Schutte Emotional Intelligence Scale (SEIS)
**Any adverse events related to MBIs interventions**	NS
**Facilitator(s)**	The researcher
**Facilitator's competencies**	Certified Cognitive-Behavioral Therapist, Mindfulness and Acceptance Engagement Professional
**Context (e.g.: course year, theoretical lessons; internship)**	Extra-Curricular Course
**Finding**	In the pretest/post-test comparison of PSS scores within the groups, a significant reduction in perceived stress levels was observed in the intervention group ($Z = -4{,}630$; $P < 0.001$), while there was a significant increase in perceived stress levels in the control group ($Z = -3.090$; $P < 0.002$). The opposite pattern was observed for PWBS and RSEIS. The intervention group showed significant increases in scores for psychological well-being ($Z = -4.376$, $P < 0.001$) and emotional intelligence ($Z = -3.920$, $P < 0.001$), while these scores decreased significantly in the control group ($Z = -2.135$, $P = 0.033$ and $Z = -3.175$, $P = 0.001$, respectively)
**Limits**	Small sample size. Sample of convenience. General information on the nursing student population (prevalence of women). Self-administered reports.
**If unclear content, the author of the correspondence has been contacted**	May 23, 2024
**He replied within the indicated time (one week)**	NO
**Reference** [55]	Quatraro, Gallegos & Walters, 2024
**Country**	USA
**Study Design**	QE pre/post-test
**Sample Size**<br><br>**Dropout score**	35 nursing students<br><br>Dropout rate: 25,71%
**MBIs applied**	Smiling Mind: Library of pre-recorded audio files.

	Mindfulness exercises range from 1 to 45 minutes, and the user decides on the content and duration that best suits their needs. To help participants get started, they were advised to use the Mindfulness Foundations program within the application. Previous research on meditation has shown that 5-10 minutes of meditation can affect stress, anxiety, and mood (Berghoff et al., 2017; Mantzios & Giannou 2018). For this reason, participants were asked to use the app for at least eight to ten minutes a day, four days a week, for six weeks
**MBIs duration**	6 weeks:<br><br>The students used the app for 3-4 days a week for 5 – 7 minutes
**Mode of Delivery**	App on your phone: Smiling Mind
**Outcome(s)**<br><br>**Variable(s)**	Stress, Depression, Self-Compassion
**Outcome(s) Measures**	Perceived Stress Scale (PSS)<br>Beck's Depression Inventory (BDI)<br>Self-Compassion Scale (SCS)
**Any adverse events related to MBIs interventions**	NS
**Facilitator(s)**	app
**Facilitator's competencies**	NS
**Context (e.g.: course year, theoretical lessons; internship)**	3rd and 4th year students
**Finding**	statistically significant reduction in stress and depression and an increase in total self-compassion
**Limits**	Convenience Sample<br><br>Absence of a control group<br><br>Monocentricity
**If unclear content, the author of the correspondence has been contacted**	-
**He replied within the indicated time (one week)**	-
**Reference** [56]	Ratanasiripong et al., 2015
**Country**	Thailand
**Study Design**	RCT

**Sample Size**<br><br>**Dropout score**	89 nursing students who attended the 2nd academic year- Nursing Degree. They were divided into three groups:<br>E-1: 29 students<br>E-2: 29 students<br>C: 29 students
**MBIs applied**	**E-1:** Biofeedback<br>**E-2**: Vipassana Meditation<br>**C: No** intervention
**MBIs duration**	Three times a day for four weeks.<br>NS Time for Each Session
**Mode of Delivery**	In presence
**Outcome(s)**<br><br>**Variable(s)**	- Reduced stress level<br>- Reduced anxiety level<br>- Improved academic performance
**Outcome(s) Measures**	Perceived Stress Scale (PSS)<br>State-Trait Anxiety Inventory (STAI)
**Any adverse events related to MBIs interventions**	NS
**Facilitator(s)**	Meditation instructor
**Facilitator's competencies**	Trainer and instructor NS the path
**Context (e.g.: course year, theoretical lessons; internship)**	Before the clinical internship
**Finding**	The results indicated that biofeedback significantly reduced anxiety and maintained stress levels in nursing students.<br>Mindfulness meditation similarly reduced anxiety levels, while also significantly lowering stress levels.<br>The biofeedback group showed a significant reduction in anxiety levels among the three groups at post-intervention.
**Limits**	Low generalization of results in the nursing population by female-only sample
**If unclear content, the author of the correspondence has been contacted**	May 23, 2024
**He replied within the indicated time (one week)**	NO
**Reference** [57]	Sari Ozturk & Kilicarslan Toruner, 2022
**Country**	Turkey
**Study Design**	RCT

**Sample Size**<br><br>**Dropout score**	180 nursing students (E: 90; C:90)<br>Dropout rate: 5,55%<br>E: -6; C: - 4
**MBIs applied**	mindfulness-based mandalas (breathing, guided visualizations, mandala coloring)
**MBIs duration**	E: Three one-hour meetings for three weeks, one meeting per week.<br>C: No treatment
**Mode of Delivery**	Online on Zoom platform
**Outcome(s)**<br><br>**Variable(s)**	- Reduced anxiety level<br>- Increased spiritual well-being<br>- Emotional management and comfort
**Outcome(s) Measures**	Spielberg State-Trait Anxiety Inventory (STAI)<br>Spiritual Well-Being Scale (SWBS)<br>Positive and Negative Experiences Scale (SPANE)
**Any adverse events related to MBIs interventions**	NS
**Facilitator(s)**	Researcher
**Facilitator's competencies**	Mindfulness-based mandala drawing certificate and a therapeutic art life coach certificate prior to study
**Context (e.g.: course year, theoretical lessons; internship)**	During the clinical internship in the COVID period
**Finding**	Statistically significant improvement of the investigated outcomes. Mindfulness-based mandala activity was found to be effective in reducing anxiety and improving spirituality, emotion management, and comfort in nursing students with high levels of anxiety under pandemic conditions.
**Limits**	Very short intervention period that does not allow to generalize the results
**If unclear content, the author of the correspondence has been contacted**	May 23, 2024
**He replied within the indicated time (one week)**	NO
**Reference** [58]	Scheick, 2011
**Country**	USA
**Study Design**	Mixed method
**Sample Size**<br><br>**Dropout score**	30 nursing students who attended the final academic year- Nursing Degree<br>Drop-out rate: - 8 (27%)<br>E: 15- C: 7
**MBIs applied**	E: STEDFAST S-AM.<br>Building awareness in the nurse-client relationship through:

	This protocol consists of questions to evaluate each individual item, based on the answer, it will be necessary to perform conscious breathing exercises.<br><br>C: No treatment
**MBIs duration**	Three minutes of breathing before the meetings.<br>NS The number of meetings
**Mode of Delivery**	In presence
**Outcome(s)**<br><br>**Variable(s)**	- Increase the level of self-control<br>- Increasing the level of self-awareness
**Outcome(s) Measures**	Element S: Self-concept examination by Schutz:<br>Self-concept assessment. This tool measures the self-concept components of liveliness (similar to mindful attention), self-control (similar to conscious stress control), and self-awareness (similar to conscious reflective attention to oneself and others).
**Any adverse events related to MBIs interventions**	NS
**Facilitator(s)**	NS
**Facilitator's competencies**	NS
**Context (e.g.: course year, theoretical lessons; internship)**	Two semesters for community psychiatric and mental health nursing
**Finding**	QN: showed not only statistically significant changes in self-awareness, which had been determined in Scheick's (2004) previous study, but also statistically significant changes in vibrating liveliness and self-control.<br>QL: Students who implement the model report an increase in self-awareness, especially since they readily use the mnemonic portion of STEDFAST self-awareness in clinical encounters
**Limits**	Very short intervention period that does not allow to generalize the results
**If unclear content, the author of the correspondence has been contacted**	May 24, 2024
**He replied within the indicated time (one week)**	NO
**Reference** [59]	Spadaro & Hunke, 2016
**Country**	USA
**Study Design**	Quasi-experimental pre post test
**Sample Size**<br><br>**Dropout score**	27 nursing students<br><br>Dropout rate: 3,70%

**MBIs applied**	MBSR: Eating Meditation, Body Scanning, Mindful Breathing, Sitting Meditation, Walking Meditation, Mindful Movement Through Hatha Yoga, Guided Meditations, and Loving-Kindness Meditation.<br>Descriptions for each of the eight components (one component per week), detailed mindfulness exercises with video demonstration (including yoga), and narration were uploaded to the university's learning management system. During the eight weeks of online intervention, participants were encouraged to practice the components during the week and write about their experiences via an online discussion forum.<br>After the assessments were completed, participants were sent a copy of Kabat-Zinn's book, *Wherever You Go There You* Are and his CD, *Mindfulness for Beginners* to reinforce the continuation of mindfulness meditation practices.
**MBIs duration**	At least one day a week for eight weeks.<br>NS the time for each session
**Mode of Delivery**	Online in asynchronous mode
**Outcome(s)**<br><br>**Variable(s)**	- Reduced stress level<br>- Reduction in the level of depression<br>- Increased level of attention
**Outcome(s) Measures**	Perceived Stress Scale (PSS)<br>Hospital Anxiety and Depression Scale (HADS)<br>Attention Network Test (ANT)
**Any adverse events related to MBIs interventions**	NS
**Facilitator(s)**	NS
**Facilitator's competencies**	NS
**Context (e.g.: course year, theoretical lessons; internship)**	During a semester that was not the last
**Finding**	stress was significantly reduced (F (2.24) =4.163, p=.019). A decreasing trend for anxiety was noted with a significant difference between time points (F (1,23) =6,889, p=.015) when the frequency of practice was weekly or daily. Cognition: The ability to shift attention, attention selection, concentration, and accuracy are improved.
**Limits**	Small sample size
**If unclear content, the author of the correspondence has been contacted**	May 24, 2024
**He replied within the indicated time (one week)**	NO
**Reference** [60]	Tarhan & Elibol, 2023
**Country**	Turkey

**Study Design**	QE pre/post-test
**Sample Size**<br><br>**Dropout score**	78 nursing students<br>E: 36-C: 42 (Dropout rate: not specified)
**MBIs applied**	E. Short MBSR: mindful breathing, body scanning, letting go, taking a mental vacation, mindful walking and noticing yourself.<br><br>C: Traditional teaching on clinical errors
**MBIs duration**	1 hour per week for four weeks
**Mode of Delivery**	In presence
**Outcome(s)**<br><br>**Variable(s)**	(a) attitudes of medical error<br>(b) the number of medical errors and risks in a simulation environment<br>(c) self-confidence
**Outcome(s) Measures**	The Attitudes to Medical Error scales<br>Simulation Design Scale<br>Student Satisfaction and Self-Fidence Scale in Learning
**Any adverse events related to MBIs interventions**	NS
**Facilitator(s)**	Second author
**Facilitator's competencies**	Trained in MBSR
**Context (e.g.: course year, theoretical lessons; internship)**	Ad hoc workshop
**Finding**	There was a statistically significant improvement in the intervention group between the pre-test and post-test for medical error attitudes ($p < 0.001$) and the number of medical errors and risks in a simulation environment ($p < 0.001$). There was no statistical difference in the intervention and control groups for self-confidence and satisfaction ($p > 0.05$). A short MBSR program significantly strengthened nursing students' awareness of medical errors and risks. However, the program does not cause any difference in satisfaction and self-confidence in students' learning levels.
**Limits**	Small sample size. Sample of convenience on voluntary choice. No blindness
**If unclear content, the author of the correspondence has been contacted**	May 24, 2024
**He replied within the indicated time (one week)**	NO
**Reference** [61]	Torné-Ruiz et al., 2023
**Country**	Spain
**Study Design**	QE pre/post-test

**Sample Size**<br><br>**Dropout score**	52 nursing students<br>Dropout rate: 19,23%<br>E: -5; C: -5
**MBIs applied**	10-daymindfulness.<br>It consisted of an audiovisual recording with which the participants practiced relaxation and conscious breathing exercises in a 25-minute session.
**MBIs duration**	25 minutes in 10 days
**Mode of Delivery**	Online
**Outcome(s)**<br><br>**Variable(s)**	- Reduced stress level<br>- Reduced anxiety level
**Outcome(s) Measures**	Self-administered Analogue Stress Scale<br>State-Trait Anxiety Inventory<br>Five Facet Mindfulness Questionnaire
**Any adverse events related to MBIs interventions**	NS
**Facilitator(s)**	Mindfulness instructor
**Facilitator's competencies**	Awareness expert with experience in conducting courses and training
**Context (e.g.: course year, theoretical lessons; internship)**	Before the clinical simulations of two courses (Adult Nursing Care I and General Pathophysiology II)
**Finding**	statistically significantly lower in the experimental group (diastolic blood pressure p = .032 and heart rate p = .048). Stress (p = .029) and anxiety (p = .016) levels were also better managed.
**Limits**	Reduced sample size and therefore no possibility of generalizing the results.
**If unclear content, the author of the correspondence has been contacted**	May 24, 2024
**He replied within the indicated time (one week)**	NO
**Reference** [62]	Yüksel & Bahadır Yılmaz, 2020
**Country**	Turkey
**Study Design**	QE pre/post-test
**Sample Size**<br><br>**Dropout score**	82 nursing students who attended the 2nd academic year- Nursing Degree<br>Dropout rate not specified
**MBIs applied**	Conscious breathing exercises, awareness of daily activities. breathing exercises, body scan meditation, sound and thought meditation.

	Cognitive therapy program: The cognitive therapy program based on group mindfulness included stress, stress management, stress coping methods, mindfulness, mindfulness-based techniques, and dysfunctional beliefs such as cognitive distortions and automatic thoughts.<br>*Description of the intervention:*<br><br>**Session 1**: Meeting, information provided on how the group will be conducted, expressing expectations and giving information on the concept of awareness, administering pre-tests.<br><br>**Session 2:** Understand the relationship between the cognitive model and cognitive distortions, emotion, thinking and behavior and achieve the skills to recognize cognitive errors.<br><br>**Session 3**: Mindfulness, being here and now, living in the moment, mindful breathing exercises, mindfulness of daily activities.<br><br>**Session 4:** Automatic thoughts, questioning your automatic thoughts, asking the right questions to reach a correct evaluation, automatic pilot recognition.<br><br>**Session 5:** Stress Coping Methods: What is Stress? What are the effects of stress? How can we cope with stress?<br><br>**Session 6**: Methods for coping with stress: Explaining the concept of non-functional thinking during stressful experiences, dealing with non-functional negative automatic thoughts, recognizing evidence that supports and does not support negative automatic thoughts.<br><br>**Session 7**: Dealing with difficulties, accepting painful emotions, becoming mindful during breathing exercises, body scan meditation, sound and thought meditation.<br><br>**Session 8**: Evaluation, synthesis, obtaining feedback from group members and administering post-tests.
**MBIs duration**	Two hours a week for eight weeks
**Mode of Delivery**	In presence
**Outcome(s)**<br><br>**Variable(s)**	- Reduced stress level<br>- Reduced anxiety level<br>- Increased level of awareness
**Outcome(s) Measures**	Mindful Attention Awareness Scale (MAAS)<br>Depression, Anxiety and Stress Scale (DASS)
**Any adverse events related to MBIs interventions**	NS
**Facilitator(s)**	The first researcher
**Facilitator's competencies**	She had received training and supervision on cognitive and behavioral therapy and mindfulness-based stress reduction.

**Context (e.g.: course year, theoretical lessons; internship)**	Extra-Curricular Course
**Finding**	Mean scores obtained in pre-test, post-test, and follow-up measurements, mean MAAS scores increased (p = .000), and stress scores decreased significantly in the experimental group (p = .004).
**Limits**	However, the program did not cause any change in students' anxiety and depression levels. Non-randomization of the sample
**If unclear content, the author of the correspondence has been contacted**	May 24, 2024
**He replied within the indicated time (one week)**	NO
**Reference** [63]	Arthur, 2022
**Country**	USA.
**Study Design**	RCT (D/T)
**Sample Size**<br><br>**Dropout score**	57-60 Unclear
**MBIs applied**	E: A Mindfulness Record on Mindful Breathing<br><br>C: a relaxing music recording to make a mental excursus on your day<br>*Description of the interventions.*<br><br>The experimental group listened to a brief conscious breathing activity of about 3 minutes. The control group listened to a short, 3-minute, relaxing, reflection-based recording.<br><br>Both recordings opened with the statement: "We will start this session with a short mindfulness exercise", then, depending on the group/recording to which the participants were assigned, the recording differed.<br>The experimental group listened to a mindfulness recording that focused on mindful breathing, while the control group listened to a recording that prompted them to take a mental tour of their day while listening to some relaxing classical music.<br>Both recordings were almost the same length, with the mindful breathing recording lasting about 10 seconds longer.
**MBIs duration**	3 minutes before theory classes until exam day
**Mode of Delivery**	in presence
**Outcome(s)**<br><br>**Variable(s)**	- Stress reduction<br>- Increased DM levels

	- Improved academic performance
**Outcome(s) Measures**	Mindfulness Attention Awareness Scale (MAAS); Perceived Stress Scale (PSS)
**Any adverse events related to MBIs interventions**	NS
**Facilitator(s)**	researcher unrelated to the degree course
**Facilitator's competencies**	NS
**Context (e.g.: course year, theoretical lessons; internship)**	before the theoretical lessons of the pediatric nursing course during the autumn session
**Finding**	There appears to be no statistically significant effect of the treatment group on test scores, MAAS (end of semester) or PSS (end of semester). There was no increase in awareness, decrease in perceived stress, or academic improvement with this cohort of nursing students.
**Limits**	Convenience sample. Methodological error in the research (due to the error in the collection of email addresses in the pre-intervention survey, the researcher was unable to assess the correlations of individual participants between MAAS and PSS scores or between MAAS, PSS and exam scores).
**If unclear content, the author of the correspondence has been contacted**	NP
**He replied within the indicated time (one week)**	-
**Reference** [64]	Baich, 2022
**Country**	USA
**Study Design**	QE (D/T)
**Sample Size**<br><br>**Dropout score**	34 nursing students<br>Dropout rate<br>-9 (26%)
**MBIs applied**	mindfulness guide with smartphone App "Smiling mind" which contains 18 different programs and 208 different mindfulness sessions.<br><br>Students have completed:<br>- three sessions in "Introduction to Mindfulness";<br>- two sessions in "Mindfulness: The Fundamentals";<br>- four sessions in "studies sessions";

	- four sessions of "Stress Management".<br>Online to be able to do it outside of academic commitments
**MBIs duration**	12 days in 5 weeks (for a total of 75 min with an average per session of 6.32 min)
**Mode of Delivery**	online APP<br><br>https://www.smilingmind.com.au
**Outcome(s)**<br><br>**Variable(s)**	Reduction of anxiety (resulting from academic demands)
**Outcome(s) Measures**	Westside Test Anxiety Scale (WTAS)
**Any adverse events related to MBIs interventions**	NS
**Facilitator(s)**	free online guide
**Facilitator's competencies**	NS
**Context (e.g.: course year, theoretical lessons; internship)**	Online, before theoretical lessons
**Finding**	a statistically significant impact ($p < 0.001$) on the reduction of WTAS scores on senior nursing students, which supports the importance of this project. In addition, the results of this project can promote faculty to direct students to practice mindfulness to reduce stress and anxiety.
**Limits**	Convenience sample. Low sample size.
**If unclear content, the author of the correspondence has been contacted**	NP
**He replied within the indicated time (one week)**	-
**Reference** [65]	Bonfe, 2024
**Country**	USA
**Study Design**	MM (D/T)
**Sample Size**<br><br>**Dropout score**	24 nursing students who attended the 1st academic year<br>*Dropout rate:* Not specified

**MBIs applied**	A Mindfulness Meditation exercise (breathing and attention) on YouTube<br>https://www.youtube.com/watch?v=3yxgFAW7wTc
**MBIs duration**	5 minutes before the exam for 6 exams
**Mode of Delivery**	Online before the exam through the semester
**Outcome(s)**<br><br>**Variable(s)**	- Reduced anxiety<br>- Reduced stress<br>- Reduced depression<br>- Student perception on awareness and resilience
**Outcome(s) Measures**	Depression Anxiety and Stress Scale (DASS-21)
**Any adverse events related to MBIs interventions**	NS
**Facilitator(s)**	The researcher led the project and the MM moments
**Facilitator's competencies**	The researcher who implemented the project. He completed a 20-minute basic training in mindfulness. The training included motivation and guide to exercise of awareness.
**Context (e.g.: course year, theoretical lessons; internship)**	Introduced during the pharmacology course.<br>Before the exams of the whole semester
**Finding**	QN: Statistically insignificant reduction in anxiety and stress<br>QL: increased sense of calm, relaxation, centering, focusing
**Limits**	Convenience sampling. Low sample size.
**If unclear content, the author of the correspondence has been contacted**	NP
**He replied within the indicated time (one week)**	-
**Reference** [66]	Davis, 2020
**Country**	USA
**Study Design**	RCT/(DT)
**Sample Size**<br><br>**Dropout score**	120<br>12 clusters with 10 participants per cluster. Dropout rate<br>Not specified
**MBIs applied**	E: Koru Mindfulness meditation<br>C: wait list<br><br>Koru Mindfulness Meditation Intervention<br>**Session 1**<br>• Opening meditation

	• Belly breathing<br>• Dynamic breathing<br>• Guided meditation: body scan<br>**Session 2**<br>• Opening meditation<br>• Walking meditation<br>• Guided meditation: Gathas<br>**Session 3**<br>• Opening of mediation<br>• Guided imagery<br>• Guided meditation: labeling thoughts<br>**Session 4**<br>• Opening meditation<br>• Food meditation<br>• Guided meditation: labeling feelings
**MBIs duration**	75 minutes per session; one session per week for four weeks
**Mode of Delivery**	Online on Zoom platform (Live Webinar)
**Outcome(s)**<br><br>**Variable(s)**	- Stress reduction<br>- Reduction of depression<br>- Assess the feasibility of the intervention
**Outcome(s) Measures**	Stress and Depression subscales of the Depression Anxiety Stress Scales (DASS-21)<br>Ego Resilience (ER) Scale<br>Brief COPE instrument<br>Interpersonal Support Evaluation List (ISEL-12)<br>Positive Emotion subscale and the Negative Emotion subscale of the Scale of Positive and Negative Experience (SPANE)<br>Perceived Stress Scale (PSS)<br>Interpersonal Relationships subscale of the Health-Promoting Lifestyle Profile II (HPLP II)
**Any adverse events related to MBIs interventions**	NS
**Facilitator(s)**	Psychiatrist and Koru Mm instructor
**Facilitator's competencies**	Mm Expert Certificate
**Context (e.g.: course year, theoretical lessons; internship)**	Extra-Curricular Course
**Finding**	The variance of stress ($p < 0.001$) is about 64% of the variance of depressive symptoms ($p < 0.001$). Negative and coping emotions were significantly correlated with stress ($p = 0.045$, $p = 0.003$, respectively). Coping, personal resources, positive emotions, and negative emotions were significantly correlated with depressive symptoms ($p = 0.009$, $p = 0.037$, $p = 0.009$, $p < 0.001$, respectively).<br>While web-based mindfulness meditation has been effective in reducing stress and depression, symptoms in this sample of nursing students, larger studies using more rigorous study designs are needed to improve the reliability and generability of these

	findings. Symptoms In this sample of nursing students, larger studies using more rigorous study designs are needed to improve reliability and generability<br>of these results. Symptoms In this sample of nursing students, larger studies using more rigorous study designs are needed to improve the reliability and generability of these findings. Symptoms In this sample of nursing students, larger studies using more rigorous study designs are needed to improve the reliability and generability of these findings.
**Limits**	The online mode did not allow for the dosage and personalization of interventions, especially those at home.<br>The lack of post-surgery confrontation even though there was an open space to write.
**If unclear content, the author of the correspondence has been contacted**	NP
**He replied within the indicated time (one week)**	-
**Reference** [67]	Dodson, 2021
**Country**	USA
**Study Design**	QE (D/T)
**Sample Size**<br><br>**Dropout score**	39 nursing students in the first semester of Nursing Degree<br><br>Dropout rate: not specified
**MBIs applied**	MBSR: Burdick's Workbook.<br>Practices:<br>- Mindful breathing<br>- Guided Imagination<br>- Body Scan
**MBIs duration**	Every week in class before class for 15-20 minutes before the placement test
**Mode of Delivery**	In presence
**Outcome(s)**<br><br>**Variable(s)**	- Reduced anxiety<br>- Assessment of concerns and emotionality
**Outcome(s) Measures**	Test Anxiety Inventory (TAI) contains subscale for emotionality and worries
**Any adverse events related to MBIs interventions**	NS
**Facilitator(s)**	Expert-trained teachers
**Facilitator's competencies**	Three weeks in the first half according to the Burdick protocol (2013)
**Context (e.g.: course year, theoretical lessons; internship)**	Before the classroom lesson until the final test (1st semester)

**Finding**	Within the study, statistical analysis of pre- and post-intervention data demonstrated a decrease in overall anxiety, worry, and emotionality of the test. Students also reported mindfulness interventions to faculty leaders who had practiced the mindfulness strategies learned in this study during clinical experiences in hospital settings and during simulation activities. Although not statistically relevant, participants' informal feedback supports the transfer of mindfulness to other contexts and may indicate that nursing students might use mindfulness techniques in their post-educational professional experiences
**Limits**	internal and external validity of the study and prediction of poor adherence in the future
**If unclear content, the author of the correspondence has been contacted**	May 25, 2024
**He replied within the indicated time (one week)**	NO
**Reference** [68]	Foster, 2017
**Country**	USA
**Study Design**	RCT/(DT)
**Sample Size**<br><br>**Dropout score**	12 Nursing Students who attended the 2nd academic year- Nursing Degree<br>(E: 6; C: 6)
**MBIs applied**	E: MBIs intervention adapted from "The Effectiveness of a Stress Coping Program based on Mindfulness Meditation on the Stress, Anxiety, and Depression Experienced by Nursing Students in Korea,"<br>by Y. S. Kang, S. Y. Choi, and E. Ryu, 2009, Nurse Education Today, 29, p. 540."<br>C: self-managed study with supervision.<br><br>**Purpose** — **Intervention Strategies/Techniques**<br><br>**Session 1**<br><br>Creating Reports — Introductory statements and course objective<br>Instruction — What is mindfulness<br>Practice — Sitting meditation; Walking meditation<br>Mindfulness exercises — Yoga<br><br>**Session 2**<br><br>Group discussion — Review of the last session and reflection on Application during the week.<br>Instruction — Spreading self-talk<br>Practice — My good stitch scan

		Breathing meditation
	Mindfulness exercises	Yoga
		**Session 3**
	Group discussion	Review of the last session and reflection on Application during the week.
	Instruction	Body Scanning Techniques
	Practice	Body Scan Meditation
		Sitting meditation
	Mindfulness exercises	Yoga
		**Session 4**
	Group discussion	Review of the last session and reflection on Application during the week.
	Instruction	Promote a sense of gratitude
	Practice	Thank you scan
		Breathing meditation
	Mindfulness exercises	Yoga
		**Session 5**
	Group discussion	Review of the last session and reflection on Application during the week.
	Instruction	Stretching
	Practice	Music, meditation and happy memories
		Walking meditation
	Mindfulness exercises	Yoga
		**Session 6**
	Group discussion	Review of the last session and reflection on Application during the week.
	Practice	Stretching
		Body Scan Meditation
		Walking meditation
	Mindfulness exercises	Yoga

	**Session 7**<br>Group discussion — Review of the last session and reflection on Application during the week.<br>Practice — Breathing meditation<br>Sitting meditation<br>Loving-kindness meditation<br>Mindfulness exercises — Yoga<br>**Session 8**<br>Group discussion — Review of the last session and reflection on Application during the week.<br>Practice — Thank you Scan<br>Body Scan<br>Walking meditation<br>Culminating experience — New beginning
**MBIs duration**	1 hour session for 8 sessions
**Mode of Delivery**	in person and at home
**Outcome(s)**<br>**Variable(s)**	- Stress reduction<br>- Reduced anxiety<br>- Improve learning and time management strategies
**Outcome(s) Measures**	Derogatis Stress Profile (DSP)<br>Learning and Study Strategies Inventory (LASSI)
**Any adverse events related to MBIs interventions**	NS
**Facilitator(s)**	Mindfulness Facilitator
**Facilitator's competencies**	Formal training in mindfulness-based stress reduction (MBSR) from the Oasis Institute (University of Massachusetts, 2014), which is part of the Center for Mindfulness in Medicine, Health Care, and Society<br>(CMMHCS) at the University of Massachusetts (2014), which offers MBSR certification training in over 200 medical centers, hospitals, and clinics worldwide (CMMHCS, 2014).
**Context (e.g.: course year, theoretical lessons; internship)**	Ad hoc workshop before the internship experience
**Finding**	From before to after the test, the experimental group showed greater stress level reductions at size, domain, and global portions according to the DSP.

	According to the LASSI, from pre to post-test both the control and experimental groups have strengthened learning and study strategies; However, the experimental group exhibited a greater level of strengthening."
**Limits**	Small sample size. Convenience sample. Students who did not reside in the college and therefore have higher stress levels.
**If unclear content, the author of the correspondence has been contacted**	NP
**He replied within the indicated time (one week)**	-
**Reference** [69]	Heinrich, 2022
**Country**	USA
**Study Design**	RCT/(DT)
**Sample Size**<br><br>**Dropout score**	195 nursing students (E: 99; C: 96)<br>*Dropout rate: 25,64%*<br>E: -17; C: -33
**MBIs applied**	E: Listening to 10 minutes of meditation recording<br>C: Listening to 10 minutes recording on nursing notions<br>*Description of interventions:*<br>Weekly scripts for a 10-minute mindfulness meditation session<br>Week 1 - introduction<br>Written instructions provided in Canvas: Take this time for yourself. Follow these tips to improve your meditation experience<br>1. It is suggested to practice this meditation at the same time every day<br>2. Find a quiet and comfortable place to sit<br>3. Silence cell phones or any distractions<br>4. Be patient with yourself. It takes time to build a meditation practice.<br><br>Week 2-stress<br>Written instructions provided in Canvas: Take this time for yourself. Follow these tips to improve your meditation experience<br>1. It is recommended to practice this meditation at the same time every day<br>2. Find a quiet and comfortable place to sit<br>3. Silence cell phones or any distractions<br>4. Be patient with yourself. It takes time to build a meditation practice.<br>Week 3 - self-compassion<br>Written instructions provided in Canvas: Take this time for yourself. Follow these tips to improve your meditation experience<br>1. It is recommended to practice this meditation at the same time every day<br>2. Find a quiet and comfortable place to sit<br>3. Silence cell phones or any distractions<br>4. Be patient with yourself. It takes time to build a meditation practice.

	Week 4 - gratitude<br>Written instructions provided in Canvas: Take this time for yourself. Follow these tips to improve your meditation experience<br>1. It is recommended to practice this meditation at the same time every day<br>2. Find a quiet and comfortable place to sit<br>3. Silence cell phones or any distractions<br>4. Be patient with yourself. It takes time to build a meditation practice.<br><br>The text of the recording "Appendix H" is present
**MBIs duration**	Three days a week for 4 weeks
**Mode of Delivery**	Audio resources
**Outcome(s)**<br><br>**Variable(s)**	Perceived level of stress, anxiety, self-compassion, mindfulness
**Outcome(s) Measures**	Perceived Stress Scale (PSS)<br>Generalized Anxiety Disorder sub-scale (GAD-7)<br>Self-Compassion Scale (SCS)<br><br>Mindfulness Attention Awareness Scale (MAAS)
**Any adverse events related to MBIs interventions**	NS
**Facilitator(s)**	Recorded Meditation
**Facilitator's competencies**	NS
**Context (e.g.: course year, theoretical lessons; internship)**	NS
**Finding**	Online mindfulness meditation can help nursing students reduce stress and anxiety, while also increasing mindfulness and self-compassion. he p-values for these findings were less than 0.05, indicating statistical significance.
**Limits**	Attrition rate; self-reported data.
**If unclear content, the author of the correspondence has been contacted**	NN
**He replied within the indicated time (one week)**	NN
**Reference** [70]	Kinney, 2022
**Country**	USA

**Study Design**	QE pre/post-test test ((D/T)
**Sample Size**<br><br>**Dropout score**	78 Nursing Students who attended 2nd Accademic Year – Nursing Degree<br>(E: 50-C:28)<br>*Dropout rate: 59,60%*<br>E: -59; C: -62
**MBIs applied**	E: Guided breathing (abbreviated MBIs). Three breathing sequences with different pauses between inhalation and exhalation (Dr. Andrew Weil, breathing 4-7-8)<br>https://www.youtube.com/watch?v=YRPh_GaiL8s<br><br>C: NS<br>*Description of the intervention*:<br>Dr. Andrew Weil's video on the health benefits of 4-7-8 breathing (refer to video) was presented; The demonstration was about eight minutes. The experimental group completed four sets of 4-7-8 breathing exercises at the beginning and end of each classroom teaching session. The set of four lasted less than 1 minute. In the last three weeks of the session, the experimental group completed eight sets of breathing 4-7-8 exercises at the beginning and end of each classroom teaching session. The set of 8 took less than 1.5 minutes.
**MBIs duration**	One minute and 30 seconds
**Mode of Delivery**	Online
**Outcome(s)**<br><br>**Variable(s)**	Increase the level of empathy
**Outcome(s) Measures**	Jefferson Scale of Empathy for Health Professions Students JSE-HPS
**Any adverse events related to MBIs interventions**	NS
**Facilitator(s)**	NS.<br>The host of the video is Dr. Andrew Weil.
**Facilitator's competencies**	NS.<br>Dr. Andrew Weil is a holistic physician
**Context (e.g.: course year, theoretical lessons; internship)**	Before and after the theoretical lessons
**Finding**	The results did not show statistical significance
**Limits**	Convenience sample. No possibility of generalizing the results but only by study population. Virtual environment due to covid
**If unclear content, the author of the correspondence has been contacted**	NP

**He replied within the indicated time (one week)**	-
**Reference** [71]	Klich, 2019
**Country**	USA
**Study Design**	QE pre/post-test test (D/T); case report
**Sample Size**<br><br>**Dropout score**	15 nursing students<br><br>Dropout score: 40%
**MBIs applied**	E: Mindfulness Meditation according to the book and the CD by Sharon Salzberg: *Salzberg, S. (2010). Real Happiness: The Power of Meditation: A 28-Day Program (Pap/Com edition). New York: Workman Publishing Company.*<br><br>*Description of the intervention:*<br>Theoretical practice: read 30 minutes of an excerpt from the book (every week). Delivery of the book and recordings.<br>Experiential practice:<br>Week 1: Concentration – Breathing Meditation<br>Week 2: Body Mindfulness – Walking Meditation<br>Week 3: Mindfulness of Emotions – Meditation on Emotions<br>Week 4: Cultivating Compassion – Meditation on Loving-Kindness
**MBIs duration**	15 minutes a day of meditation and 30 minutes a week of reading for 4 weeks
**Mode of Delivery**	At home with supplementary materials for practice (Book and the CD by Sharon Salzberg)
**Outcome(s)**<br><br>**Variable(s)**	- Increased DM levels<br>- Increased levels of empathy<br>- Reduced anxiety
**Outcome(s) Measures**	Interpersonal Responsiveness Index (IRI)<br>Mindful Attention Awareness Scale (MAAS)<br>State Trait Anxiety Inventory (STAI)
**Any adverse events related to MBIs interventions**	no adverse events occurred. Recruits were briefed on potential risks and provided with contact information for mental health services as a precaution.
**Facilitator(s)**	NS<br>book and cd by Sharon Salzberg's Real Happiness: The Power of Meditation: A 28-Day Program book and audio CD
**Facilitator's competencies**	NS<br>Sharon Salzberg is a meditation teacher
**Context (e.g.: course year, theoretical lessons; internship)**	Extracurricular at home
**Finding**	The data show that a detectable decrease in anxiety levels can result from participating in a self-directed mindfulness program of as little as four weeks. The results related to levels of awareness and empathy were less conclusive. A specific relationship between empathy and awareness cannot be determined

**Limits**	Convenience sample; non-generability to the student population in inferm.ca. Reduced sample size.. Self-administered reports. Prevalence of women.
**If unclear content, the author of the correspondence has been contacted**	NP
**He replied within the indicated time (one week)**	-
**Reference** [72]	Leggett, 2010
**Country**	USA
**Study Design**	RCT/(D/T)
**Sample Size**<br><br>**Dropout score**	90 nursing students who attended the first academic year – Nursing Degree<br>E and C: not specified<br>*Dropout rate*<br>85 (6%) E: 42; C:43
**MBIs applied**	I: brief MM intervention: brief breathing intervention and debriefing C: usual treatment<br><u>*Description of the intervention.*</u><br>MINDFUL BREATHING SCRIPT<br>Welcome & Introduction<br>Welcome to this mindful breathing program. Breathing is a phenomenon that occurs regularly without attention on our part. Our purpose in these three sessions in this class is on managing stress through mindful breathing by paying attention to our breath from moment to moment. Research suggests that deep breathing actually induces stress-releasing neurotransmitters.<br>So, being more mindful in deep breaths can reduce stress in a difficult clinical situation.<br>Step 1: Preparation<br>Before proceeding, make sure that your physical needs such as thirst, hunger or toilets are managed. Find a quiet spot with comfortable seating.<br>Step 2: Notice but don't change your breathing<br>Sit back and watch your breathing. Pay attention to your breathing<br>how it enters and how it exits. Don't force your breathing, simply observe each breath as it moves in and as it moves out. Track the air and observe the sensation of the breath for the entire duration of the inhale. Become aware of the entire duration of your breathing.<br>Now become aware of the entire duration of your breathing. Observe the act of breathing from moment to moment as the air slowly moves in and out. As the air moves, notice where it is felt, inside the nose and beyond, as the air progressively enters. What<br>The inhalation comes to an end, note the brief pause that occurs before the exhalation.<br>Again, pay attention to where you feel the air moving as it is exhaled. There will be another short break before the inhalation starts again. These pauses occur with every inhale and every exhalation. They are so short that you may not be aware of them, but by paying attention and being aware of the breath, you can notice them.<br>Continue to watch each breath as it enters and as it exits, predictable and constant. Pay attention to chest movements with each inhale and each exhalation. Notice any movement in your abdomen as you watch each breath from one moment to the next.

	moment with awareness. Does the chest expand? Does the chest contract? Is it the abdomen that expands with inhalation? Does the abdomen contract with exhalation? Notice the pattern of the breath. Is it a short breath or a long breath? At first, as you watch your breath, it can be brief. As calm sets in, watch your breath stretch with each inhale and exhale. Observe the ability of the breath to bring calmness throughout the body.<br>Experiencing shortness of breath<br>it can bring discomfort.<br>Step 3: Practice Long Breath<br>Practice making your breath long.<br>Step 4: Be present<br>As you observe your breath, you may find that your mind wanders to another task. Don't chastise yourself, simply turn your attention to the complete breathing cycle from moment to moment. The mind is wonderful and will do the task for which it<br>has been trained, which is thinking. Thinking is not bad, but it can take you away from the task of mindful breathing. Return to awareness of the breath and the act of breathing, allowing your body to relax and be supported as you become aware of the<br>breathe, inhale and exhale slowly. When the thoughts come in, let them pass and simply return to the breath. There is no need to dwell on thoughts, simply accept their presence, let them pass and return to the awareness of the breath.<br>Step 5: Clinical and Home Practice<br>This practice allows you to be present in the here and now by being aware of your breathing cycle moment by moment. Notice the feeling of relaxation you get with mindful breathing. You should practice mindful breathing for about 10 to 15 years<br>minutes per day. As you continue each day with this, you will find yourself becoming more aware of the things around you moment by moment. Congratulations on your Growing awareness experience!<br><br>DEBRIEFING SCRIPT<br>Students will be asked the following debriefing questions as part of the curricular protocol to help them internalize and generalize their learning to other personal stress situations. These questions will be presented orally in groups and<br>discussed but are not part of the data collection. These sessions will be audio-recorded.<br>The debriefing questions are listed below:<br>1. What has been your experience with mindful breathing?<br>2. How does mindful breathing work?<br>3. What happened while you were participating in mindful breathing?<br>4. How do you feel about mindful breathing?<br>5. How do you feel about the possibility of using it as stress reduction during stressful clinical experiences?<br>6. How could you apply this mindfulness breathing to stressful clinical situations and personal experiences?<br>7. What is it about mindfulness breathing that allows you to focus on specific nursing tasks or skills such as dressing changes or catheter insertions?<br>8. What insights did you gain during mindful breathing?<br>9. How can mindfulness breathing help you in your skills during clinical labs such as administering medications, changing dressings, or inserting catheters?<br>10. Mindfulness breathing is something that can help you in other areas<br>your life?

	Please record each daily 20-minute mindfulness breathing practice during the week.<br>Take it to your clinical lab and give it to your instructor. The logs are color-coded and labeled for each week: Week 1 = yellow, Week 2 = green, Week 3 = orange, Week 4 = pink, and Week 5 = lavender.
**MBIs duration**	A session of 1. 30 hours per week for three weeks
**Mode of Delivery**	In presence with daily practice of 20 minutes at home
**Outcome(s)**<br><br>**Variable(s)**	- Reduced stress level<br>- Reduction in the level of depression<br>- Increased perceived level of self-efficacy<br>- Increased level of dispositional mindfulness<br>- Evaluation of the effects on the activity of the autonomic nervous system (pA and HR)
**Outcome(s) Measures**	Center for Epidemiologic Studies Depression Scale (CESD-R)<br>Mindfulness Attention Awareness Scale (MAAS)<br>Student Clinical Completion Appraisal form<br>Clinical Skills Evaluation
**Any adverse events related to MBIs interventions**	NS
**Facilitator(s)**	Experienced Mindful Breathing Facilitator
**Facilitator's competencies**	NS
**Context (e.g.: course year, theoretical lessons; internship)**	Ad hoc workshops
**Finding**	Analyses of variance demonstrated a greater mean decrease for the intervention compared to the treatment group as usual after mindfulness breathing intervention for depression, $F(1.82) = 6.864$, $p = 0.010$; systolic, $F(1.81) = 6.557$, $p = 0.012$;<br>and diastolic, $F(1.81) = 6.078$, $p = 0.016$ — measures indicating that the intervention may be beneficial.<br>The heart rate results did not reach statistical significance.<br>Analysis of variance for awareness, self-efficacy and skill performance did not reach significance.<br>Correlations conducted on the measures indicated that, as depression decreased, systolic and diastolic measures also decreased. As awareness has increased, self-efficacy has increased, and skill performance has also increased.
**Limits**	This study was limited to a geographic area with little ethnic diversity of the sample, which may influence generalization to other populations. small sample size due to Drop-out (NS)
**If unclear content, the author of the correspondence has been contacted**	NP

**He replied within the indicated time (one week)**	-
**Reference** [73]	Lynch et al., 2023
**Country**	USA
**Study Design**	QE (D/T)
**Sample Size**<br><br>**Dropout score**	122 nursing students<br>Dropout rate: 56%<br>- 68
**MBIs applied**	The intervention is chosen from the web and include 4 short written meditations with a focus on the stressors encountered by undergraduate nursing students: a one-minute mindful breathing meditation, a three-minute body scan, and two image-guided meditations, one for 5 minutes and one for 7 minutes.
**MBIs duration**	NS
**Mode of Delivery**	Online
**Outcome(s)**<br><br>**Variable(s)**	- Reduced stress level<br>- Increased level of resiliency
**Outcome(s) Measures**	ad hoc survey on stress and anxiety and on the usefulness of short MBIs administered by the website
**Any adverse events related to MBIs interventions**	NS
**Facilitator(s)**	NS
**Facilitator's competencies**	NS
**Context (e.g.: course year, theoretical lessons; internship)**	NS
**Finding**	The results of the survey showed that students found the website easily accessible and useful.<br>Student satisfaction is highlighted
**Limits**	time to devote to practice.
**If unclear content, the author of the correspondence has been contacted**	NP
**He replied within the indicated time (one week)**	-

**Reference** [74]	Medari, 2023
**Country**	USA.
**Study Design**	MM (D/T)
**Sample Size**<br><br>**Dropout score**	102 nursing Students<br>*Dropout rate*<br>14 (86%)
**MBIs applied**	MindShift® CBT App from device.<br>https://www.youtube.com/embed/0Ka10cf9dSY?feature=oembed
**MBIs duration**	10 minutes five times a week for six weeks
**Mode of Delivery**	Online Application
**Outcome(s)**<br><br>**Variable(s)**	- Increased perceived level of self-efficacy<br>- Reduced stress level
**Outcome(s) Measures**	Pss Perceived Stress Scale<br>General Self-Efficacy Scale
**Any adverse events related to MBIs interventions**	NS
**Facilitator(s)**	NS<br>Through App
**Facilitator's competencies**	NS
**Context (e.g.: course year, theoretical lessons; internship)**	Semester
**Finding**	Not statistically significant differences. A downward trend in perceived stress and an increasing trend in self-efficacy indicate the clinical relevance of providing this tool, particularly for first-semester students. students suggest continuing to use it.
**Limits**	The small sample size does not allow the generability of the results. The lack of randomization. Inconstancy in using the app. I would also add privacy because it is interactive, students write what they feel about constructs such as worries, fears, etc... (see link and explanation of the app *ed.)*
**If unclear content, the author of the correspondence has been contacted**	NP
**He replied within the indicated time (one week)**	-
**Reference** [75]	Oseguera, 2019
**Country**	USA

**Study Design**	MM (D/T)
**Sample Size**<br><br>**Dropout score**	60 nursing students for the quantitative phase<br>15 nursing students for the qualitative phase
**MBIs applied**	Mindfulness Meditation (deep breathing) The Mindful Movement on YouTube<br>https://www.youtube.com/watch?v=erj8eaD_OmM
**MBIs duration**	Five minutes a day for four weeks
**Mode of Delivery**	In-person with YouTube online video
**Outcome(s)**<br><br>**Variable(s)**	- Reduced stress level<br>- Describe the experiences of Mm (QUAL DOM 1)<br>- Describe their stress levels (QUAL DOM 2)
**Outcome(s) Measures**	Cognitive and Affective Mindfulness Scale-Revised (CAM-R);<br>Perceived Stress Scale (PSS)
**Any adverse events related to MBIs interventions**	NS
**Facilitator(s)**	The teachers
**Facilitator's competencies**	Prepared by the researcher (NS The course protocol)
**Context (e.g.: course year, theoretical lessons; internship)**	In the first five minutes of the theory lesson
**Finding**	The results of the quasi-experimental research showed a statistically significant decrease in stress levels and an increase in awareness among nursing students who participated in the study.<br>QL Question 1: (a) positive experience, (b) calmer and more relaxed feeling, (c) meditation too short, (d) decreased stress, and (e) pleasant meditation experience.<br>QL Question 2: (a) stress levels decrease, (b) too short in time and different position in time, (c) calmness in breathing, (d) cleared mind and higher concentration level
**Limits**	Drop-out during practice; Reasons: absenteeism, illness or other reasons. Convenience sample; auto-report; inexperience of the interviewers
**If unclear content, the author of the correspondence has been contacted**	NP
**He replied within the indicated time (one week)**	-
**Reference** [76]	Ross, 2022
**Country**	USA
**Study Design**	QE (D/T)

**Sample Size**<br><br>**Dropout score**	79 nursing students who attended the 1st academic year- Nursing Degree<br>Dropout rate: 63%<br>-50
**MBIs applied**	Modified MBSR, based on meditations such as walking, eating, and communicating mindfully.<br>*Description of the intervention.*<br>**Session 1.**<br>Facing challenges.<br>Introduction to MBSR concepts; the opening circle; raisin meditation.<br>**Session 2.**<br>Perception and creative response.<br>Sitting meditation<br>**Session 3.**<br>Pleasure, the power of being present.<br>Mindful movement: yoga stretches; the gratitude sheet .<br>**Session 4.**<br>Stress reactivity and the role of mindfulness.<br>Sitting meditation and the non-grateful sheet.<br>**Session 5.**<br>Responding versus reacting: you are not your thoughts.<br>Seated meditation; focus attention on objects<br>**Session 6.**<br>Working with difficult situations including stressful communication.<br>Seated meditation; conscious walking.<br>**Session 7.**<br>Integrate the practice of Mindfulness fully and personally into daily life.<br>Seated meditation; Health promotion<br>**Session 8**.<br>The eighth week is the rest of your life.<br>Seated meditation; closing circle; Reflections.
**MBIs duration**	15-minute sessions before the start of the theoretical course, one session per week (7:35 am to 7:50 am; lesson start time 8:00 am)
**Mode of Delivery**	In presence with a request for home activities for 10-30 minutes a day
**Outcome(s)**<br><br>**Variable(s)**	- Reduced stress level<br>- Reduced anxiety level
**Outcome(s) Measures**	Short Stress Overload Scale (SOS-S)<br>Perceived Stress Scale (PSS)
**Any adverse events related to MBIs interventions**	NS
**Facilitator(s)**	The first researcher under the supervision of the course teacher who had an interest in Mm
**Facilitator's competencies**	NS

**Context (e.g.: course year, theoretical lessons; internship)**	During the first semester before the begin of the course
**Finding**	Comparison of pre- and post-stress scores was not statistically significant. The impact on students has been positive (calmer and more engaged); implemented the practices at home
**Limits**	Convenience sample. Important Drop-out that surrendered the very small sample size.
**If unclear content, the author of the correspondence has been contacted**	NP
**He replied within the indicated time (one week)**	-
**Reference** [77]	Simonton, 2021
**Country**	USA.
**Study Design**	MM (D/T)
**Sample Size**<br><br>**Dropout score**	57 nursing students<br>Dropout rate: -41(72%)
**MBIs applied**	Koru of Mindfulness developed by Rogers and Maytan (2012): (belly breathing/breath awareness, dynamic breathing, body scanning, walking meditation, Gatha meditation, guided imagery, thought/feeling labeling)
**MBIs duration**	5-10 minutes of MK per day for five weeks
**Mode of Delivery**	Online on Canva platform
**Outcome(s)**<br><br>**Variable(s)**	- Reduced level of anxiety from high-intensity simulation<br>- Description of experiences on KD<br>- Description of how the COVID period has affected the use of Mindfulness-based interventions
**Outcome(s) Measures**	State-Trait Anxiety Inventory (STAI) Daily logs: mindfulness strategy used; number of minutes practiced.
**Any adverse events related to MBIs interventions**	NS
**Facilitator(s)**	NS
**Facilitator's competencies**	NS
**Context (e.g.: course year, theoretical lessons; internship)**	Before High-Fidelity Simulations

**Finding**	Quantitative data are scarce.<br>QL data: In summary, participants perceived the value of mindfulness techniques as a coping strategy and envisioned using them as part of nursing practice for personal use (coping with work stress, increasing focus) and professional use (integrating them into patient care plans when appropriate). The sense of feeling overwhelmed is linked to three main sub-themes: curricular changes, the inability to see peers/faculty, and the fear related to illness/falling behind in studies.
**Limits**	The study does not allow for generalization of the results, as it was conducted at a single university in the Northeast, with a sample size of 16 participants.
**If unclear content, the author of the correspondence has been contacted**	NP
**He replied within the indicated time (one week)**	-
**Reference** [78]	Tenrreiro, 2022
**Country**	USA
**Study Design**	QE pre/post-test (D/T)
**Sample Size**<br><br>**Dropout score**	20 nursing students<br><br>Dropout rate not specified
**MBIs applied**	MBSR<br>NS Protocol
**MBIs duration**	NS
**Mode of Delivery**	NS
**Outcome(s)**<br><br>**Variable(s)**	Reduction of the level of exam anxiety
**Outcome(s) Measures**	Cognitive Test Anxiety Scale (CTAS-2)
**Any adverse events related to MBIs interventions**	NS
**Facilitator(s)**	NS
**Facilitator's competencies**	NS
**Context (e.g.: course year, theoretical lessons; internship)**	NS
**Finding**	CTA management can help with nursing student retention and address the shortage of nurses.

**Limits**	Threats to the internal, external and statistical validity of the conclusions. Most of the limitations are related to the design and methodologies of the research.
**If unclear content, the author of the correspondence has been contacted**	NP
**He replied within the indicated time (one week)**	-
**Reference** [79]	Teribury, 2021
**Country**	USA
**Study Design**	QE pre/post-test (D/T)
**Sample Size**<br><br>**Dropout score**	26 nursing students<br>Dropout rate: 12%<br>-3
**MBIs applied**	mindfulness meditation APP Headspace<br>https://www.headspace.com/headspace-meditation-app
**MBIs duration**	Eight weeks.<br>NS The time of each connection to the App
**Mode of Delivery**	Online through application support
**Outcome(s)**<br><br>**Variable(s)**	Reduction in the level of test anxiety
**Outcome(s) Measures**	Cognitive Test Anxiety Scale-2 (CTAS-2)
**Any adverse events related to MBIs interventions**	NS
**Facilitator(s)**	APP Headspace
**Facilitator's competencies**	NS
**Context (e.g.: course year, theoretical lessons; internship)**	During a semester
**Finding**	This descriptive interventional study investigated whether mindfulness Headspace meditation reduced cognitive test anxiety in nursing students. Data was obtained from 23 participants who completed the demographic survey, CTAS-2 pre- and post-test, and Mindfulness Meditation Log.<br>Data and statistical analysis showed that CTAS-2 scores were reduced when mindfulness meditation was used. There was no linear relationship in the difference between pre- and post-test scores and the number of minutes mindfulness meditation was performed;

	Therefore, the data and statistical analysis were not completed. Instead, the data was analyzed for a correlation with post-test CTAS-2 scores, and the number of minutes of mindfulness meditation was performed. However, a negative linear relationship was observed with post-test CTAS-2 scores and mindfulness meditation performance for 200-1400 minutes.
**Limits**	The dropout rates, lack of participation, truthful response to the online survey, and tracking the amount of time the mindfulness meditation was performed.
**If unclear content, the author of the correspondence has been contacted**	NP
**He replied within the indicated time (one week)**	-
**Reference** [80]	Tung, 2019
**Country**	China
**Study Design**	RCT
**Sample Size**<br><br>**Dropout score**	88 students<br><br>Dropout rate: 17,04%<br>E: -11; C:-4
**MBIs applied**	E: Mindful Self Compassion (MSC) programme<br>C: wait-list<br>_Description of the intervention._<br>MSC Program.<br>The table shows the topics related to the MSC presented to the intervention group; in addition, students were assigned homework related to the MSC.<br><br>MSC programme calendar and theme<br>Session 1: Discovering Conscious Self-Compassion<br>Session 2: Practicing mindfulness<br>Session 3: Practicing Loving-Kindness<br>Session 4: Discovering Your Compassionate Voice<br>Session R: Attending a mini-retreat<br>Session 5: Living Deeply<br>Session 6: Encounter with difficult emotions<br>Session 7: Exploring Difficult Relationships<br>Session 8: Embracing Your Life
**MBIs duration**	8 weeks with one session per week lasting 3 hours and a half-day retreat scheduled between the fourth and fifth sessions.
**Mode of Delivery**	In presence

**Outcome(s)**<br><br>**Variable(s)**	Influence of mindful self-compassion on mindfulness predisposition, participant self-compassion, compassion satisfaction and *compassion fatigue* and stress
**Outcome(s) Measures**	The scales were administered before the MSC program, after the first 4 weeks of the course, at the end of the course and after 4 weeks from the end.<br>- Five Facet Mindfulness Questionnaire - Chinese version (FFMQ-C)<br>- Self-Compassion Scale – Chinese (SCS-C)<br>- Chinese Professional Quality of Life scale (Chinese ProQOL-5)<br><br>Chinese Perceived Stress Scale (Chinese PSS)
**Any adverse events related to MBIs interventions**	NS
**Facilitator(s)**	Not the Principal Investigator
**Facilitator's competencies**	MSC Certified Teacher
**Context (e.g.: course year, theoretical lessons; internship)**	Nursing students. The year or period of the academic year in which the intervention was administered is not specified.
**Finding**	The MSC program has been shown to be effective in increasing levels of mindfulness and self-compassion while reducing *compassion fatigue*, burnout, and stress levels among nursing students.
**Limits**	- Convenience Sample<br>- Only Self-reported data were used to assess the effects of the intervention<br><br>High dropout
**If unclear content, the author of the correspondence has been contacted**	NN
**He replied within the indicated time (one week)**	NO
